# Site-Selective Functionalization of Unactivated Allylic C–H Bonds via Direct Deprotonation with KTMP: Application to the Formal Total Synthesis of (+)-Artemisinin from Amorphadiene

**DOI:** 10.1021/acs.orglett.2c04145

**Published:** 2023-01-02

**Authors:** Nicholas A. Clanton, Nicolas A. Wilson, Eliezer Ortiz, Shawn T. Blumberg, Doug E. Frantz

**Affiliations:** ‡ The Max and Minnie Tomerlin Voelcker Laboratory for Organic Chemistry, Department of Chemistry, The University of Texas at San Antonio, San Antonio, Texas 78249, United States; † Department of Pharmaceuticals & Bioengineering, Southwest Research Institute, San Antonio, Texas 78238, United States

## Abstract

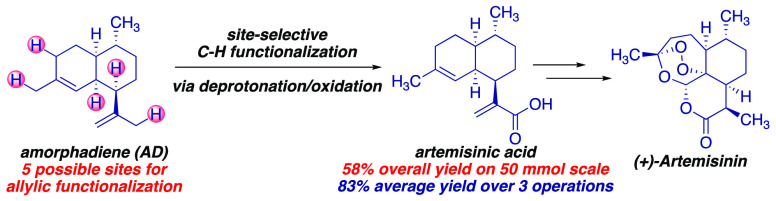

The site-selective functionalization of unactivated allylic C–H bonds via direct deprotonation using KTMP is described. The conversion of amorphadiene to artemisinic alcohol via a simple, highly regioselective deprotonation over 4 other possible allylic sites is shown with further extrapolation to the first large-scale telescoped chemical synthesis of artemisinic acid from amorphadiene. Finally, application of the method for the successful site-selective functionalization of unactivated allylic C–H bonds in other terpene-based natural products is also highlighted.

The latest World Malaria Report from the World Health Organization (WHO) estimated 241 million cases of malaria and 627,000 deaths in 2020.^1^ Unfortunately, these numbers represent a significant increase compared to 2019 and were exacerbated by disruptions in prevention, diagnosis, and treatment of malaria due to the ongoing COVID-19 pandemic. Further disruptions in global supply chains continue to negatively impact the production of antimalarials and ultimately contribute to reduced gains in malaria-endemic countries.

In the battle against malaria, artemisinin-based combined therapies (ACTs) remain as the first-line arsenal for the treatment of uncomplicated disease caused by *P. falciparum* and, in select cases, by *P. vivax*. Global demand for ACTs continues to grow, up to 218 metric tons (MT) in 2021;^2^ however, meeting this demand has been hampered not only due to COVID-19 but the long-term supply issues of artemisinin (**1**) itself. During the past 20 years, the world’s supply and cost of artemisinin has been notably erratic. Extraction from *Artemisia annua L.* continues to be the major source of this API (100–120 MT/year), yet sustainable supplies are dependent on varying market dynamics, climate change, geographical location, and geopolitical pressures. The remaining gap in artemisinin stock is supplemented by semisynthetic industrial approaches (50–60 MT/year) that rely on the biosynthetic production of artemisinic acid (AA, **5**) via fermentation of sugar in titers of ∼25 g/L using genetically engineered strains of *Saccharomyces cerevisiae* (brewer’s yeast).^3,4^ Significant effort has since been put forth to optimize the chemical transformation of AA to artemisinin. Despite these elegant strategies, the cost of semisynthetic approaches (350–400 $/kg) still do not compete with the current price of artemisinin obtained by extraction (250 $/kg).

Interestingly, the initial fermentation step used to produce amorph-4,11-diene (amorphadiene, AD, **2**) is capable of producing titers that are up to 5 times higher (∼120 g/L) than was obtainable for AA.^5^ Thus, realizing that AD could serve as a more attractive precursor to develop a semisynthetic route to artemisinin, Amyris developed two approaches to oxidize AD via either selective hydroboration/oxidation or epoxidation of the exocyclic double bond. However, both of these routes were abandoned and deemed too costly at the time.^6^ Renewed efforts to utilize AD involved a 6-step synthetic sequence beginning with selective epoxidation of the endocyclic double bond allowing for subsequent manipulation of the exocyclic olefin that ultimately culminated with a Li-metal mediated reductive removal of the epoxide to provide dihydroartemisinic acid (DHAA).^7^ However, the implementation of this route on production scale, to the best of our knowledge, has yet to occur.

Biomimetic approaches that target intermediates along biosynthetic pathways are proven strategies for the total synthesis of natural products. From this perspective, the development of a direct chemical conversion of AD to artemisinic alcohol (**3**), the next intermediate on the biosynthetic pathway to artemisinin, could provide significant advantages over previous semisynthetic approaches. In turn, this strategy would complement existing total syntheses of artemisinin that start from commodity raw materials, most notable of these being the graceful approach developed by Cook.^8^ Captivated by the recent success of the Cossy and Amara groups on the functionalization of AD,^9−11^ in particular the Pd-catalyzed regioselective oxidation of AD,^12^ we embarked on our own efforts to identify a robust and efficient approach for the direct conversion of AD to artemisinic alcohol as a biomimetic formal total synthesis to artemisinin (Scheme 1).

**Scheme 1:**
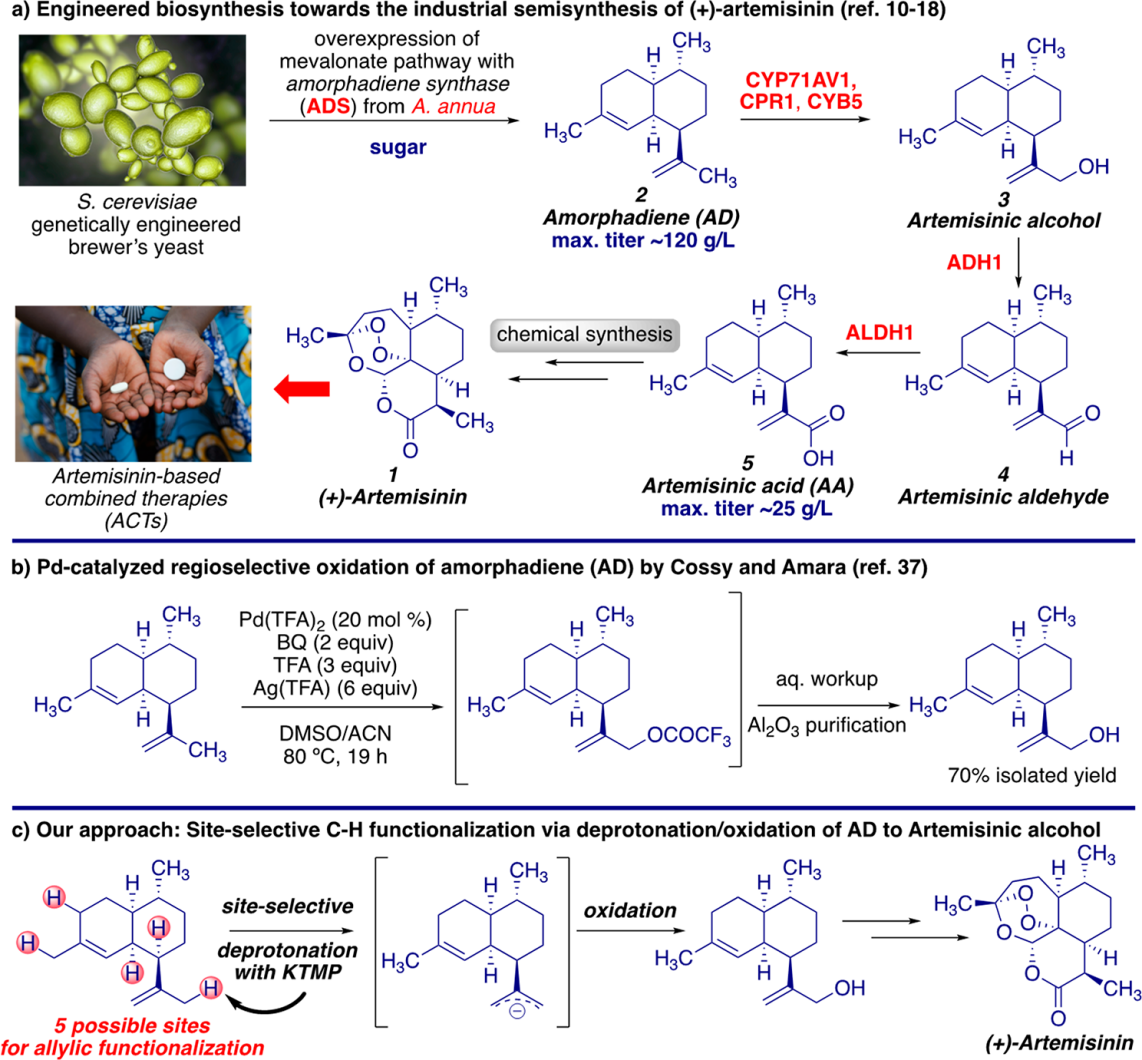
(a) Engineered Biosynthesis toward the Industrial Semisynthesis of (+)-Artemisinin; (b) Pd-Catalyzed Regioselective Oxidation of Amorphadiene (AD) by Cossy and Amara; (c) Our Approach: Site-Selective C–H functionalization via Deprotonation/Oxidation of AD to Artemisinic Alcohol

Direct chemoselective and site-selective C–H functionalization of complex naturally occurring sesquiterpenes, such as AD, remains a formidable challenge for synthetic organic chemists. From this perspective, in an age where catalytic C–H functionalizations are making great strides, simple and efficient stoichiometric functionalizations can sometimes be overlooked. For example, regioselective stoichiometric deprotonations have been a proven strategy for the functionalization of unsaturated hydrocarbons, including terpenes, since the 1970s. The landmark study by Crawford on the direct metalation of limonene using *n-*BuLi-TMEDA catalyzed a series of subsequent investigations by others in the field.^13^ In the 1980s Schlosser’s “superbase” system^14,15^ that combined *n-*BuLi with KO^*t*^Bu (LICKOR) gained notoriety and was demonstrated in the selective allylic deprotonation of simple olefins and terpenoids.^16^ However, the application of these deprotonation strategies to more complex sesquiterpenes, including AD, has yet to be demonstrated.

A selected subset of our initial exploratory reactions for the selective deprotonation of AD followed by borylation/oxidation is presented in Table 1 (see Supporting Information for more details). Preliminary proof of concept was realized using *n-*BuLi-TMEDA in hexanes; conditions previously shown to metalate limonene.^13^ However, conversion of AD to **3** was low with concomitant formation of isomeric allylic alcohol **6** (Table 1, entries 1 and 3). Even lower conversion was realized using a *s*-BuLi-TMEDA combination (Table 1, entry 2). Significant conversion was not realized until the ternary combination of *n*-BuLi, KO^*t*^Bu, and 2,2,6,6-tetramethylpiperidine (TMP) in THF was employed (Table 1, entry 7). Additional optimization of this result using 2 equiv of each reagent at −78 °C provided high conversion of AD (89%), exceptional site-selectivity (>20:1), and an excellent isolated yield of **3** (81%) (Table 1, entry 9). It is important to note that proven methods for allylic deprotonations of terpene-based natural products using alkyl potassium superbases (e.g., Schlosser’s LICKOR conditions) completely failed to deprotonate AMD (Table 1, entry 5). Furthermore, reactions without KO^*t*^Bu (generating LiTMP) or *n-*BuLi alone, failed to provide any measurable conversion by ^1^H NMR (Table 1, entries 4 and 6). These data taken together provide supporting evidence that KTMP is required to achieve high conversion and regioselectivity for the deprotonation of AMD. As far as we are aware, this represents the first example of a regioselective allylic deprotonation using KTMP.^17^ To address the challenges of using cryogenic temperatures on an industrial scale, we explored reaction conditions using KTMP in hydrocarbon solvents where increased thermal stability is known over ethereal solvents.^18^ We were pleased to realize promising conversion (68%) and site-selectivity (3:1) using heptane as the solvent at room temperature using 3 equiv of KTMP (Table 1, entry 12). Alternatively, the use of continuous flow conditions could also be employed, as demonstrated by Knochel.^19^

**Table 1:**
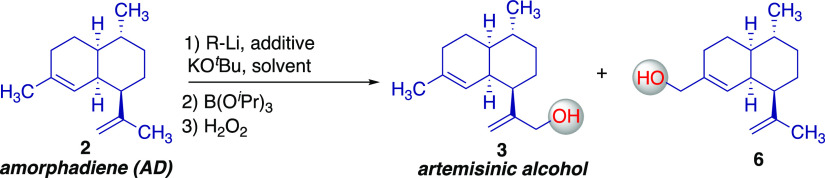
Initial Exploration of the Regioselective Deprotonation/Oxidation of Amorphadiene

entry^a^	R-Li (equiv)	additive (equiv)	KO^*t*^Bu (equiv)	solvent	temp (°C)	time (h)	conversion of AD (%)^b^	yield (%)^c^	**3**:**6**^d^
1	*n*-BuLi (0.67)	TMEDA (0.67)	none	hexanes	0	16	35	ND	3:1
2	*s*-BuLi (0.67)	TMEDA (0.67)	none	cyclohexane	0	16	6	ND	5:1
3	*n*-BuLi (1.0)	none	1.0	hexanes	0	24	25	ND	2:1
4	*n*-BuLi (1.0)	none	none	THF	–78	1	<5	ND	ND
5	*n*-BuLi (1.2)	none	1.2	THF	–78	1	<5	ND	ND
6	*n*-BuLi (1.2)	TMP (1.2)	none	THF	–78	1	<5	ND	ND
7	*n*-BuLi (1.2)	TMP (1.2)	1.2	THF	–78	1	72	60	>20:1
8	*n*-BuLi (1.5)	TMP (1.5)	1.5	THF	–78	1	78	72	>20:1
9	*n*-BuLi (2.0)	TMP (2.0)	2.0	THF	–78	1	**89**	**81**	**>**20:1
10	*n*-BuLi (2.0)	TMP (2.0)	2.0	THF	–40	1	<5	ND	ND
11	*n*-BuLi (2.0)	TMP (2.0)	2.0	THF	–25	1	<5	ND	ND
12	*n*-BuLi (3.0)	TMP (3.0)	3.0	heptane	23	24	68	39	3:1

a All reactions performed on a 0.5 mmol scale of AD at 0.1 M concentration with 1 equiv of B(O^*i*^Pr)_3_ and 2 equiv of H_2_O_2_ relative to *n*-BuLi.

b Determined via ^1^H NMR analysis of crude reaction mixtures by comparing the relative amounts of AD to **3** and **6** combined.

c Isolated yields.

d Ratios determined on crude reaction mixtures by ^1^H NMR.

With a highly regioselective and robust deprotonation/oxidation of AD in hand, we turned our attention to developing a process to convert AD to artemisinic acid (**5**) without isolation or purification of any intermediates. After minimal rounds of optimization, we have identified a two-step/one-pot oxidation sequence using crude artemisinic alcohol (**3**) obtained from the regioselective deprotonation to provide **5** in high isolated yield on a 50 mmol scale (Scheme 2). Regioselective deprotonation/oxidation of AD using KTMP as described above provided artemisinic alcohol **3** in 86% conversion with 24:1 artemisinic alcohol **3** in 86% conversion with 24:1 regioselectivity as determined by ^1^H NMR analysis. Oxidation of crude **3** to artemisinic aldehyde **4** was realized via a Cu-catalyzed oxidation using O_2_ as the stoichiometric oxidant in >99% conversion as determined by ^1^H NMR after 1 h. Subsequent conversion of **4** to **5** was achieved in 71% HPLC assay yield from AD via a Pinnick oxidation using 2-methyl-2-butene (15 equiv) as the scavenger for the HOCl byproduct. Isolation via direct crystallization from acetonitrile/H_2_O provided **5** in 58% overall yield from AD resulting in an average yield of 83% for each of the three steps. Downstream industrial conversion of artemisinic acid (AA) to (+)-artemisinin is a highly optimized route capable of producing 50–60 tons/year in ∼55% overall yield, thus completing the formal total synthesis route from AD.^20^

**Scheme 2:**
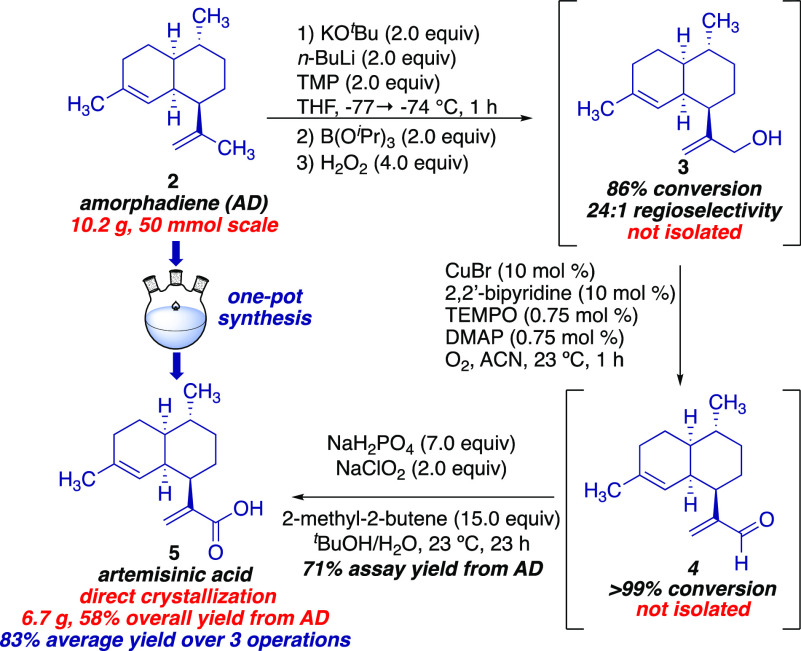
Through Process for the Conversion of AD to AA on 50 mmol Scale

Intrigued by the highly site-selective deprotonation of AD enabled by KTMP, we were curious if this method could be used to selectively functionalize unactivated allylic C–H bonds in other terpene-related natural products. A summary of our preliminary efforts is provided in Scheme 3 (see Supporting Information for experimental details on each example). For example, the robustness of the method allows for the direct site-selective C–H functionalization of (+)-limonene contained in naturally occurring orange oil (∼$1,650/55 gallons) to provide (+)-limone-10-ol (**7**) in 77% yield. The related cyclic monoterpene α-phellandrene is selectively functionalized at the exocyclic allylic position to provide cuminyl alcohol (**8**) in 48% yield after unavoidable aromatization during workup. Selective deprotonation/oxidation of (−)-perillyl alcohol provides (−)-10-hydroxyperillyl alcohol (**9**) in 70% yield that, as far as we are aware, has only been achievable using biotransformation approaches in the past.^21^ Likewise, the regioselective functionalization of (+)-β-citronellene yields (+)-(*Z*)-allylic alcohol **10** in (86:14, *Z:E*); a functionalized enantiopure monoterpene that has remained elusive until now. In a similar fashion, the site-selective deprotonation/oxidation of (±)-β-citronellol provides direct access to (±)-(*Z*)-8-hydroxycitronellol (**11**, 91% yield, 84:16, *Z:E*), a precursor to an HIV protease inhibitor, that previously required 5 steps to synthesize from (±)-citronellal.^22^ The *Z*-selectivity obtained for both **10** and **11** is consistent with the thermodynamic preference for allylic potassium species to exist in the *endo*-Z conformation.^23^ KTMP can also be used to selectively hydroxylate (−)-isopulegol to provide diol **12** in 93% isolated yield. The site-selective oxidation of natural grade (+)-valencene (74% purity, $780/kg) to provide valencen-13-ol (**13**) in 77% yield represents the most direct and highest yielding method to this known tick repellent.^24^ Finally, we were elated to realize the site-selective C–H functionalization of cannabidiol (CBD) to provide 10-hydroxy-CBD (**14**) in an impressive 52% isolated yield. Previous SAR studies on CBD at the 10-position have demonstrated these derivatives can block the antinociceptive activity of Δ^9^-THC.^25^

**Scheme 3:**
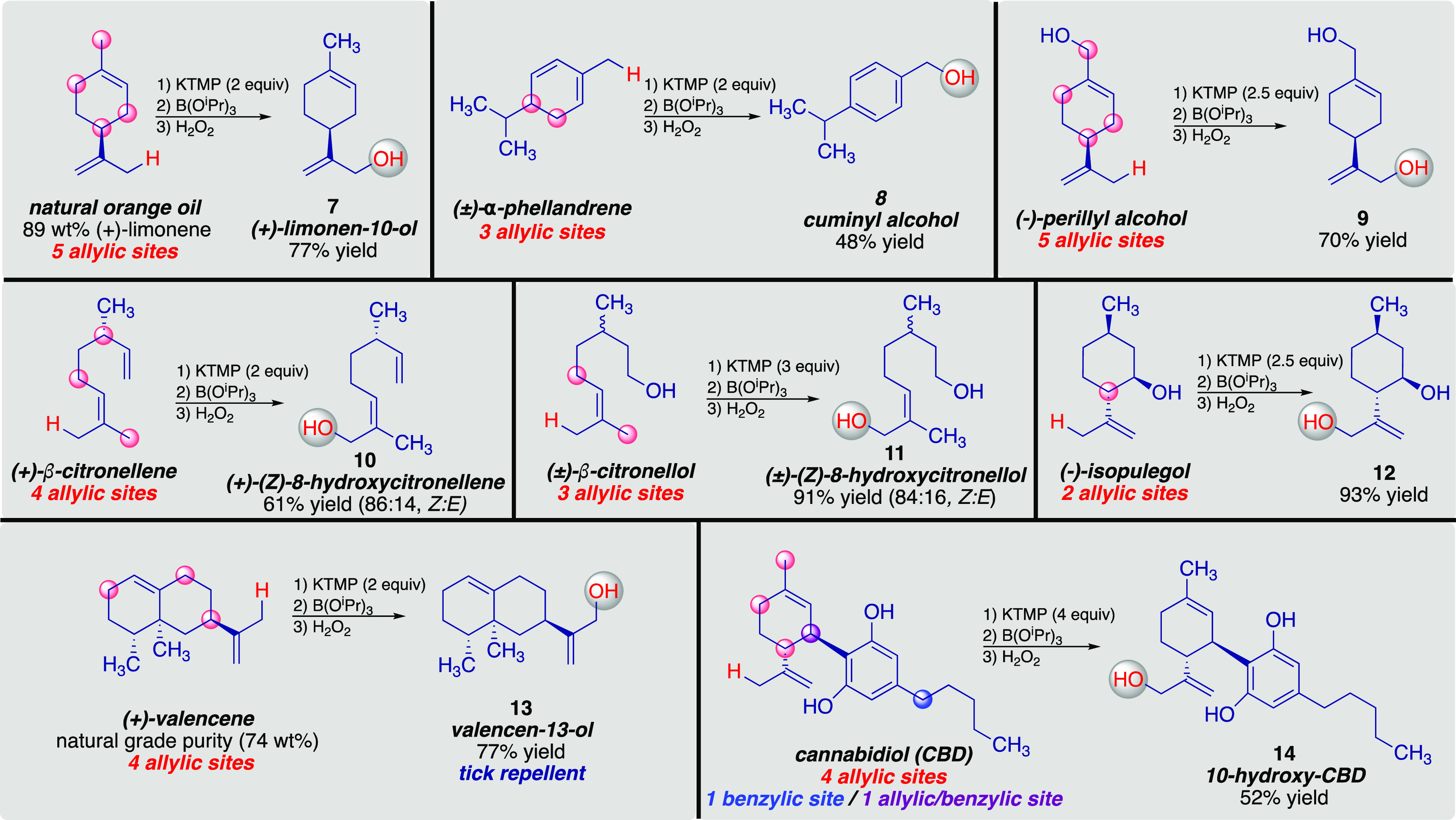
Site-Selective C–H Functionalization of Terpene-Based Natural Products via KTMP Deprotonation/Oxidation

In conclusion, we have discovered and developed a direct allylic C–H functionalization of amorphadiene (AD) to artemisinic alcohol via a highly regioselective deprotonation. Critical to the success of this reaction is the use of KTMP as the preferred base that demonstrates superior regioselectivity for deprotonation at C12 over 4 other possible allylic sites in AD. Extrapolation of this method to the first telescoped chemical synthesis of artemisinic acid from amorphadiene with implications for the large-scale semisynthetic production of artemisinin is also demonstrated on a 50 mmol scale. Finally, extension of this method to the site-selective allylic C–H functionalization of other terpene-based natural products was also explored.

## Data Availability

The data underlying this study are available in the published article and its online Supporting Information.

## Supporting Information Available

Full experimental details and characterization, ^1^H and ^13^C NMR spectra of all final products (PDF)
