# Supplementary material for: Site-Selective Functionalization of Unactivated Allylic C–H Bonds via Direct Deprotonation with KTMP: Application to the Formal Total Synthesis of (+)-Artemisinin from Amorphadiene
Source: Org Lett. 2023 Jan 2;25(1):277–81. doi: 10.1021/acs.orglett.2c04145 (PMC9841608; doi:10.1021/acs.orglett.2c04145)

Supporting Information

Site-Selective Functionalization of Unactivated Allylic C-H Bonds via Direct Deprotonation with KTMP: Application to the Formal Total Synthesis of (+)-Artemisinin from Amorphadiene

Nicholas A. Clanton,† Nicolas A. Wilson,† Eliezer Ortiz,† Shawn T. Blumberg,‡ Doug E. Frantz*,†

doug.frantz@utsa.edu

†The Max and Minnie Tomerlin Voelcker Laboratory for Organic Chemistry, Department of Chemistry, The University of Texas at San Antonio, San Antonio, Texas 78249, United States

‡Department of Pharmaceuticals & Bioengineering, Southwest Research Institute, San Antonio, Texas, 78238

Table of Contents

1. General experimental information S2
2. Synthetic procedures S2
   a. Method A: Deprotonation of allylic C-H bonds by KTMP in THF S2
   b. Deprotonation of amorphadiene (AD) with KTMP in heptane S2
   c. Deprotonation of AD with alkyllithium and TMEDA S3
   d. Deprotonation of AD with *n*BuLi and KO^{t}Bu in hexanes S3
   e. Small-scale oxidation of artemisinic alcohol to artemisinic acid S3
   f. 50 mmol-scale process for the direct conversion of AD to artemisinic acid (AA) S4
3. Characterization data on artemisinin related products S6
4. Site-selective functionalization of terpene-based natural products S7
5. Modified synthetic approach to amorphadiene S11
6. References S14
7. NMR Spectra S16

1. General Information: All reactions were performed under an atmosphere of nitrogen unless otherwise noted. Reaction progress was monitored by TLC and/or HPLC analysis. TLC analysis was performed on Silica Gel 60, aluminum-backed TLC plates. LC/MS analysis was performed on an Agilent 1200/6130 Series LC/MS equipped with a ZORBAX Eclipse XDB-C18 analytical column from Agilent (4.6 x 150 m, 5 µm, Part #: 993967-902). Quantitative HPLC analysis was performed on an Agilent 1260 instrument also equipped with a ZORBAX Eclipse XDB-C18 analytical column from Agilent (4.6 x 150 m, 5 µm, Part #: 993967-902). Column chromatography was performed using 230-400 Mesh silica gel. ^{1}H and ^{13}C NMR analyses were performed on a Bruker AVIII 500 MHz, or a Bruker AVIII 300 MHz NMR. All peaks reported as s (singlet), d (doublet), t (triplet), q (quartet), m (multiplet), qd (quartet of doublets), tq (triplet of quartets), ddd (doublet of doublets of doublets), ddt (doublet of doublets of triplets), ddq (doublet of doublets of quartets). HRMS analysis was performed on a Thermo Scientific Orbitrap Exploris 120 equipped with an ESI ion source or a quadrupole-time of flight (qTOF) mass spectrometer. All reagents were purchased from commercial sources without further purification unless otherwise stated. 2,2,6,6-tetramethylpiperidine (TMP) and *N,N,N'N'*-tetramethylethylene diamine (TMEDA) were distilled from potassium hydroxide prior to use. All solvents used in this study were dried and stored over 3Å molecular sieves. High purity solid potassium tert-butoxide (trace-metals basis, 99.99%) was purchased from Sigma-Aldrich, stored in the desiccator, and used without further purification. The concentration of *n*BuLi solution was determined according to the method of Ghiviriga et al.[1]

2. Synthetic procedures:

a. Method A: Deprotonation of allylic C-H bonds by KTMP in THF: A flame-dried round bottom flask under nitrogen and equipped with a magnetic stir bar and thermocouple was charged with THF (10.0 mL) and KO*t*Bu solution (1 M in THF, 2.0 mL, 2.0 mmol). The mixture was cooled to -78 °C and *n*BuLi (2.4-2.7 M in hexane, 2.0 mmol) was added dropwise while maintaining internal reaction temperature of < -72 ºC. The resulting yellow solution was stirred for 15 minutes at -78 °C. 2,2,6,6-tetramethylpiperidine (TMP, 0.34 mL, 2.0 mmol) was added dropwise while maintaining internal reaction temperature of < -72 ºC and then stirred for an additional 15 minutes at -78 °C. The terpene-based substrate (1.0 mmol) was then added via syringe and the reaction was stirred for 1 hour at -78 °C. Triisopropylborate (0.46 mL, 2.0 mmol) was added via syringe and the reaction was allowed to warm to room temperature over 30 minutes. Hydrogen peroxide (30% aqueous, 0.40 mL, 4.0 mmol) was added slowly to maintain the internal temperature < 30 ºC (**CAUTION: Exothermic step**). The reaction was stirred for an additional 30 minutes. The reaction is then diluted with water (20 mL) and transferred to a separatory funnel. The organic layer was extracted with 3 portions of MTBE (20 mL), washed twice with HCl (20 mL), and followed by brine (20 mL). The organic layer was dried over Na_2SO_4 and concentrated by rotary evaporation. The crude products were purified by flash silica gel chromatography.

b. Deprotonation of amorphadiene (AD) with KTMP in heptane: A flame-dried 10 mL Schlenk flask equipped with a magnetic stir bar was charged with potassium *tert*-butoxide (1.5 mmol). The flask was placed under high vacuum at 120 °C for 30 minutes, then was backfilled with nitrogen. Heptane (5 mL) was added, and the suspension was stirred for 15 minutes. *n*BuLi (2.5 M solution, 0.60 mL, 1.5 mmol) was added dropwise and the mixture was stirred for an

additional 15 minutes. TMP (0.26 mL, 1.5 mmol) was added dropwise, and the mixture was stirred for an additional 15 minutes. Over this time the suspension partially dissolved followed by the formation of a new precipitate. AD (102 mg, 0.50 mmol) was then added via syringe and the reaction was stirred for 24 hours at room temperature (~22-24 ºC). The flask was then cooled to 0 °C, triisopropyl borate (0.34 mL, 1.5 mmol) was added, and the reaction was stirred for an additional hour while allowing to warm back to room temperature. Hydrogen peroxide (30% aqueous, 0.16 mL, 1.5 mmol) was added slowly to maintain the internal temperature < 30 ºC (**CAUTION: Exothermic step**) and the resulting colorless solution was stirred for 15 minutes. The mixture was diluted with water (10 mL) and extracted with diethyl ether (3x10 mL). The organic layer was concentrated by rotary evaporation to afford the crude artemisinic alcohol **3**. Conversion and yield were determined by ^1H NMR analysis.

c. Deprotonation of amorphadiene (AD) with alkyllithium and TMEDA: A flame-dried 10 mL Schlenk flask equipped with a magnetic stir bar under nitrogen was charged with hexanes (0.3 mL) and *N,N,N',N'*-tetramethylethylenediamine (0.18 mmol). The solution was cooled to 0 °C and either a 2.5 M solution of *n*BuLi (0.27 mL, 0.67 mmol) of 1.6 M solution of *sec*-BuLi (0.42 mL, 0.67 mmol) and the mixture was stirred for 15 min, then AD (204 mg, 1.00 mmol) was added. The next day, the reaction was cooled to -40 ° and triisopropylborate (0.25 mmol) was added. After stirring for 1 h, 30% aqueous hydrogen peroxide (0.25 mmol) was added slowly to maintain the internal temperature < 30 ºC (**CAUTION: Exothermic step**) and stirred for an additional 15 min. The reaction was then worked up as described for the deprotonation with KTMP in heptane to afford the crude artemisinic alcohol **3**. Conversion and yield were determined by ^1H NMR analysis.

d. Deprotonation of amorphadiene (AD) with *n*BuLi and KO^{t}Bu in hexanes: A flame-dried flask equipped with a magnetic stir bar was charged with potassium *tert*-butoxide (224 mg, 2.0 mmol). The flask was placed under high vacuum at 120 °C for 30 minutes, then was backfilled with nitrogen. Hexanes (6 mL) was added, and the suspension was stirred for 15 minutes. The solution was cooled to 0 °C and a 2.55 M solution of *n*BuLi (0.78 mL, 2.0 mmol) was added and the mixture was stirred for an additional 15 min. AD (408 mg, 2.00 mmol) was added and the reaction was stirred for 24 hours. After stirring for 1 h, the reaction 30% aqueous hydrogen peroxide (0.25 mmol) was added slowly to maintain the internal temperature < 30 ºC (**CAUTION: Exothermic step**) and stirred for an additional 15 min. The reaction was then worked up as described for the deprotonation with KTMP in heptane to afford the crude artemisinic alcohol **3**.

e. Small-scale oxidation of artemisinic alcohol to artemisinic acid:

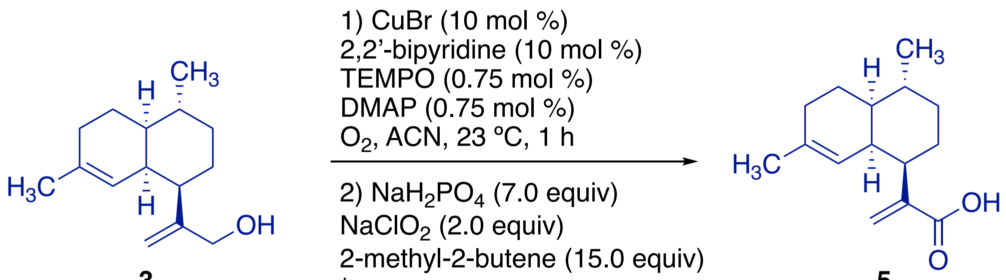

3 *artemisinic alcohol* → **5** *artemisinic acid*

To a round bottom flask equipped with a magnetic stirring bar was added, CuBr (14.6 mg, 10 mol %), 2,2'-bipyridine (15.6 mg, 10 mol %), TEMPO (1.17 mg, 0.75 mol %), and DMAP (1.83 mg, 0.75 mol%). The flask was charged with 7.5 mL of acetonitrile then evacuated and backfilled with oxygen for three cycles and placed under a balloon to maintain oxygen atmosphere. Artemisinic alcohol (220 mg, 1.00 mmol) was added via syringe, and the reaction was stirred until complete conversion to artemisinic aldehyde, observed by HPLC assay, (about 1 hour). At this time, the flask was opened to air, and *t*BuOH (7.5 mL), 2-methyl-2-butene (1.59 mL, 15.0 mmol), and a 0.865 M solution of NaH_2PO_4 (8.09 mL, 7 mmol) were added sequentially. A 1.28 M solution of $NaClO_2$ (1.56 mL, 2.00 mmol) was then added dropwise. The reaction was allowed to stir overnight at room temperature open to air, until judged complete by HPLC assay. Upon completion, the reaction was quenched by the addition of Na_2SO_3 (31.5 mg, 0.25 mmol) and stirred for an additional 5 minutes. The mixture was then diluted with saturated ammonium chloride. The aqueous layer was extracted with MTBE (3x10 mL). The combined organic extracts were washed with brine, dried over Na_2SO_4, and concentrated by rotary evaporation. The crude material then dissolved in acetonitrile (2 mL) and crystallized by the slow addition of water (2 mL). The solids were filtered and washed with additional water (1 mL) to afford the desired artemisinic acid as a white powder in 87% yield (224 mg).

f. 50 mmol-scale process for the direct conversion of amorphadiene (AD) to artemisinic acid (AA)

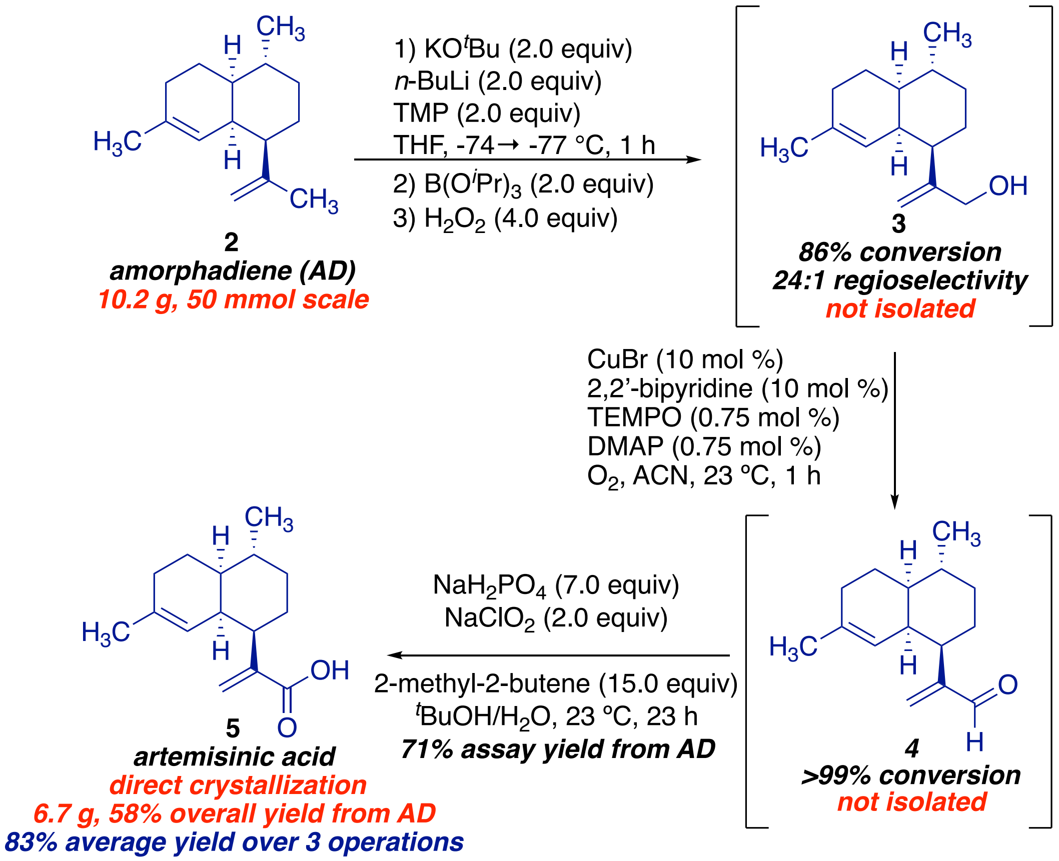

A flame-dried, 3-neck round bottom flask equipped with an addition funnel and thermocouple was placed under a nitrogen atmosphere and charged with 400 mL of THF. A solution of potassium *tert*-butoxide in THF (1 M, 100 mL, 100 mmol) was added and the flask was cooled to -77 °C (internal temperature). A solution of *n*BuLi in hexanes (2.83 M, 35.3 mL, 100 mmol) was added while maintaining an internal temperature of < -74 ºC and then allowed to stir for an additional 15 minutes. 2,2,6,6-tetramethylpiperdine (TMP, 14.23 g, 16.9 mL, 100 mmol) was added while maintaining an internal temperature of < -74 ºC and the resulting solution was also stirred for an additional 15 minutes. 4,11-amophadiene (amorphadiene (AD), 10.22 g, 50 mmol) was then added slowly via syringe (no noticeable temperature change during addition) and the reaction was stirred for an hour at -77 °C. After 1-hour, triisopropyl borate (18.18 g, 23.1 mL, 100 mmol) was added slowly and the flask was allowed to warm to room temperature over 30 minutes. The flask was then cooled to 0 °C and a 30% aqueous hydrogen peroxide (20.4 mL, 200 mmol) (**CAUTION: Exothermic step**) was added while maintaining an internal temperature of <15 °C and the reaction was stirred for an additional 30 minutes. After this time, the crude material was then diluted with water (100 mL) and extracted with MTBE in 3 portions (100 mL). The solution was then washed 2 times with 1M HCl (100 mL), dried over anhydrous sodium sulfate, and concentrated by rotary evaporation to yield the crude alcohol **3** (84% conversion by NMR). To a round bottom flask was added CuBr (731.9 mg, 10 mol %), 2,2'-bipyridine (781.0 mg, 10 mol %), TEMPO (58.59 mg, 0.75 mol %), and DMAP (91.63 mg, 0.75 mol%) along with a magnetic stir bar (all charges made based on starting 50 mmol of AD). The flask was then charged with acetonitrile (375 mL) and then evacuated and backfilled with oxygen for three cycles and ultimately placed under a balloon to maintain oxygen atmosphere. The crude alcohol **3** was added via syringe, and the reaction was stirred under oxygen atmosphere until complete conversion to artemisinic aldehyde **4** was observed by HPLC assay. At this time, the flask was opened to the air followed by the addition of *t*BuOH (375 mL), 2-methyl-2-butene (79.5 mL, 15.0 equiv.), and a 0.865 M solution of NaH_2PO_4 (404.6 mL, 7.0 equiv.) added sequentially. A 1.28 M solution of $NaClO_2$ (78.13 mL, 2.0 equiv.) was then added dropwise. The reaction was allowed to stir overnight at room temperature open to air, until judged complete by quantitative HPLC analysis. Upon completion, the reaction was quenched by the addition of Na_2SO_3 (1.576 g, 0.25 eq.) and stirred for an additional 5 minutes. The mixture was then diluted with saturated ammonium chloride. The aqueous layer was extracted with MTBE (3 x 200 mL). The combined organic extracts were washed with brine, dried over Na_2SO_4, and concentrated by rotary evaporation. The crude material then dissolved in acetonitrile (70 mL) and crystallized by the slow addition of water (70 mL). The solids were filtered and washed with additional water (22 mL) to afford the desired artemisinic acid **5** as white powder in 58% yield (6.74 g) from the starting amorphadiene.

Artemisinic Acid (5)[2]

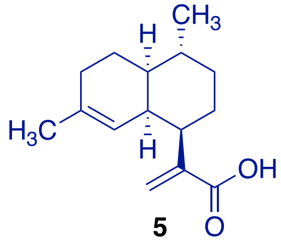

5
artemisinic acid

^{1}H NMR (500 MHz, $CDCl_3$) δ 11.17 (s, 1H), 6.47 (s, 1H), 5.58 (s, 1H), 5.01 (s, 1H), 2.72 (dt, *J* = 12.3, 3.8 Hz, 1H), 2.63 (s, 1H), 2.01 – 1.84 (m, 2H), 1.84 – 1.70 (m, 2H), 1.62 (s, 3H), 1.60 – 1.53 (m, 1H), 1.50 – 1.33 (m, 4H), 1.09 (qd, *J* = 12.3, 4.1 Hz, 1H), 0.92 (d, *J* = 5.8 Hz, 3H). ^{13}C NMR (126 MHz, $CDCl_3$) δ 171.8, 142.5, 135.0, 126.6, 120.1, 42.1, 41.4, 37.9, 35.2, 27.6, 26.4, 25.9, 25.6, 23.7, 19.8. HRMS (ESI-Orbitrap, negative mode) *m/z:* $[M-H]^-$ Calcd for $[C_{15}H_{21}O_2]^-$ 233.1536; Found 233.1545. Compound was isolated as a white crystalline powder.

3. Characterization data on artemisinin related products:

Artemisinic Alcohol (3)[3]

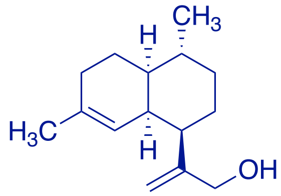

3
artemisinic alcohol

General procedure A was followed for this substrate. The crude product was purified by flash silica gel chromatography (10-30% EtOAc/hexanes) to afford the artemisinic alcohol in 81% yield (178 mg) isolated as a clear oil. NMR data matched what has been previously reported.

^{1}H NMR (500 MHz, $CDCl_3$) δ 5.22 (p, *J* = 1.4 Hz, 1H), 5.08 (h, *J* = 1.5 Hz, 1H), 4.87 (p, *J* = 1.4 Hz, 1H), 4.21 – 4.09 (m, 2H), 2.56 – 2.44 (m, 1H), 2.24 (d, *J* = 12.2 Hz, 1H), 2.00 – 1.86 (m, 2H), 1.83 – 1.76 (m, 1H), 1.72 (dq, *J* = 12.9, 3.5 Hz, 1H), 1.62 (s, 3H), 1.61 – 1.31 (m, 6H), 1.03 (dtd, *J* = 13.0, 11.6, 3.5 Hz, 1H), 0.91 (d, *J* = 6.4 Hz, 3H). ^{13}C NMR (126 MHz, $CDCl_3$) δ 151.1, 135.1, 120.5, 109.9, 65.7, 43.5, 41.7, 37.8, 35.3, 27.8, 26.4, 25.8, 25.7, 23.7, 19.8. HRMS (ESI-Orbitrap, positive mode) *m/z:* $[M+H]^+$ Calcd for $[C_{15}H_{25}O]^+$ 221.1900; Found: 221.1896.

Artemisinic Aldehyde (4)[3]

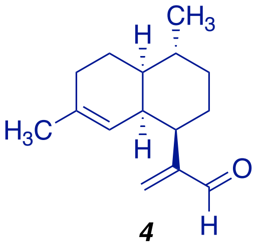

4
artemisinic aldehyde

To a round bottom flask, CuBr (36.6 mg, 10 mol %), 2,2'-bipyridine (39.0 mg, 10 mol %), TEMPO (2.9 mg, 0.75 mol %), and DMAP (4.6 mg, 0.75 mol%) were added along with a magnetic stir bar. The flask was then charged with acetonitrile (12 mL), evacuated, and backfilled with oxygen for three cycles and ultimately placed under an oxygen balloon to maintain an oxygen atmosphere. The alcohol **3** (551.0 mg, 2.5 mmol) was added via syringe, and the reaction was stirred under oxygen atmosphere until complete conversion to artemisinic aldehyde **4** was observed by HPLC assay (~1 hour). The subsequent material was purified by column chromatography to afford a 69% yield (374.4 mg). The compound was isolated as a clear crystalline solid.

^{1}H NMR (500 MHz, $CDCl_3$) δ 9.55 (s, 1H), 6.20 (s, 1H), 6.15 (s, 1H), 4.91 (s, 1H), 2.73 (dd, J = 12.3, 3.9 Hz, 1H), 2.55 (s, 1H), 1.98 – 1.85 (m, 2H), 1.82 – 1.70 (m, 2H), 1.60 (s, 3H), 1.59 – 1.53 (m, 1H), 1.52 – 1.35 (m, 4H), 1.10 (qd, J = 12.2, 3.9 Hz, 1H), 0.92 (d, J = 6.1 Hz, 3H). ^{13}C NMR (126 MHz, $CDCl_3$) δ 194.7, 152.5, 135.2, 134.7, 120.1, 41.3, 39.6, 37.4, 35.2, 27.6, 26.4, 25.5, 25.4, 23.7, 19.8. HRMS (ESI-Orbitrap, positive mode) *m/z:* $[M+H]^+$ Calcd for $[C_{15}H_{23}O]^+$ 219.1743; Found: 219.1744.

4. Site-selective functionalization of terpene-based natural products.

(+)-limonen-10-ol **(7)**[4]

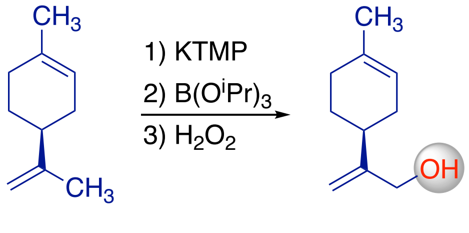

(+)-limonene *(89% purity)* from natural orange oil

7
(+)-limonen-10-ol

General procedure A was followed for this substrate using 136 mg of orange oil (89 wt% limonene, 121 mg, 0.89 mmol). The crude product was purified by flash silica gel chromatography (10-30% EtOAc/hexanes) to afford (+)-limonen-10-ol in 77% yield (104 mg). NMR data matched what has been reported previously. Compound isolated as a yellow oil.

1H NMR (500 MHz, CDCl3) δ 5.45 – 5.35 (m, 1H), 5.09 – 5.01 (m, 1H), 4.97 – 4.86 (m, 1H), 4.14 (s, 2H), 2.23 – 2.11 (m, 2H), 2.11 – 2.02 (m, 1H), 2.01 – 1.88 (m, 2H), 1.87 – 1.77 (m, 2H), 1.66 (s, 3H), 1.52 (dtd, J = 12.8, 11.3, 5.6 Hz, 1H). ^{13}C NMR (75 MHz, $CDCl_3$) δ 153.7, 133.8, 120.4, 107.9, 65.2, 36.9, 31.4, 30.5, 28.2, 23.5.

***Cuminyl alcohol* (8)**

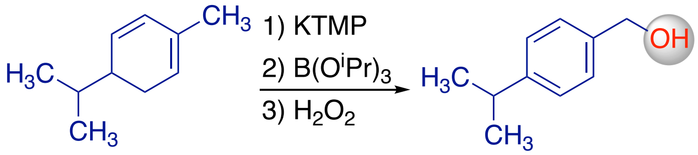

(±)-α-phellandrene

8
cuminyl alcohol

General procedure A was followed for this substrate. The crude product was purified by flash silica gel chromatography (10-30% EtOAc/hexanes) to afford the aromatized cuminyl alcohol in 48% yield (73 mg). NMR data matched an authentic commercial sample. Compound isolated as a clear oil.

^{1}H NMR (500 MHz, $CDCl_3$) δ 7.33 (d, J = 7.8 Hz, 2H), 7.26 (d, J = 7.8 Hz, 2H), 4.69 (d, J = 5.3 Hz, 2H), 2.94 (hept, J = 7.0 Hz, 1H), 1.64 – 1.55 (m, 1H), 1.28 (d, J = 6.9 Hz, 6H).^{13}C NMR (126 MHz, $CDCl_3$) δ 148.5, 138.3, 127.2, 126.7, 65.3, 33.9, 24.0 (2 CH_3's).

(-)-10-hydroxyperillyl alcohol (9)[5]

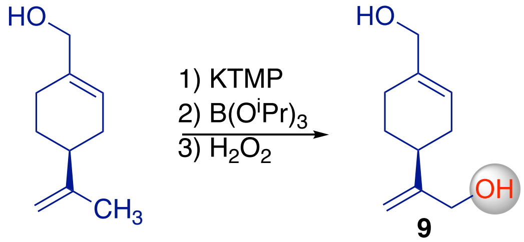

9

(-)-perillyl alcohol ***(-)-10-hydroxyperillyl alcohol***

General procedure A was followed using 2.5 equiv. each of *n*BuLi, KO*t*Bu, TMP, and B(O^{i}Pr)$_3$ and increasing to 5 equiv. of H_2O_2. The crude product was purified by flash silica gel chromatography (10-30% EtOAc/hexanes) to afford (-)-10-hydroxyperillyl alcohol in 70% average yield (**experiment #1**: 74%, 125 mg, **experiment #2**: 65%, 109 mg). NMR data matched what has been reported previously. Compound isolated as an off yellow oil.

^{1}H NMR (500 MHz, $CDCl_3$) δ 5.76 – 5.67 (m, 1H), 5.13 – 5.04 (m, 1H), 4.97 – 4.90 (m, 1H), 4.17 (s, 2H), 4.07 – 3.96 (m, 2H), 2.31 – 2.20 (m, 2H), 2.18 – 2.09 (m, 2H), 2.05 – 1.96 (m, 1H), 1.96 – 1.89 (m, 1H), 1.70 (s, 1H), 1.61 – 1.50 (m, 2H). ^{13}C NMR (126 MHz, $CDCl_3$) δ 153.3, 137.3, 122.2, 108.2, 67.2, 65.2, 36.9, 30.9, 27.8, 26.1. HRMS (ESI-Orbitrap, positive mode) *m/z:* $[M+Na^+]^+$ Calcd for $[C_{10}H_{16}O_2Na]^+$ 191.1043; Found: 191.1038.

***(+)-(Z)-8-hydroxycitronellene* (10)**

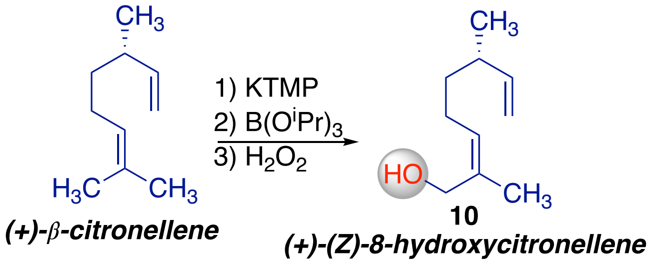

General procedure A was followed for this substrate. The crude product was purified by flash silica gel chromatography (0 to 20% EtOAc/hexanes) to afford (+)-(*Z*)-8-hydroxycitronellene in 61% yield (94 mg) and 86:14 *Z:E* stereoselectivity. Compound isolated as a clear oil.

^{1}H NMR (500 MHz, $CDCl_3$) δ 5.70 (ddd, *J* = 17.7, 10.2, 7.7 Hz, 1H), 5.30 (t, *J* = 7.4 Hz,1H), 5.01 – 4.92 (m, 2H), 4.14 (s, 2H), 2.14 (p, *J* = 6.9 Hz, 1H), 2.09 – 2.01 (m, 2H), 1.84 – 1.77 (m, 3H), 1.39 – 1.32 (m, 2H), 1.17 (s, 1H), 1.01 (d, *J* = 6.6 Hz, 3H). ^{13}C NMR (126 MHz, $CDCl_3$) δ 144.5, 134.2, 128.6, 112.8, 61.7, 37.4, 36.8, 25.3, 21.3, 20.3. HRMS (ESI-Orbitrap, positive mode) *m/z:* $[M+H]^+$ Calcd for $[C_{10}H_{19}O]^+$ 155.1430; Found: 155.1428.

***(±)-(Z)-8-hydroxycitronellol* (11)[6]**

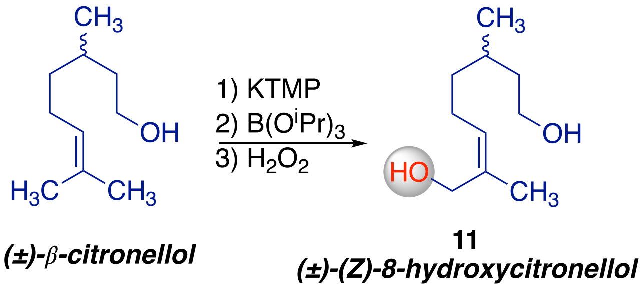

General procedure A was followed using 3 equiv. each of *n*BuLi, KO*t*Bu, TMP, and $B(O^iPr)_3$ and increasing to 6 equiv. of H_2O_2. The crude product was purified by flash silica gel chromatography (17-80% EtOAc/hexanes) to afford (±)-(*Z*)-8-hydroxycitronellol in 91% yield (84:16 Z:E 156 mg). NMR data matched what has been reported previously. Compound isolated as a clear oil.

^{1}H NMR (500 MHz, $CDCl_3$) δ 5.29 (t, *J* = 7.4 Hz, 1H), 4.22 (d, *J* = 11.7 Hz, 1H), 4.08 (d, *J* = 11.8 Hz, 1H), 3.78 – 3.63 (m, 2H), 2.18 (dq, *J* = 15.4, 7.8 Hz, 1H), 2.04 (dq, 1H), 1.72 – 1.56 (m, 3H), 1.55 – 1.44 (m, 1H), 1.41 – 1.31 (m, 2H), 1.26 (ddt, *J* = 13.5, 8.6, 6.8 Hz, 1H), 0.93 (d, *J* = 6.6 Hz, 3H). ^{13}C NMR (126 MHz, $CDCl_3$) δ 134.4, 128.6, 61.5, 60.9, 39.2, 37.1, 28.5, 24.9, 21.3, 19.7.

***(-)-10-hydroxyisopulegol* (12)**[7, 8]

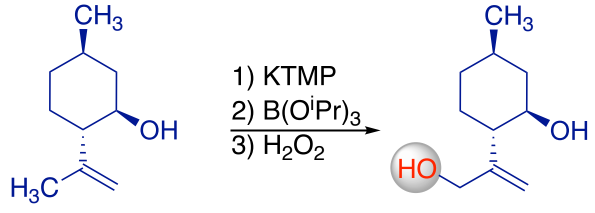

(-)-isopulegol

12
(-)-10-hydroxyisopulegol

General procedure A was followed using 2.5 equiv. each of *n*BuLi, KO*t*Bu, TMP, and B(O^{i}Pr)$_3$ and increasing to 5 equiv. of H_2O_2. The crude product was purified by flash silica gel chromatography (20-100% EtOAc/hexanes) to afford (-)-10-hydroxyisopulegol in 93% yield (159 mg). NMR data matched what has been reported previously. Compound isolated as a white powder.

^{1}H NMR (500 MHz, $CDCl_3$) δ 5.29 – 5.17 (m, 1H), 5.07 (s, 1H), 4.14 (q, *J* = 12.9 Hz, 2H), 3.57 (td, *J* = 10.5, 4.2 Hz, 1H), 2.37 – 2.14 (m, 2H), 2.04 (dtd, *J* = 12.3, 3.9, 1.9 Hz, 1H), 1.97 (ddd, *J* = 13.1, 10.0, 3.6 Hz, 1H), 1.80 – 1.65 (m, 2H), 1.60 – 1.49 (m, 1H), 1.37 (qd, *J* = 12.9, 3.4 Hz, 1H), 1.04 (q, 1H), 1.00 – 0.89 (m, 4H). ^{13}C NMR (126 MHz, $CDCl_3$) δ 150.8, 112.5, 73.2, 65.8, 50.1, 43.5, 34.5, 31.5, 31.3, 22.2. HRMS (ESI-Orbitrap, positive mode) *m/z:* [M+Na$^+$]$^+$ Calcd for $[C_{10}H_{18}O_2Na]^+$ 193.1199; Found: 193.1194.

***Valencene-13-ol* (13)**[9]

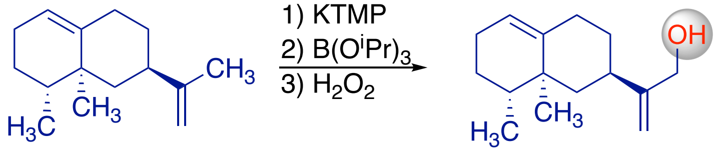

(+)-valencene
natural grade purity (74%)

13
(+)-valencen-13-ol

General procedure A was followed using 204 mg of natural grade valencene (74 wt% valencene, 151 mg, 0.74 mmol). The crude product was purified by flash silica gel chromatography (0 to 20% EtOAc/hexanes) to afford (+)-valencen-13-ol as a clear oil in 77% yield (126 mg). NMR data matched what has been reported previously.

^{1}H NMR (500 MHz, $CDCl_3$) δ 5.39 – 5.31 (m, 1H), 5.06 – 5.02 (m, 1H), 4.92 – 4.88 (m, 1H), 4.15 (m, 2H), 2.33 (tq, *J* = 11.9, 2.7 Hz, 2H), 2.12 (ddd, *J* = 14.1, 4.1, 2.5 Hz, 1H), 2.08 – 2.01 (m, 1H), 2.00 – 1.88 (m, 2H), 1.84 (ddq, *J* = 12.8, 5.1, 2.7 Hz, 1H), 1.66 – 1.53 (m, 1H), 1.49 – 1.40 (m, 3H), 1.35 (t, *J* = 6.2 Hz, 1H), 1.24 (dtd, *J* = 13.8, 12.3, 4.1 Hz, 1H), 1.02 (t, *J* = 12.8 Hz, 1H), 0.98 (s, 2H), 0.91 – 0.88 (m, 2H). ^{13}C NMR (126 MHz, $CDCl_3$) δ 154.1, 142.8, 120.4, 107.9, 65.3, 45.5, 40.9, 38.0, 36.7, 33.6, 32.7, 27.1, 25.9, 18.4, 15.7.

10-hydroxycannabidiol **(14)**[10]

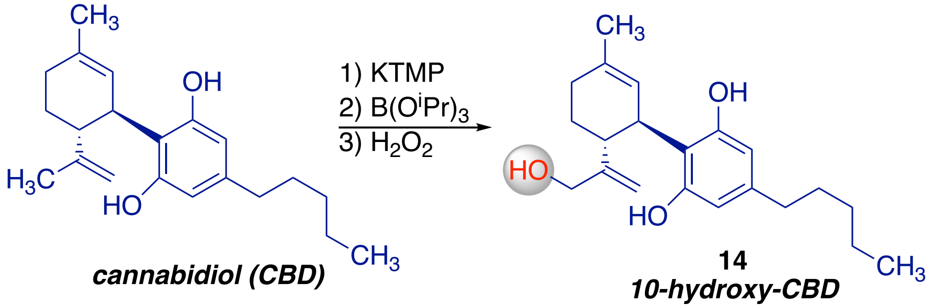

General procedure A was followed using 4 equiv. each of *n*BuLi, KO*ᵗ*Bu, TMP, and B(O*ⁱ*Pr)$_3$ and increasing to 8 equiv. of H_2O_2. The crude product was purified by flash silica gel chromatography (10-100% EtOAc/hexanes) to afford 10-hydroxy-cannabidiol in 52% yield (172 mg) isolated as a clear oil. ^{1}H NMR data matched what has been reported previously but ^{13}C NMR has not been published to the best of our knowledge.

^{1}H NMR (500 MHz, $CDCl_3$) δ 6.71 (s, 1H), 6.31 (s, 1H), 6.23 (s, 1H) 5.96 (s, 1H), 5.65 – 5.55 (m, 1H), 4.97 – 4.89 (m, 1H), 4.69 – 4.59 (m, 1H), 4.31 (d, *J* = 12.2 Hz, 1H), 2.69 (s, 1H), 2.54 (td, *J* = 11.3, 3.3 Hz, 1H), 2.50 – 2.40 (m, 2H), 2.34 – 2.22 (m, 1H), 2.18 – 2.11 (m, 1H), 2.05 – 1.86 (m, 2H), 1.82 (s, 3H), 1.69 (s, 1H), 1.58 (p, *J* = 7.5 Hz, 2H), 1.38 – 1.25 (m, 4H), 0.90 (t, *J* = 6.9 Hz, 3H). ^{13}C NMR (126 MHz, $CDCl_3$) δ 156.0, 154.0, 148.6, 143.3, 140.1, 124.0, 115.6, 113.3, 109.6, 108.7, 65.0, 44.5, 38.1, 35.5, 31.5, 30.7, 30.3, 27.4, 23.7, 22.6, 14.1. HRMS (ESI-Orbitrap, positive mode) *m/z:* $[M+H^+]^+$ Calcd for $[C_{21}H_{31}O_3]^+$ 331.2268; Found: 331.2262

5. Modified synthesis of amorphadiene

Artemisinic alcohol (3) from artemisinic acid (5).

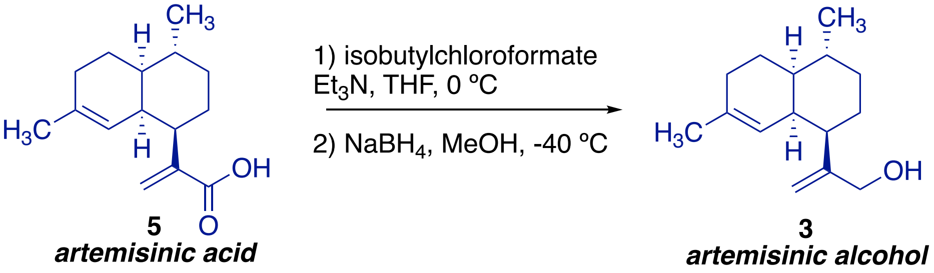

Isobutyl chloroformate (3.47 g, 3.29 mL, 25.38 mmol) was added to a chilled (0 °C) solution of Artemisinic acid **5** (5.0 g, 21.15 mmol) in THF (90 mL). After stirring 5 min, TEA (3.21 g, 4.42 mL, 31.73 mmol) was added dropwise and the mixture stirred for 1 h, before filtering through Celite. The mixed anhydride solution was added in one portion to pre-cooled MeOH (90 mL) at -40 °C, and the mixture was stirred for 5 min before adding $NaBH_4$ (3.2 g, 84.6 mmol) in one portion. The reaction mixture was then allowed to warm to room temperature and monitored for reaction completion (TLC: 10% EtOAc in hexanes + 2% TEA), before quenching with aqueous NH_4Cl (5% (w/w), 150 mL), and the mixture was then extracted with EtOAc (3 x 150 mL). The organic layers were separated, dried over Na_2SO_4, and concentrated under reduced pressure to

recover 6.7 g crude **2**, which was subsequently purified by chromatography (column: 40 g silica gel; eluent: gradient EtOAc 0 →25% in hexane) to isolate 3.81 g (81%) of pure **3** as a clear oil. Characterization conforms to previously reported data.[11]

2-[(1R,4R,4aS,8aR)-4,7-dimethyl-1,2,3,4,4a,5,6,8a-octahydronaphthalen-1-yl]prop-2-en-1-yl acetate (S1)[11]

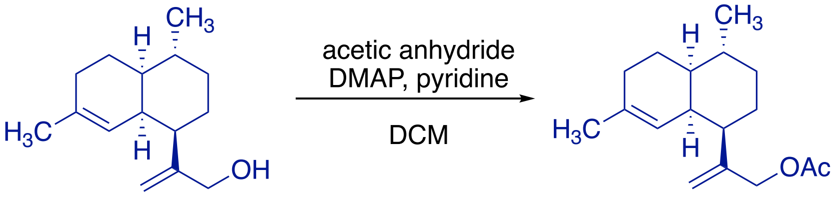

3 *artemisinic alcohol* **S1**

Acetic anhydride (15.01 g, 13.9 mL, 147.03 mmol) was added to a solution of pyridine (12.92 g, 13.21 mL, 163.35 mmol) and **2** (7.2 g, 32.67 mmol) in CH_2Cl_2 (80 mL) at room temperature. The mixture was stirred until determined to be complete by TLC (10% EtOAc in hexanes, ca ~16 h), whereupon the reaction was diluted with CH_2Cl_2 (200 mL) and washed sequentially with aqueous citric acid (5% (w/w), 2 x 150 mL), NH_4OH (10% (w/w), 2 x 150 mL), H_2O (1 x 150 mL) and brine (1 x 150 mL). The organic layers were dried over Na_2SO_4 and concentrated under reduced pressure to recover 9.35 g crude **S1** which was subsequently purified by chromatography (column: 40 g silica gel; eluent: gradient EtOAc 0 →15% in hexane) to isolate 7.43 g (87%) of pure **S1** as a clear oil. Characterization conforms to previously reported data. ^{1}H NMR (500 MHz, $CDCl_3$) δ 5.22 (s, 1H), 5.06 (s, 1H), 4.93 (s, 1H), 4.62 (d, J = 13.3 Hz, 1H), 4.54 (d, J = 13.3 Hz, 1H), 2.51 (s, 1H), 2.20 (d, J = 12.5 Hz, 1H), 2.12 (s, 3H), 2.01 – 1.94 (m, 1H), 1.94 – 1.85 (m, 1H), 1.80 (dd, J = 17.3, 6.0 Hz, 1H), 1.72 (dq, J = 12.6, 3.4 Hz, 1H), 1.62 (s, 3H), 1.60 – 1.49 (m, 2H), 1.48 – 1.26 (m, 3H), 1.03 (qd, J = 12.9, 3.2 Hz, 1H), 0.91 (d, J = 6.5 Hz, 3H). ^{13}C NMR (126 MHz, $CDCl_3$) δ 170.9, 146.0, 135.1, 120.2, 112.5, 66.5, 43.7, 41.6, 37.7, 35.3, 27.8, 26.4, 25.70, 25.67, 23.7, 21.0, 19.8.

Amorpha-4,11-diene (2).[12]

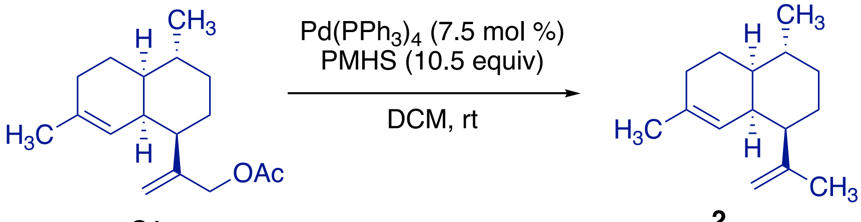

S1 **2** *amorphadiene*

A solution of **S1** (1.95 g, 7.24 mmol) and $Pd(PPh_3)_4$ (0.42 g, 0.36 mmol) in CH_2Cl_2 (75 mL) was de-gassed by evacuating the headspace of the reaction vessel under vacuum and back-filling with nitrogen (3 x), before adding polymethylhydrosiloxane (PMHS) (3.05 g, 3.03 mL, 50.68 mmol) in one portion at room temperature. The reaction was stirred for 6 h, whereupon additional $Pd(PPh_3)_4$ (0.21 g, 0.18 mmol) and PMHS (1.51 g, 1.5 mL, 25.09 mmol) was added and the reaction continued to stir until determined complete by TLC (2% EtOAc in hexanes, ca. ~6-48

h). The reaction was concentrated to a residue under reduced pressure and dissolved in hexanes (80 mL) before adding aqueous NaOH (20% (w/w), 80 mL) and the mixture stirred for an additional 16 h. The organic layers were separated and washed with H_2O (2 x 100 mL), dried over Na_2SO_4, and concentrated under reduced pressure. The crude material was then purified by chromatography (column: 12 g silica gel; eluent: 100% hexane) to isolate 1.42 g (96%) of pure **2** as a clear oil. Characterization conforms to previously reported data.[12] ^{1}H NMR (500 MHz, $CDCl_3$) δ 5.08 (s, 1H), 4.89 (s, 1H), 4.67 (s, 1H), 2.58 (s, 1H), 2.06 – 1.85 (m, 3H), 1.81 (dd, *J* = 14.0, 4.1 Hz, 1H), 1.77 (s, 3H), 1.70 (dq, *J* = 12.7, 3.6 Hz, 1H), 1.63 (d, *J* = 2.5 Hz, 3H), 1.61 – 1.48 (m, 2H), 1.48 – 1.38 (m, 1H), 1.38 – 1.24 (m, 2H), 1.00 (qd, *J* = 12.5, 3.4 Hz, 1H), 0.91 (d, *J* = 6.4 Hz, 3H). ^{13}C NMR (126 MHz, $CDCl_3$) δ 148.0, 134.6, 120.9, 109.8, 47.6, 41.8, 37.6, 35.4, 27.9, 26.5, 26.1, 25.8, 23.7, 22.6, 19.8.

References

(1) Jakhar, V. K.; Johnson, E. C.; Kavuturu, A.; Heller, J. K.; Veige, A. S.; Ghiviriga, I. Precise NMR Method for Titering Organometal Reagents. *Organic Letters* **2021**, *23* (13), 4945-4948. DOI: 10.1021/acs.orglett.1c01006

(2) Xu, M.-M.; Yang, L.; Tan, K.; Chen, X.; Lu, Q.-T.; Houk, K. N.; Cai, Q. An enantioselective ambimodal cross-Diels–Alder reaction and applications in synthesis. *Nature Catalysis* **2021**, *4* (10), 892-900. DOI: 10.1038/s41929-021-00687-x

(3) Bertea, C. M.; Freije, J. R.; van der Woude, H.; Verstappen, F. W.; Perk, L.; Marquez, V.; De Kraker, J. W.; Posthumus, M. A.; Jansen, B. J.; de Groot, A.; Franssen, M. C.; Bouwmeester, H. J. Identification of intermediates and enzymes involved in the early steps of artemisinin biosynthesis in Artemisia annua. *Planta Med* **2005**, *71* (1), 40-47. DOI: 10.1055/s-2005-837749

(4) Serra, S.; Fuganti, C.; Gatti, F. G. A Chemoenzymatic, Preparative Synthesis of the Isomeric Forms ofp-Menth-1-en-9-ol: Application to the Synthesis of the Isomeric Forms of the Cooling Agent 1-Hydroxy-2,9-cineole. *European Journal of Organic Chemistry* **2008**, *2008* (6), 1031-1037. DOI: 10.1002/ejoc.200701010

(5) Suemune, H.; Iwasaki, G.; Ueno, K.; Sakai, K. The synthesis of 9-substituted p-mentha-1,8(10)-diene derivatives. *Chemical and Pharmaceutical Bulletin* **1984**, *32* (11), 4632-4636. DOI: 10.1248/cpb.32.4632

(6) Zhao, X. Z.; Tu, Y. Q.; Peng, L.; Li, X. Q.; Jia, Y. X. Synthetic studies of the HIV-1 protease inhibitive didemnaketals: stereocontrolled synthesis of an ester side chain. *Tetrahedron Letters* **2004**, *45* (19), 3713-3716. DOI: 10.1016/j.tetlet.2004.03.103

(7) Schlosser, M.; Kotthaus, M. Isopulegol as a Model Compound: Metalation and Substitution of an Allylic Position in the Presence of an Unprotected Hydroxy Function. *European Journal of Organic Chemistry* **1999**, *1999* (2), 459-462. DOI: 10.1002/(sici)1099-0690(199902)1999:2<459::Aid-ejoc459>3.0.Co;2-f

(8) Friedrich, D.; Bohlmann, F. Total synthesis of various elemanolides. *Tetrahedron* **1988**, *44* (5), 1369-1392. DOI: 10.1016/s0040-4020(01)85916-0

(9) Khasawneh, M. A.; Xiong, Y.; Peralta-Cruz, J.; Karchesy, J. J. Biologically important eremophilane sesquiterpenes from alaska cedar heartwood essential oil and their semi-synthetic derivatives. *Molecules* **2011**, *16* (6), 4775-4785. DOI: 10.3390/molecules16064775

(10) Jorapur, V. S.; Duffley, R. P.; Razdan, R. K. A Procedure for the Conversion of Cannabidiol into 12β-Substituted Tetrahydrocannabinols (THC's): Synthesis of 12β-Hydroxy-ΔβTHC1. *Synthetic Communications* **1984**, *14* (7), 655-660. DOI: 10.1080/00397918408063751

(11) Zanetti, A.; Schwertz, G.; De Oliveira, M. N.; Gomez Fernandez, M. A.; Amara, Z.; Cossy, J. Palladium-Catalyzed Regioselective Allylic Oxidation of Amorphadiene, a Precursor of

Artemisinin. *The Journal of Organic Chemistry* **2021**, *86* (11), 7603-7608. DOI: 10.1021/acs.joc.1c00653

(12) Schwertz, G.; Zanetti, A.; Nascimento De Oliveira, M.; Gomez Fernandez, M. A.; Dioury, F.; Cossy, J.; Amara, Z. Synthesis of amorpha-4,11-diene from dihydroartemisinic acid. *Tetrahedron* **2019**, *75* (6), 743-748. DOI: 10.1016/j.tet.2018.12.050

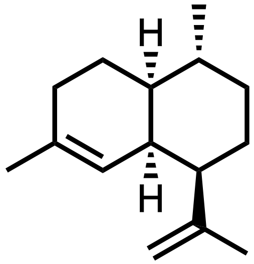

2

^{1}H NMR (500 MHz, $CDCl_3$)

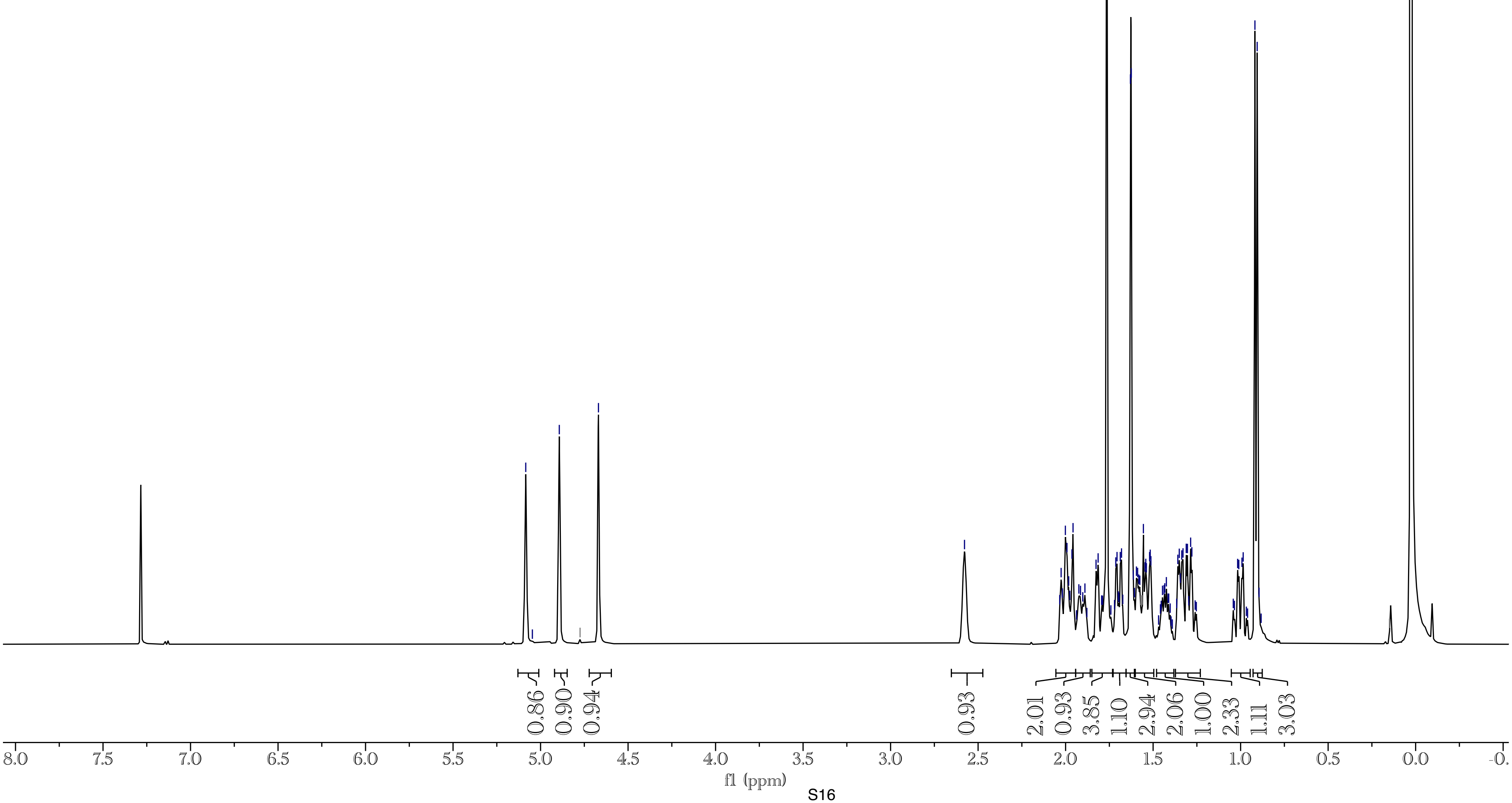

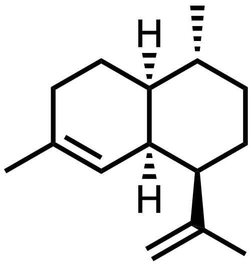

2

^{13}C NMR (126 MHz, $CDCl_3$)

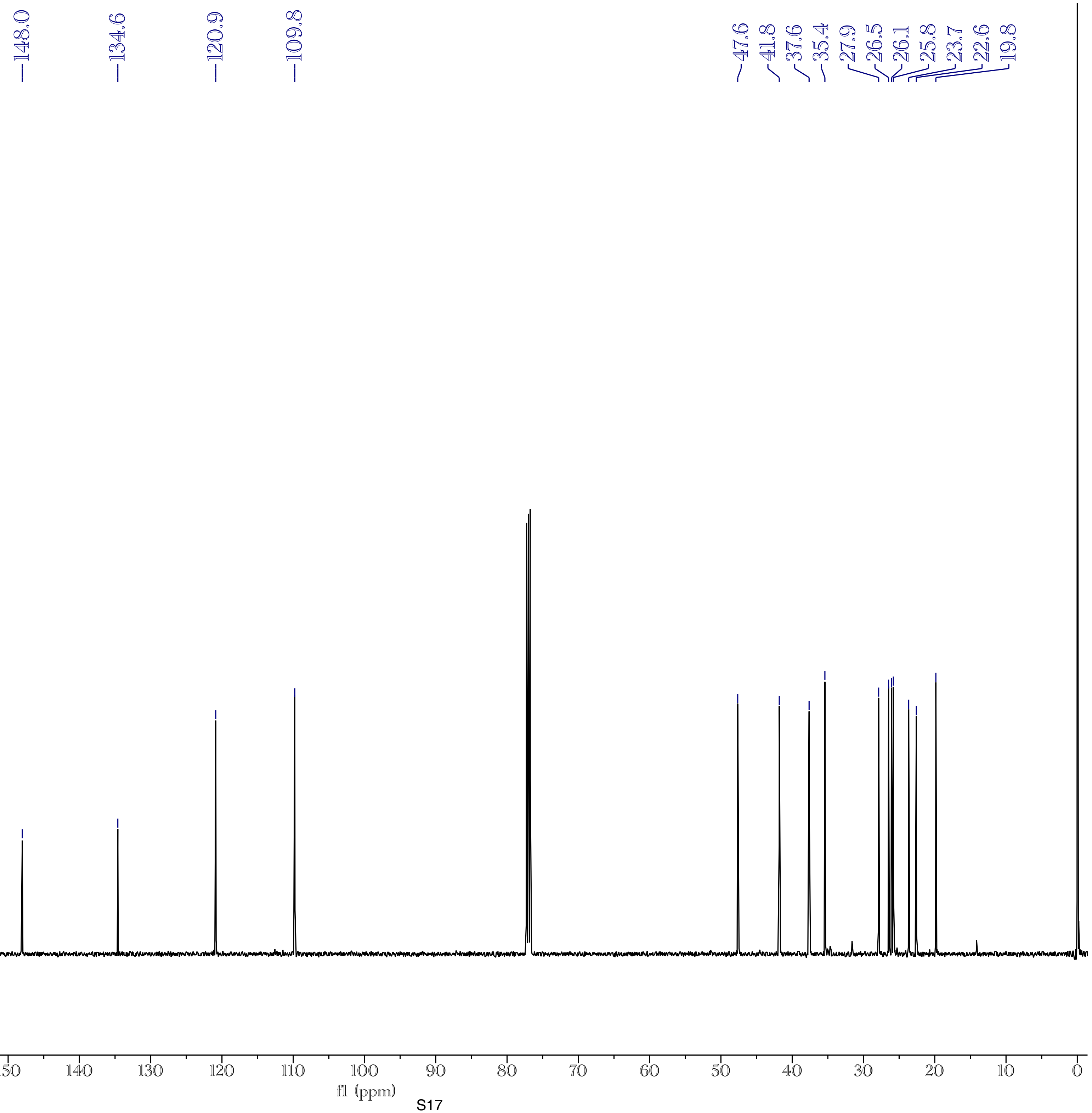

3

^{1}H NMR (500 MHz, $CDCl_3$)

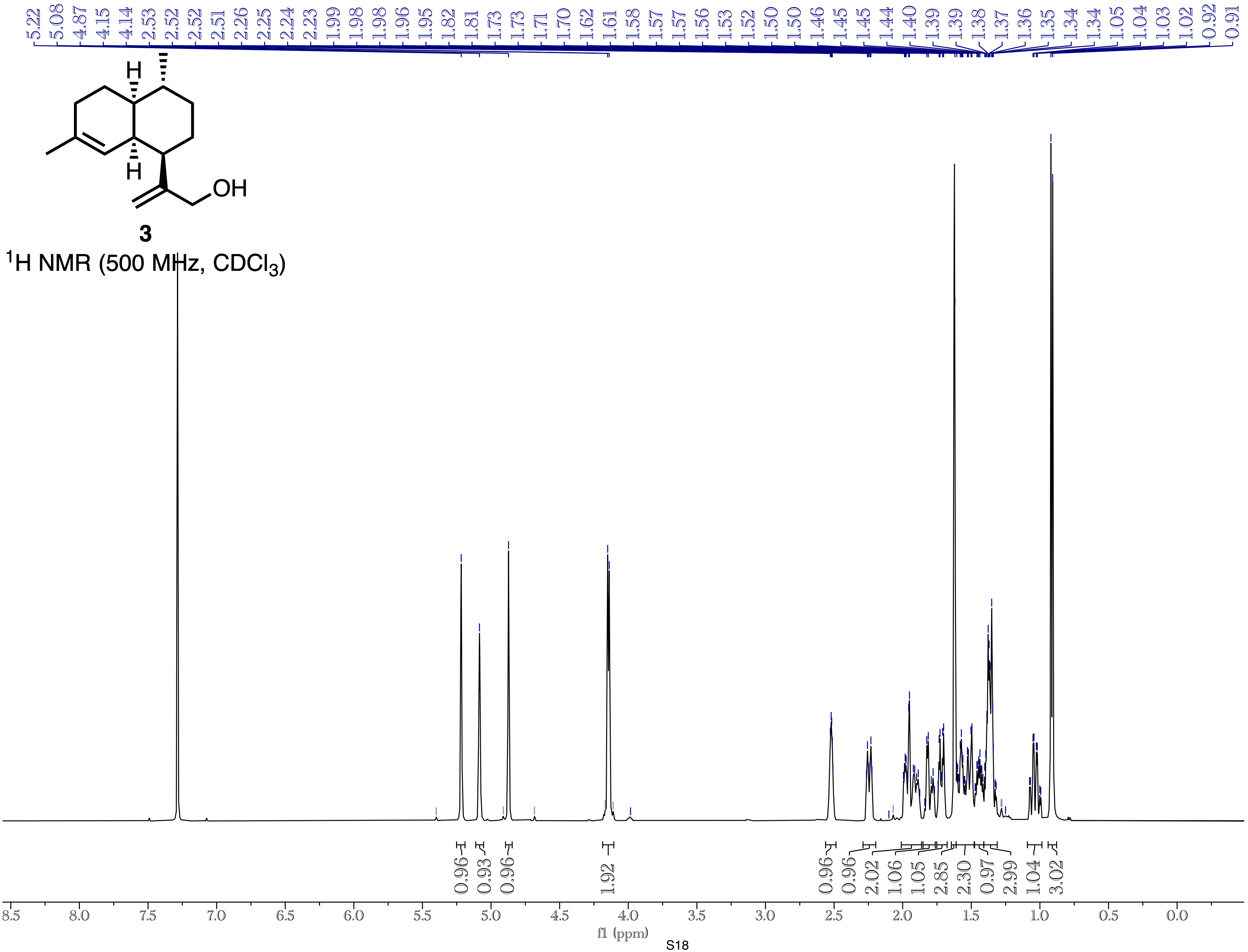

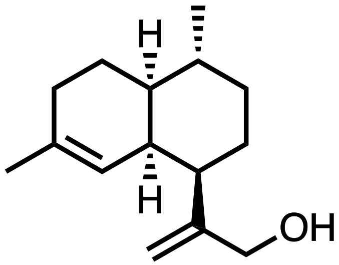

3

^{13}C NMR (126 MHz, $CDCl_3$)

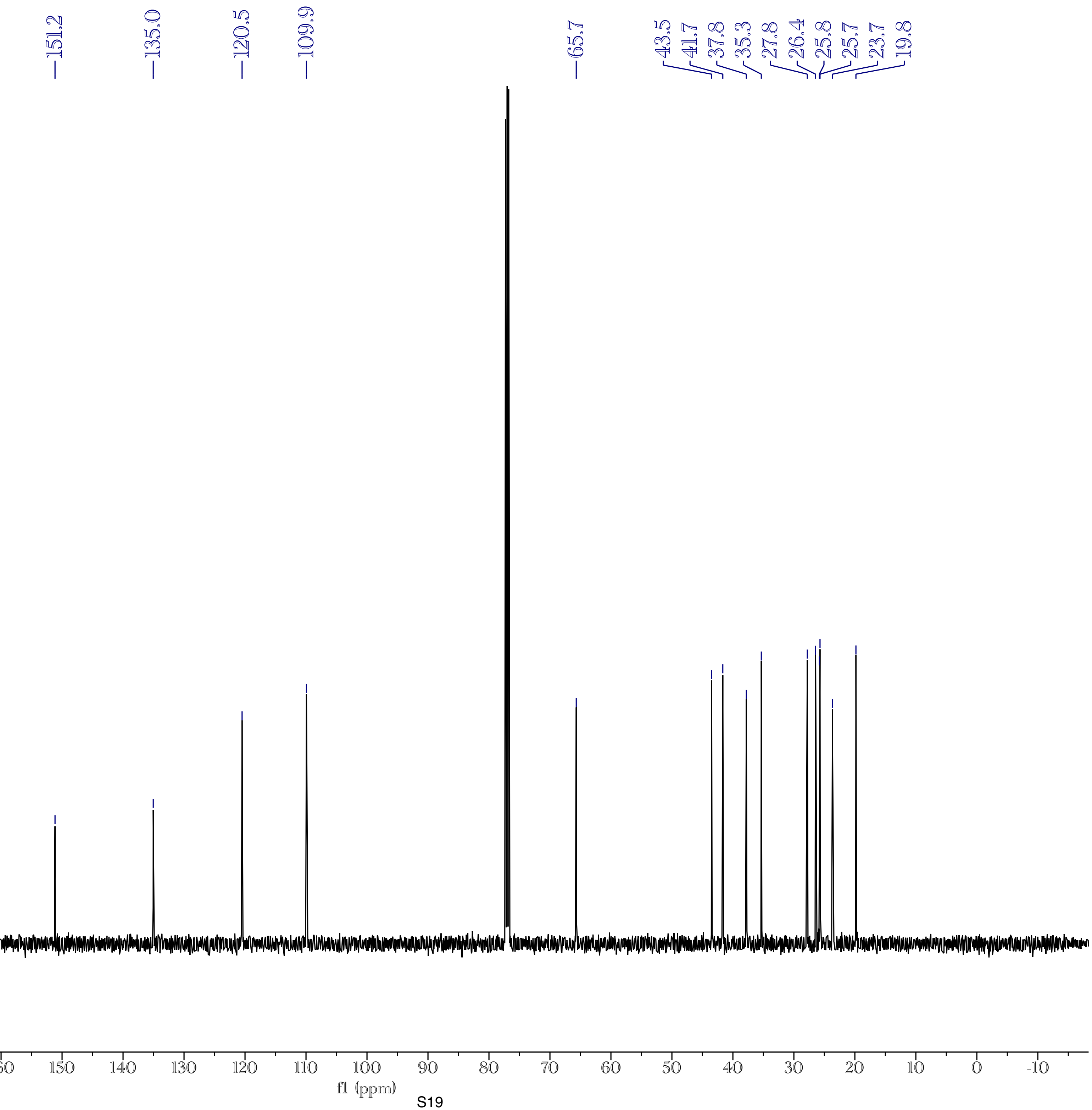

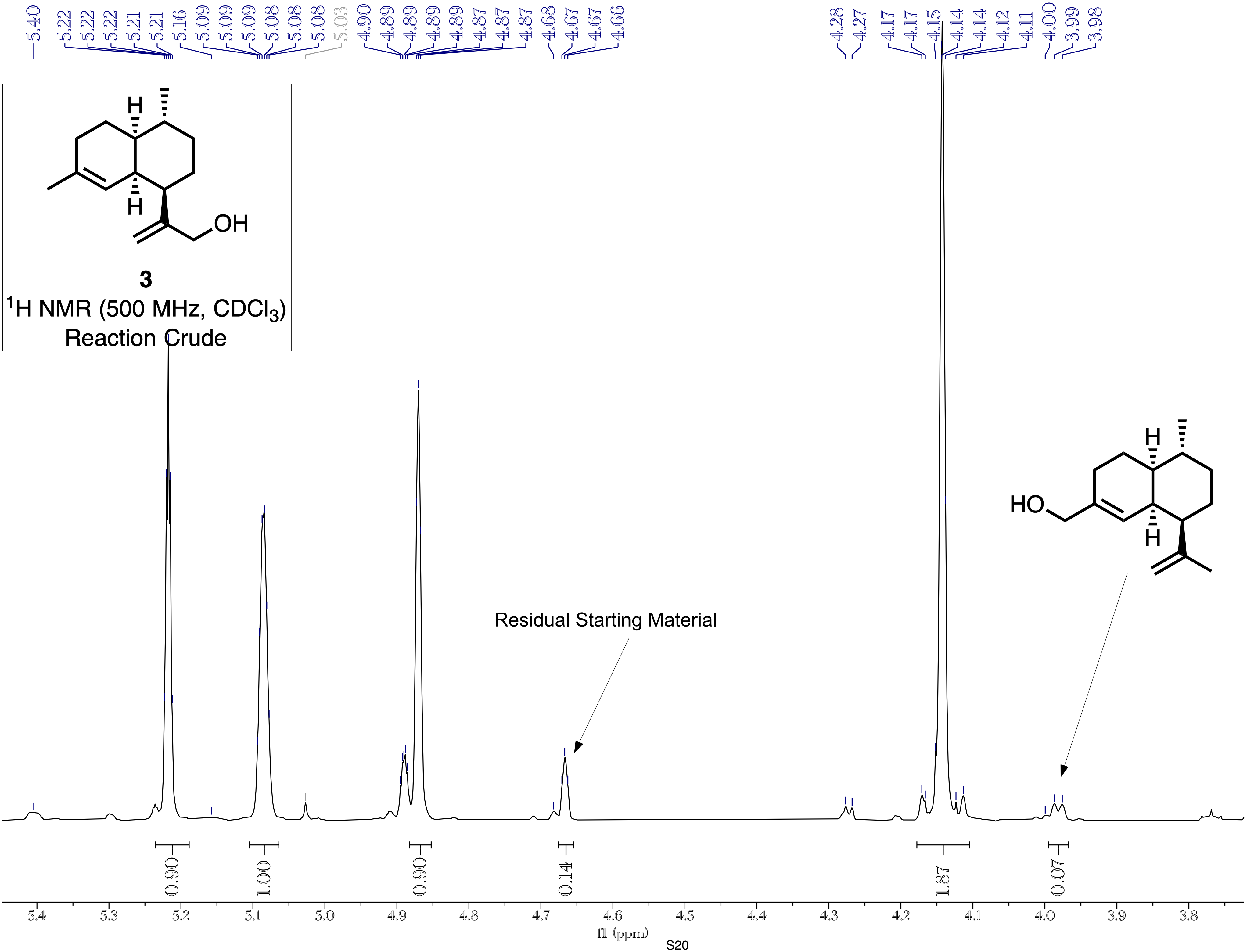
5.40
5.22
5.22
5.22
5.21
5.21
5.16
5.09
5.09
5.09
5.08
5.08
5.08
5.03
4.90
4.89
4.89
4.89
4.89
4.87
4.87
4.87
4.68
4.67
4.67
4.66
4.28
4.27
4.17
4.17
4.15
4.14
4.14
4.12
4.11
4.00
3.99
3.98
H
H
OH
3
^{1}H NMR (500 MHz, $CDCl_3$)
Reaction Crude
HO
H
H
Residual Starting Material
0.90
1.00
0.90
0.14
1.87
0.07
5.4
5.3
5.2
5.1
5.0
4.9
4.8
4.7
4.6
4.5
4.4
4.3
4.2
4.1
4.0
3.9
3.8
f1 (ppm)

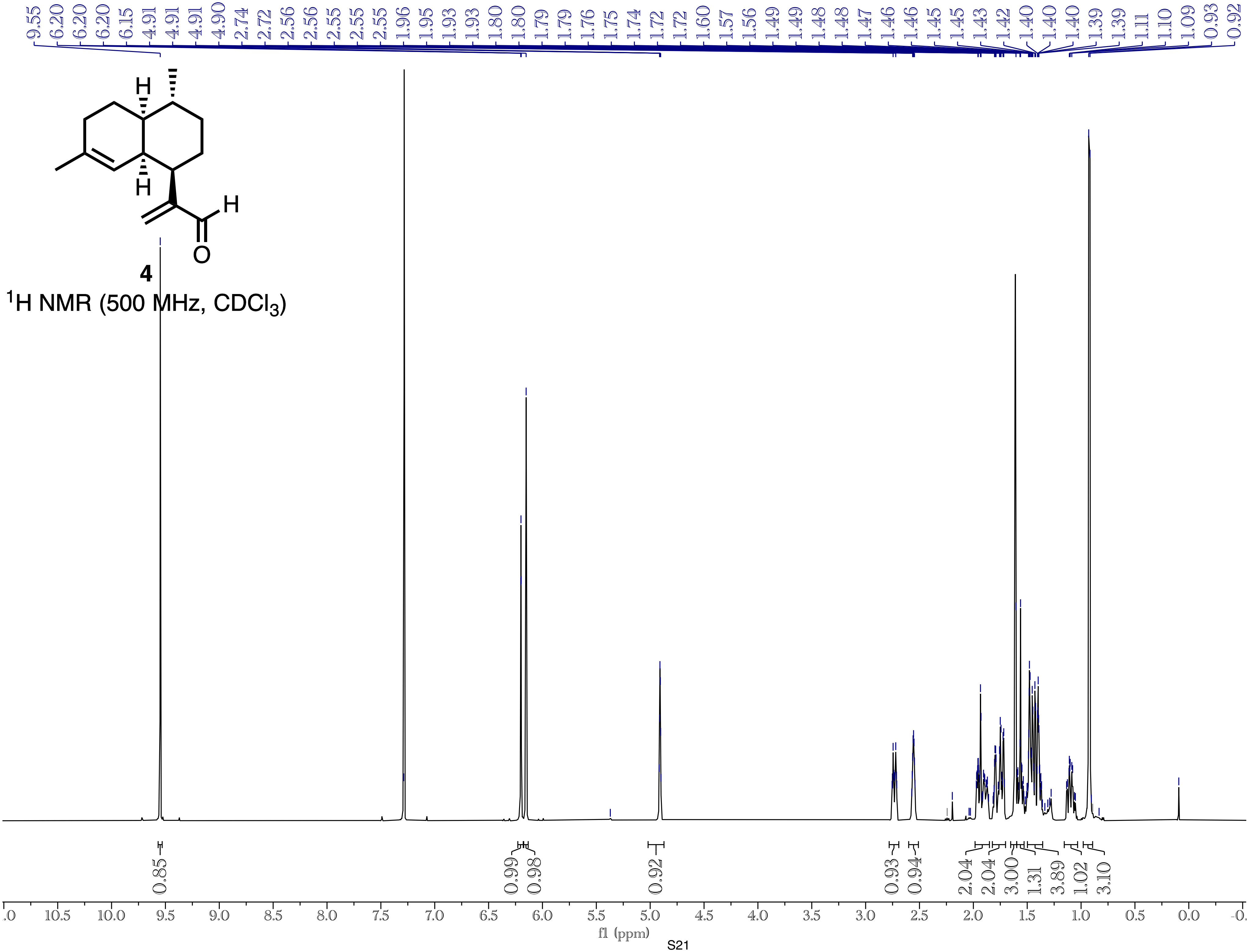

4

^{1}H NMR (500 MHz, $CDCl_3$)

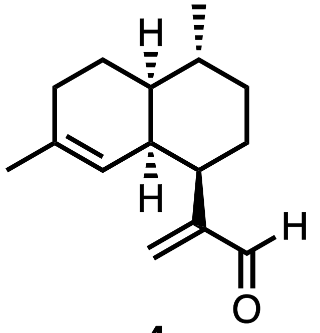

4

^{13}C NMR (126 MHz, $CDCl_3$)

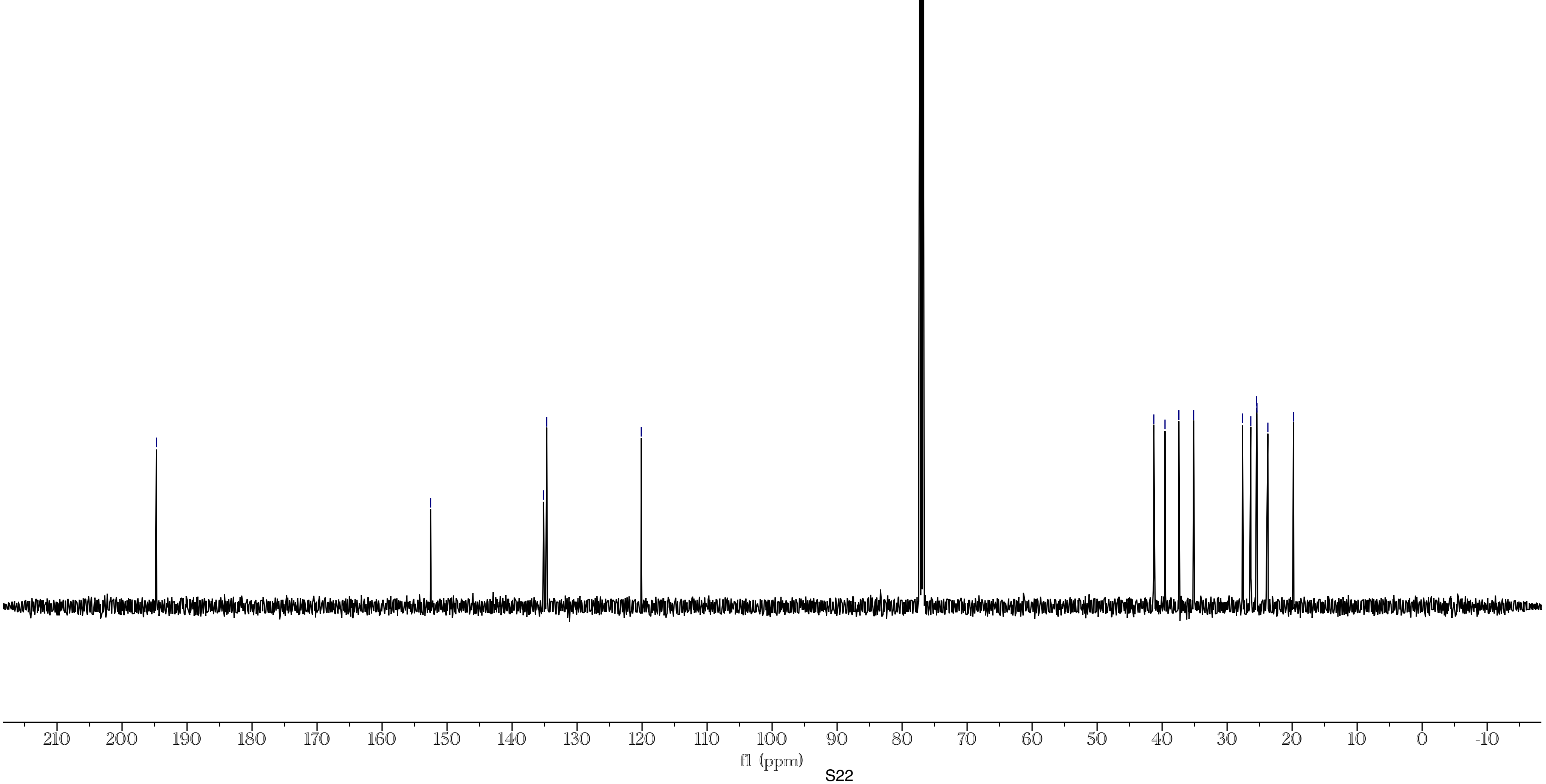

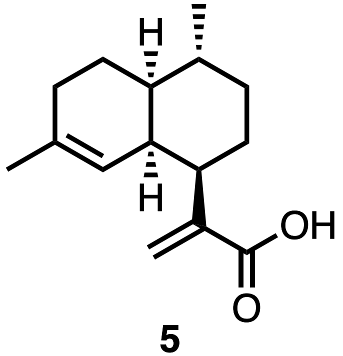

5

^{1}H NMR (500 MHz, $CDCl_3$)

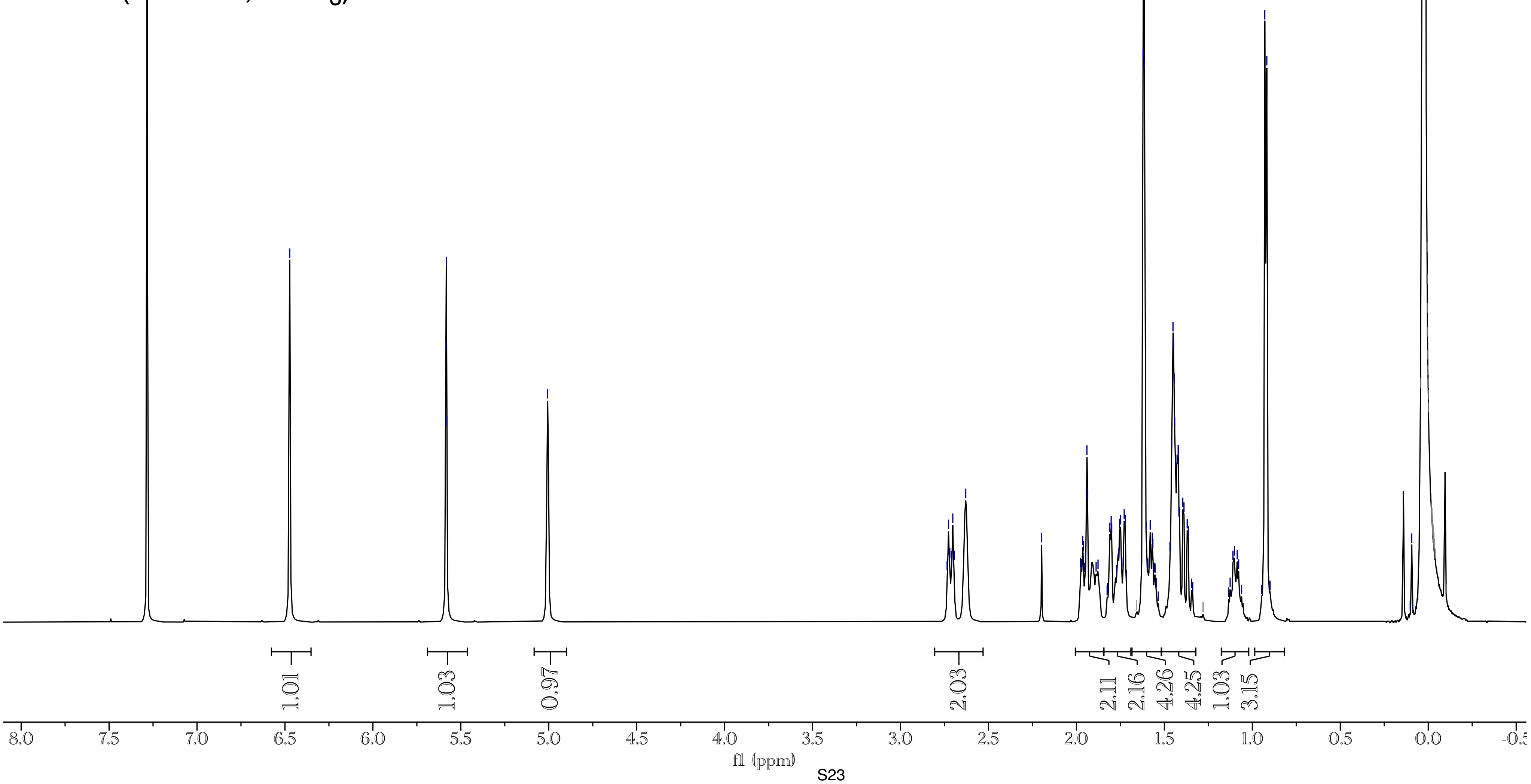

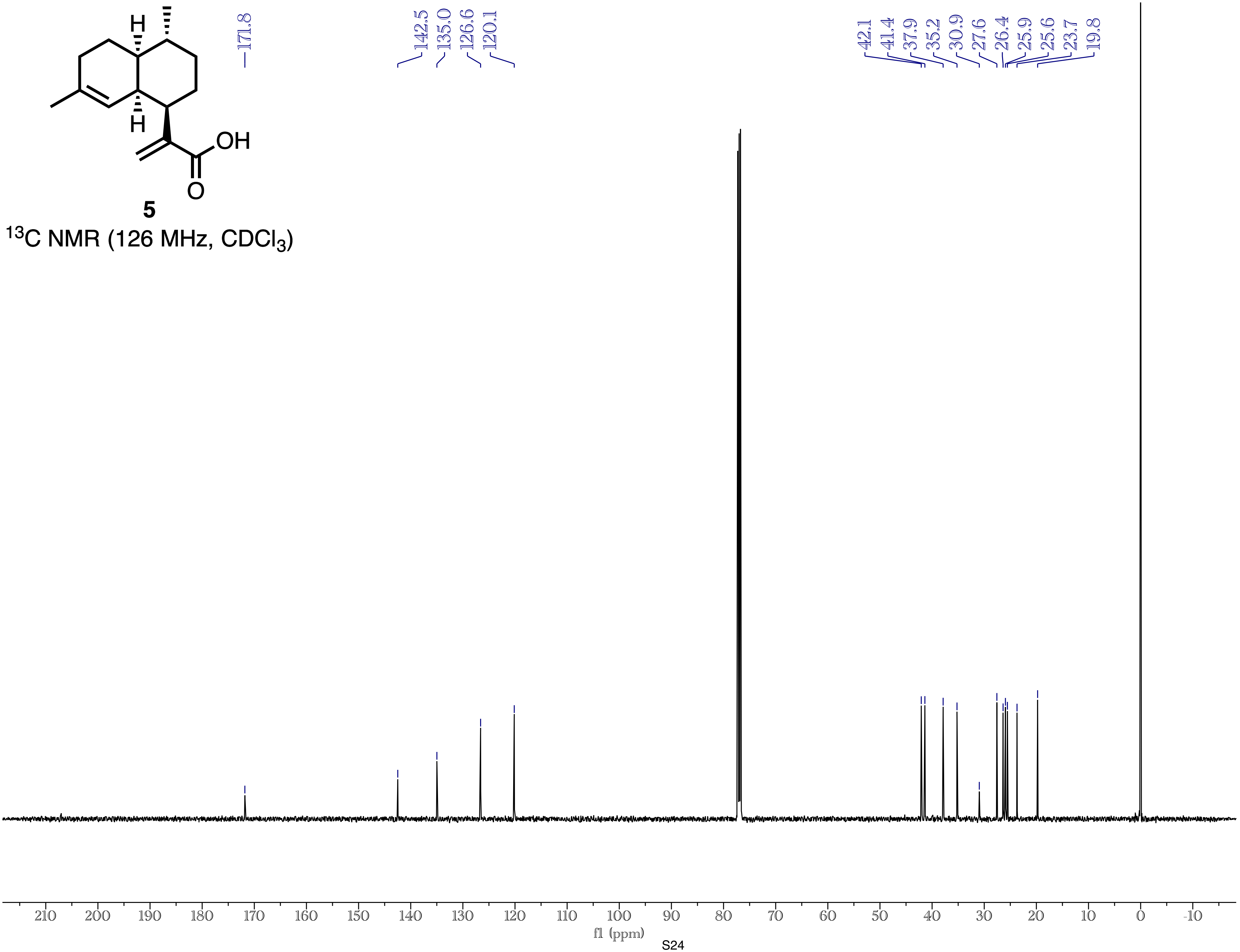
5
^{13}C NMR (126 MHz, $CDCl_3$)
171.8
142.5
135.0
126.6
120.1
42.1
41.4
37.9
35.2
30.9
27.6
26.4
25.9
25.6
23.7
19.8
f1 (ppm)

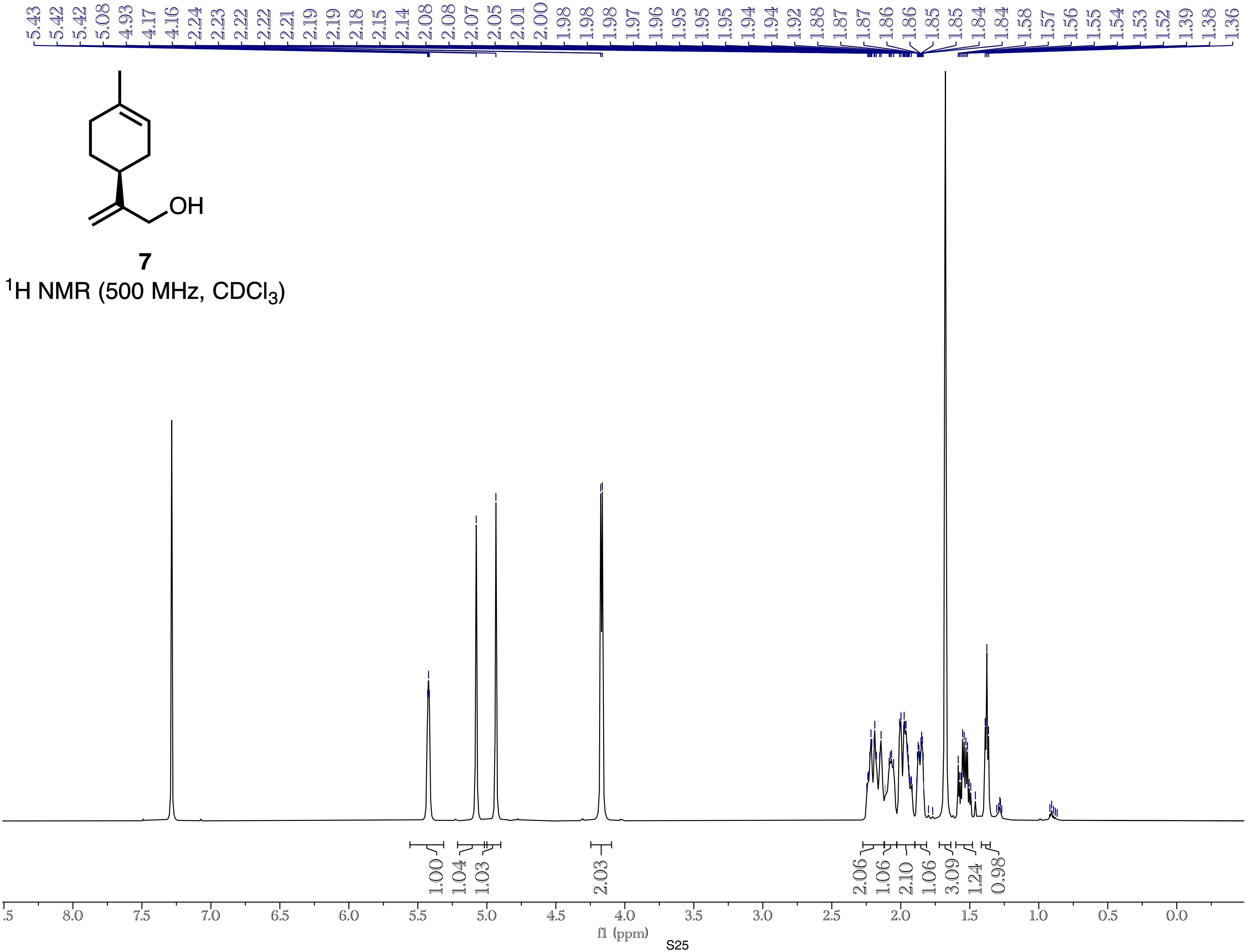
5.43 5.42 5.42 5.08 4.93 4.17 4.16 2.24 2.23 2.22 2.22 2.21 2.19 2.19 2.18 2.15 2.14 2.08 2.08 2.07 2.05 2.01 2.00 1.98 1.98 1.98 1.97 1.96 1.95 1.95 1.95 1.94 1.94 1.92 1.88 1.87 1.87 1.86 1.86 1.85 1.85 1.84 1.84 1.58 1.57 1.56 1.55 1.54 1.53 1.52 1.39 1.38 1.36
OH
7
^{1}H NMR (500 MHz, $CDCl_3$)
1.00 1.04 1.03 2.03 2.06 1.06 2.10 1.06 3.09 1.24 0.98
.5 8.0 7.5 7.0 6.5 6.0 5.5 5.0 4.5 4.0 3.5 3.0 2.5 2.0 1.5 1.0 0.5 0.0
f1 (ppm)

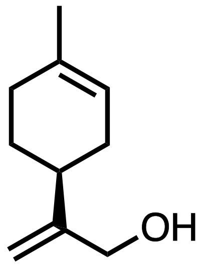

7

^{13}C NMR (126 MHz, $CDCl_3$)

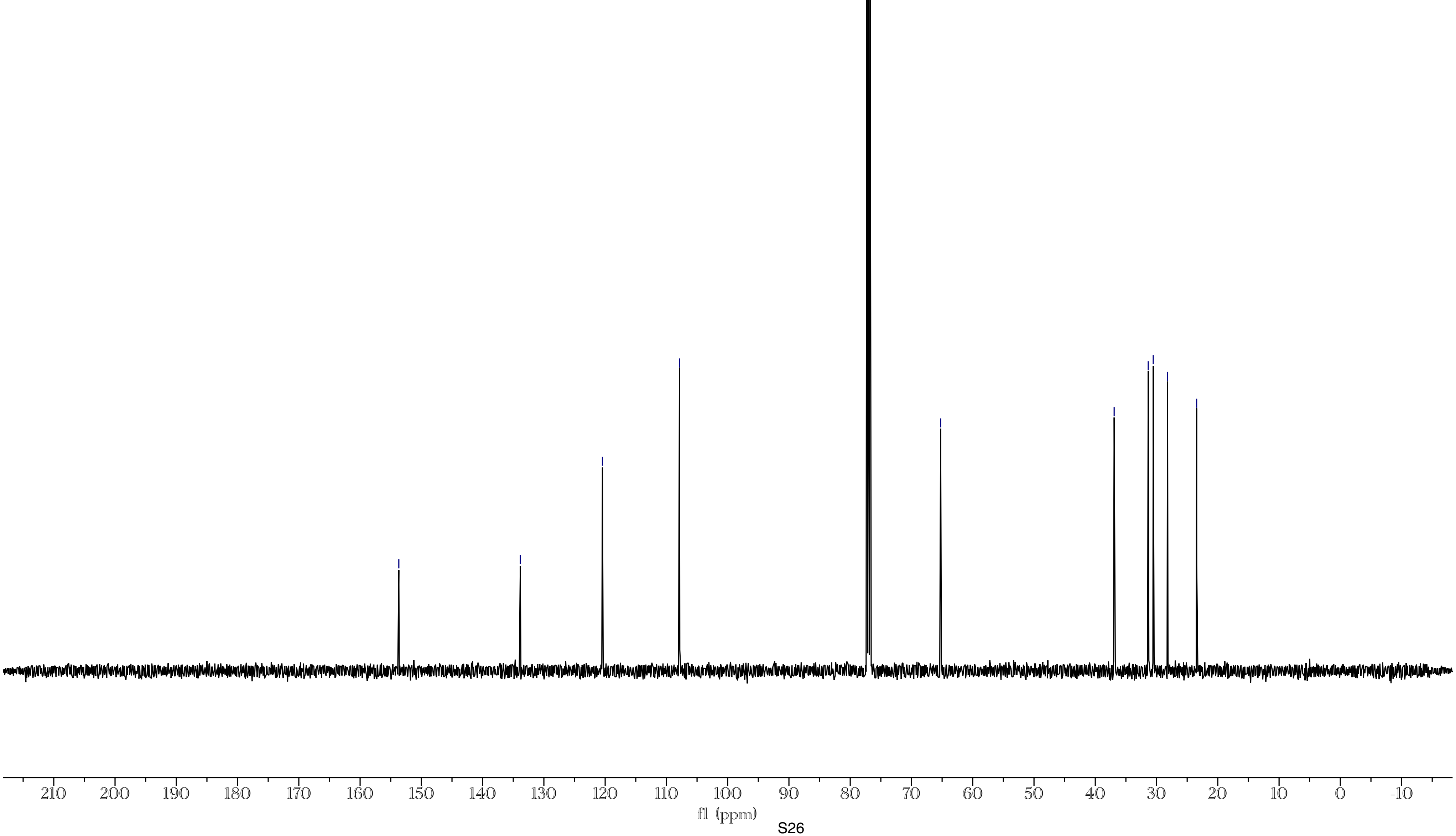

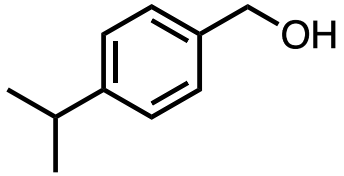

8

^1H NMR (500 MHz, $CDCl_3$)

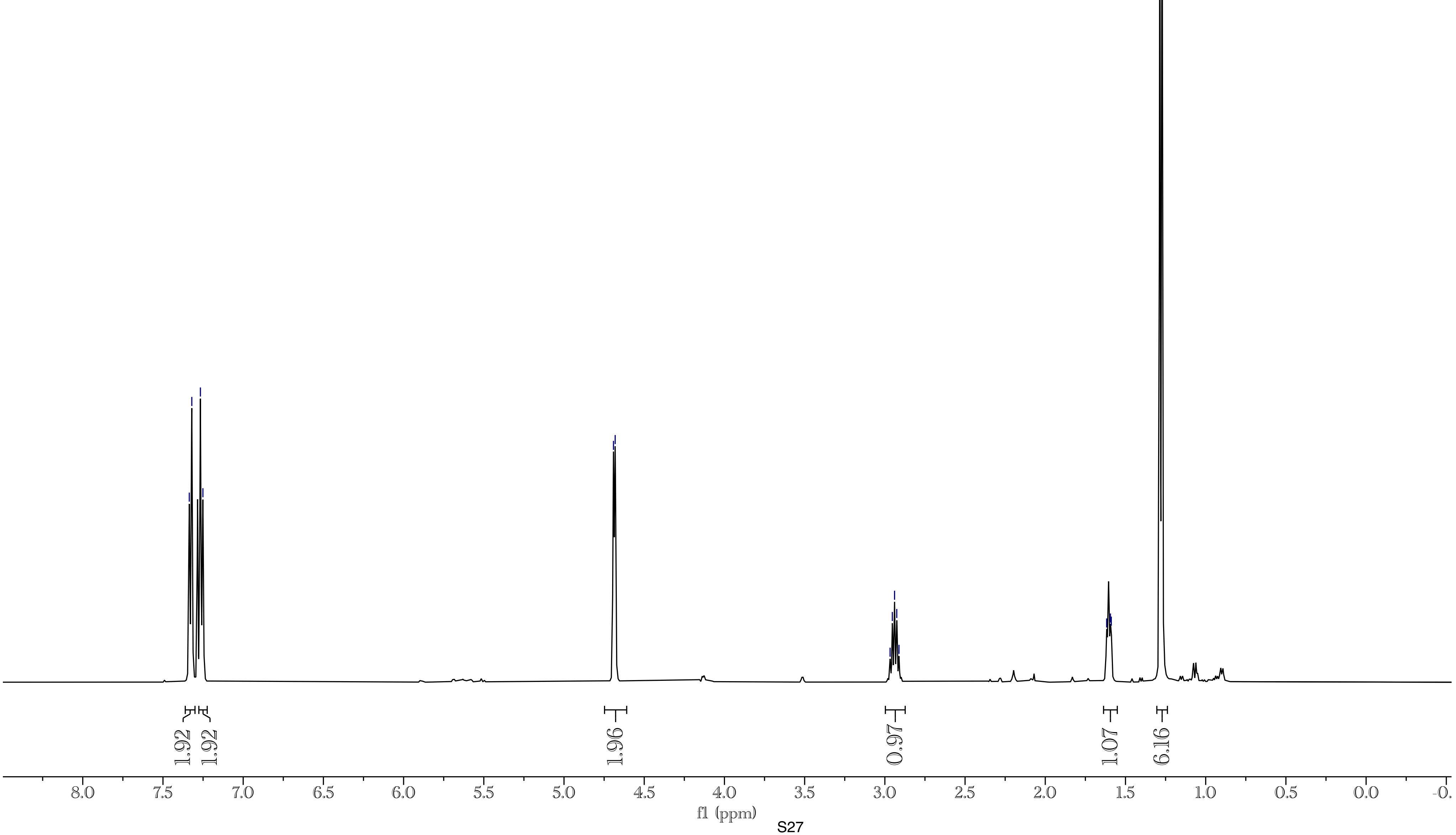

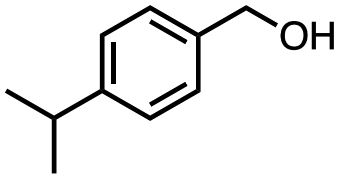

8

^{13}C NMR (126 MHz, $CDCl_3$)

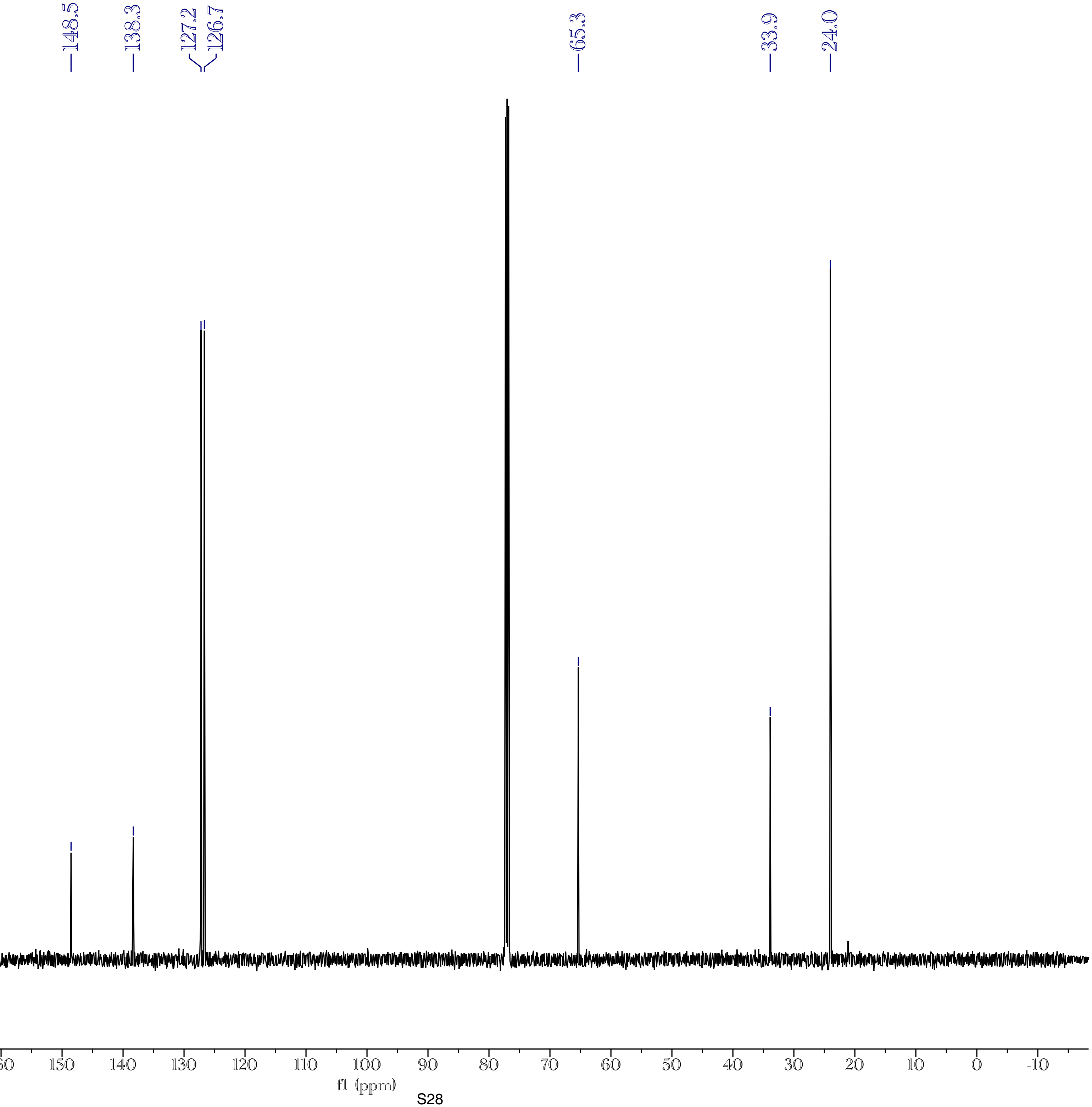

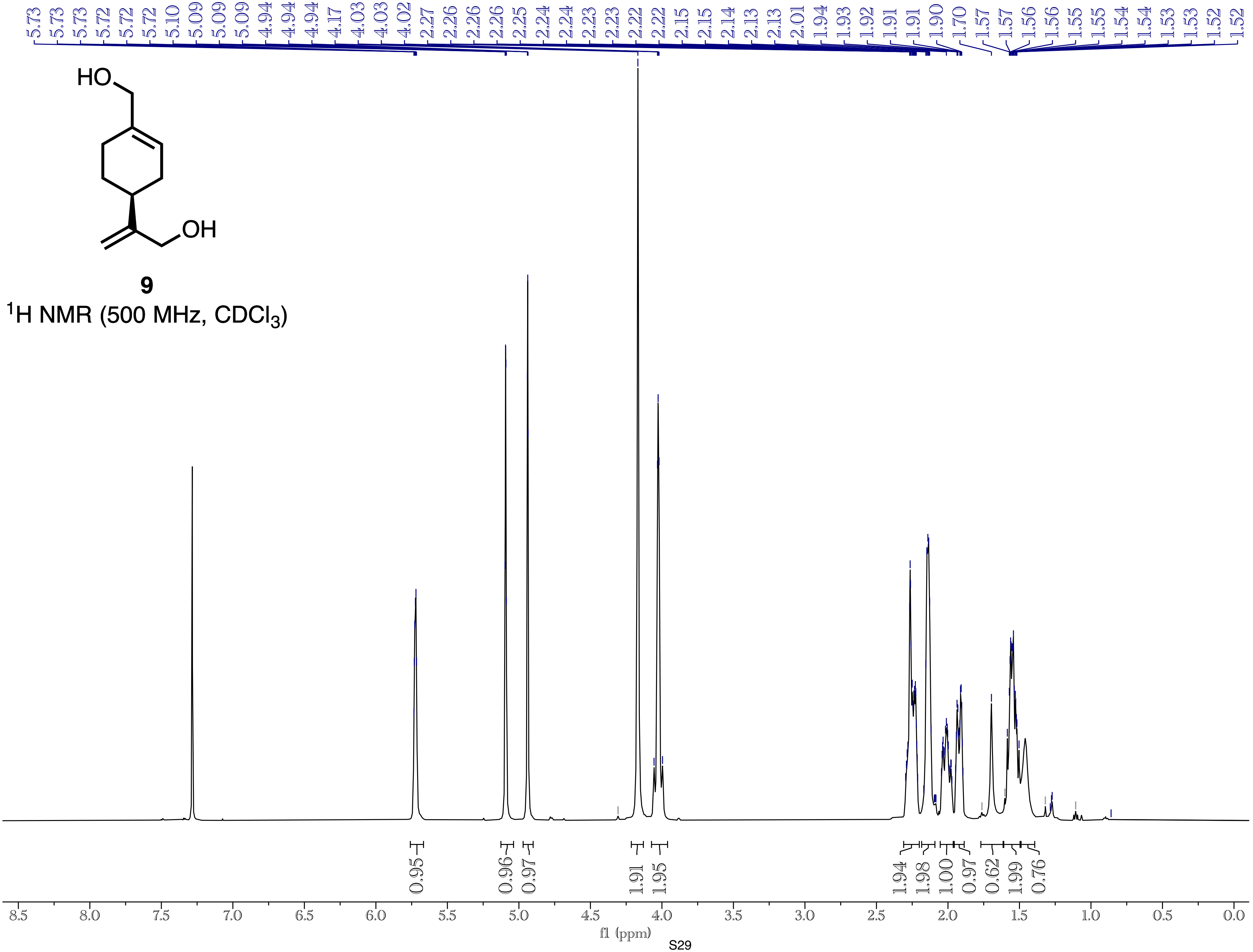

9

^1H NMR (500 MHz, $CDCl_3$)

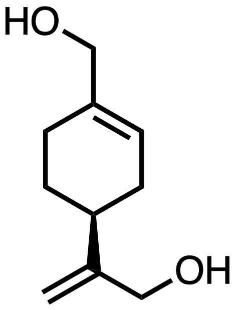

9

^{13}C NMR (126 MHz, $CDCl_3$)

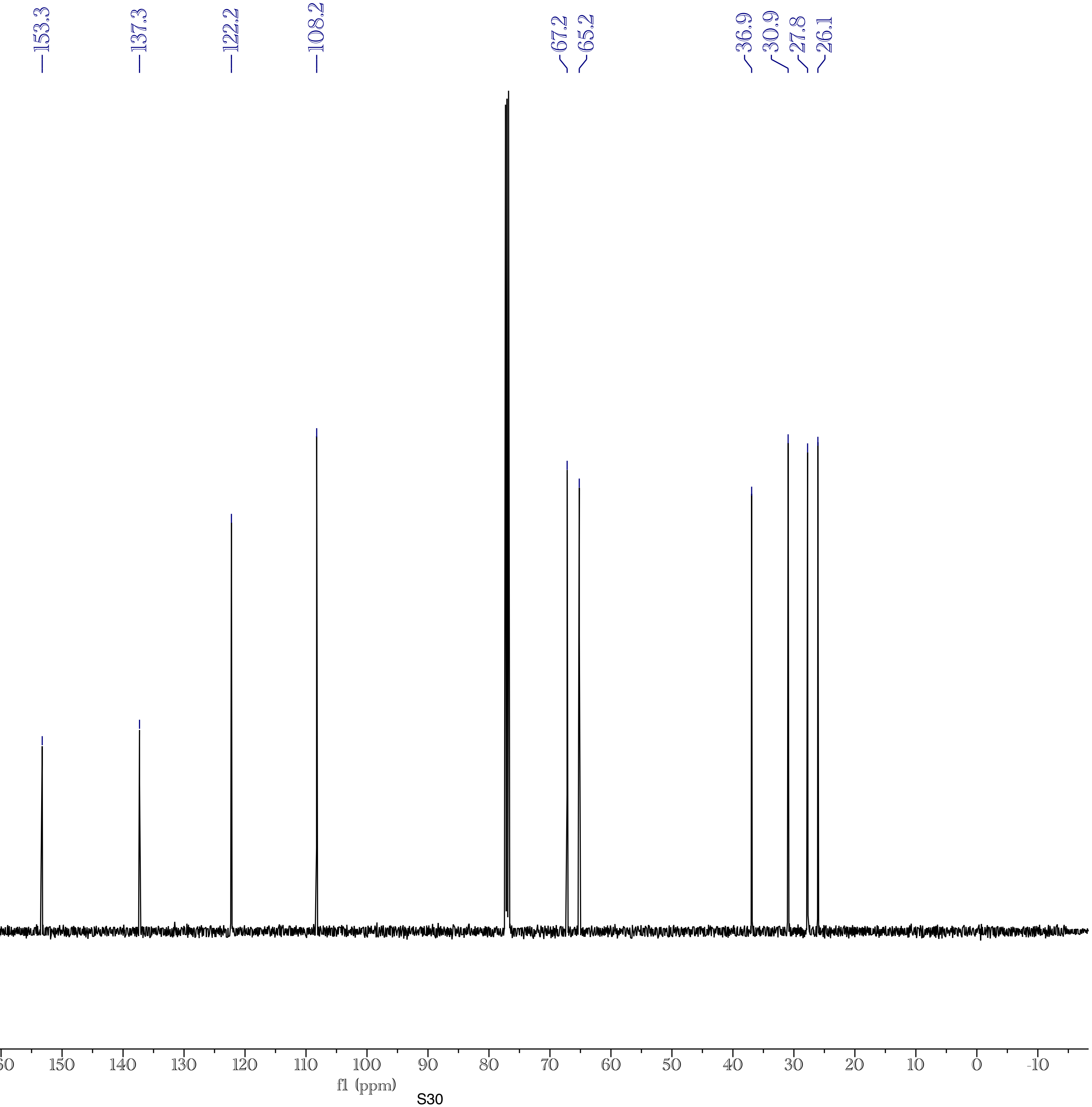

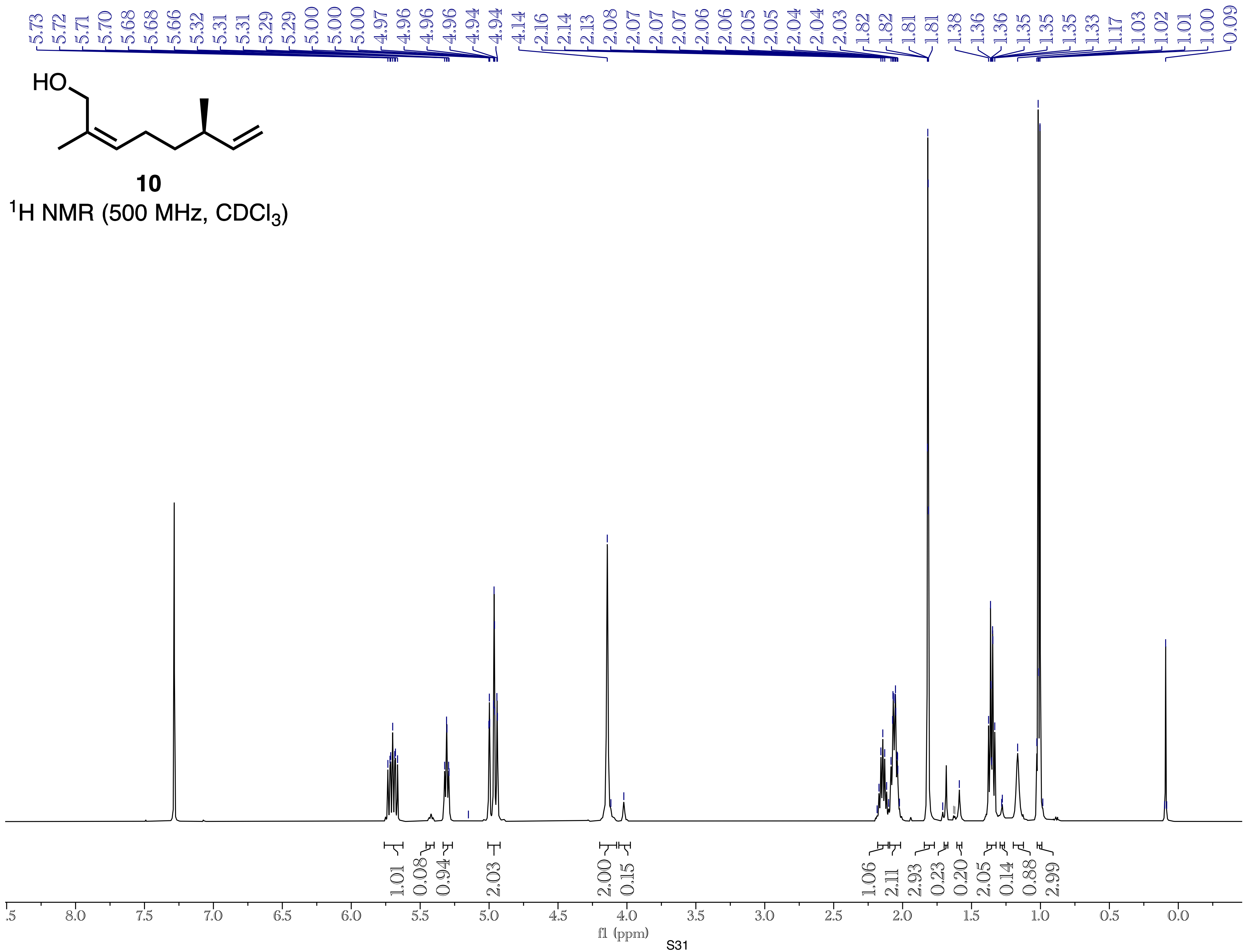

HO
10
^{1}H NMR (500 MHz, $CDCl_3$)
5.73 5.72 5.71 5.70 5.68 5.68 5.66 5.32 5.31 5.31 5.29 5.29 5.00 5.00 5.00 4.97 4.96 4.96 4.96 4.94 4.94 4.14 2.16 2.14 2.13 2.08 2.07 2.07 2.07 2.06 2.06 2.05 2.05 2.04 2.04 2.03 1.82 1.82 1.81 1.81 1.38 1.36 1.36 1.35 1.35 1.35 1.33 1.17 1.03 1.02 1.01 1.00 0.09
1.01 0.08 0.94 2.03 2.00 0.15 1.06 2.11 2.93 0.23 0.20 2.05 0.14 0.88 2.99
.5 8.0 7.5 7.0 6.5 6.0 5.5 5.0 4.5 4.0 3.5 3.0 2.5 2.0 1.5 1.0 0.5 0.0
f1 (ppm)

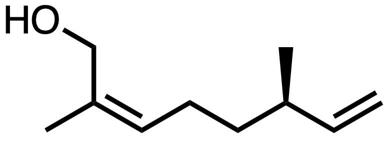

10

^{13}C NMR (126 MHz, $CDCl_3$)

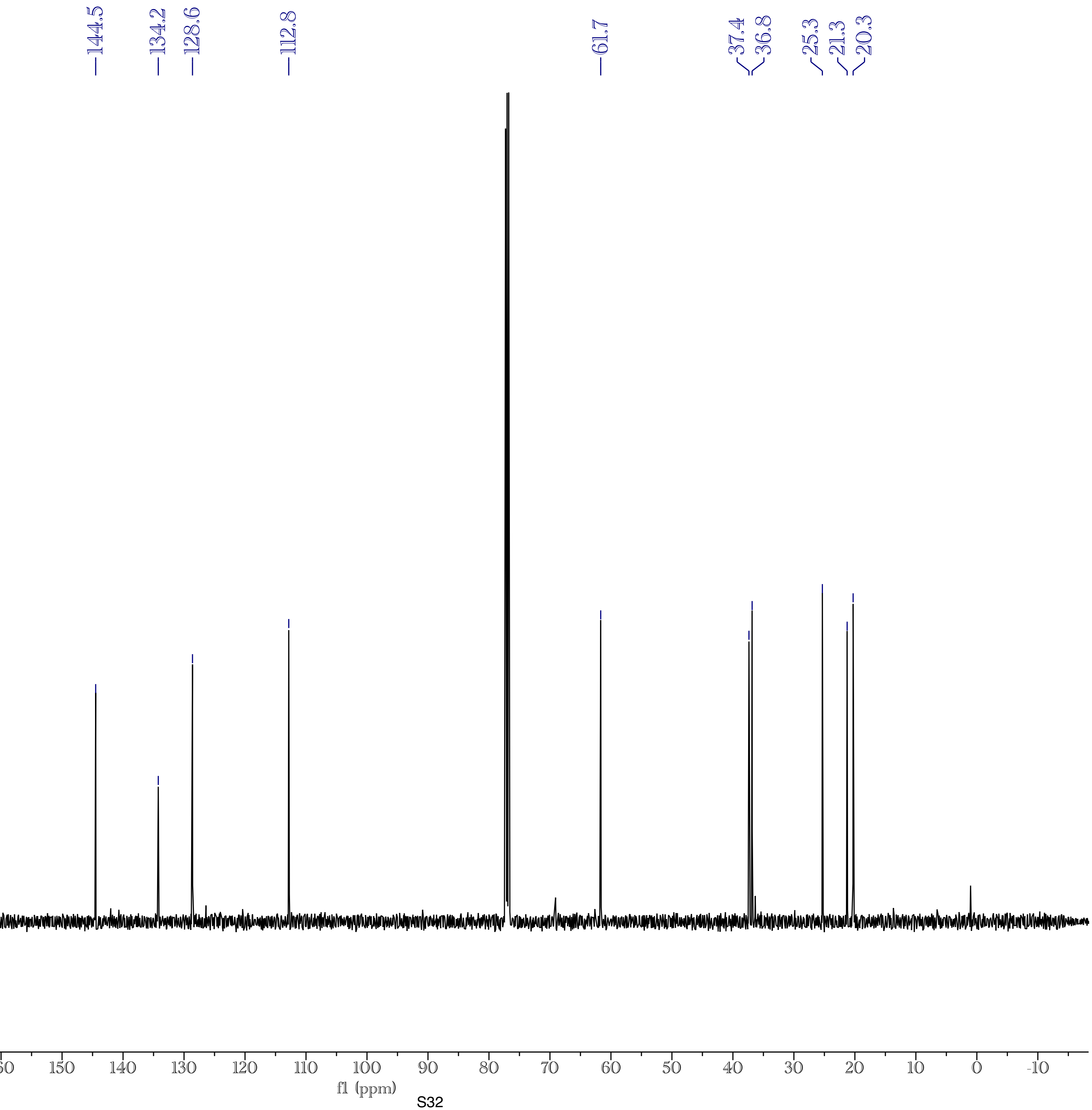

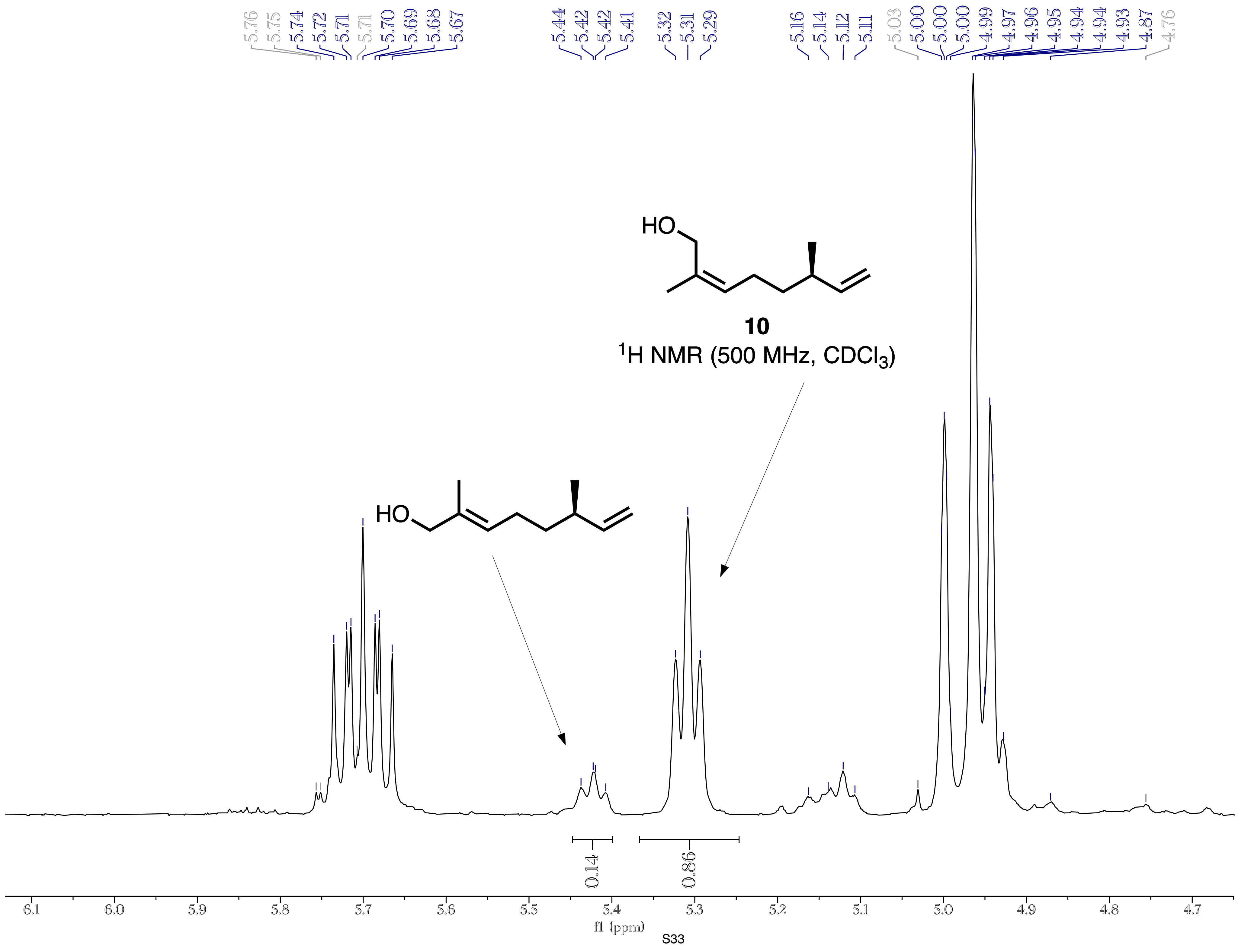

5.76 5.75 5.74 5.72 5.71 5.71 5.70 5.69 5.68 5.67
5.44 5.42 5.42 5.41
5.32 5.31 5.29
5.16 5.14 5.12 5.11
5.03 5.00 5.00 5.00 4.99 4.97 4.96 4.95 4.94 4.94 4.93 4.87 4.76
HO
10
^{1}H NMR (500 MHz, $CDCl_3$)
0.14
0.86
6.1 6.0 5.9 5.8 5.7 5.6 5.5 5.4 5.3 5.2 5.1 5.0 4.9 4.8 4.7
f1 (ppm)

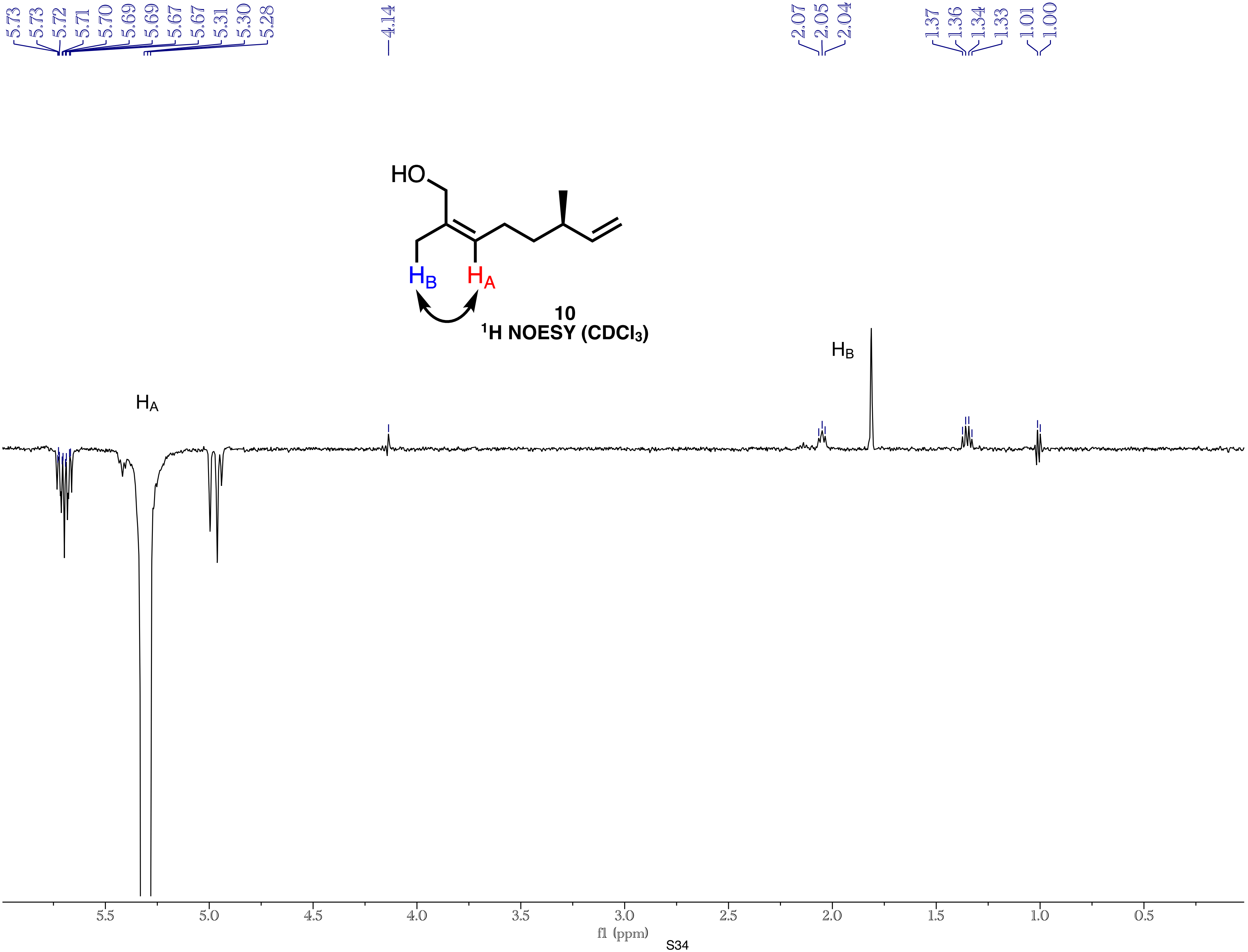

5.73
5.73
5.72
5.71
5.70
5.69
5.69
5.67
5.67
5.31
5.30
5.28
4.14
2.07
2.05
2.04
1.37
1.36
1.34
1.33
1.01
1.00
HO
H$_B$
H$_A$
10
^{1}H NOESY (CDCl$_3$)
H$_B$
H$_A$
5.5
5.0
4.5
4.0
3.5
3.0
2.5
2.0
1.5
1.0
0.5
f1 (ppm)

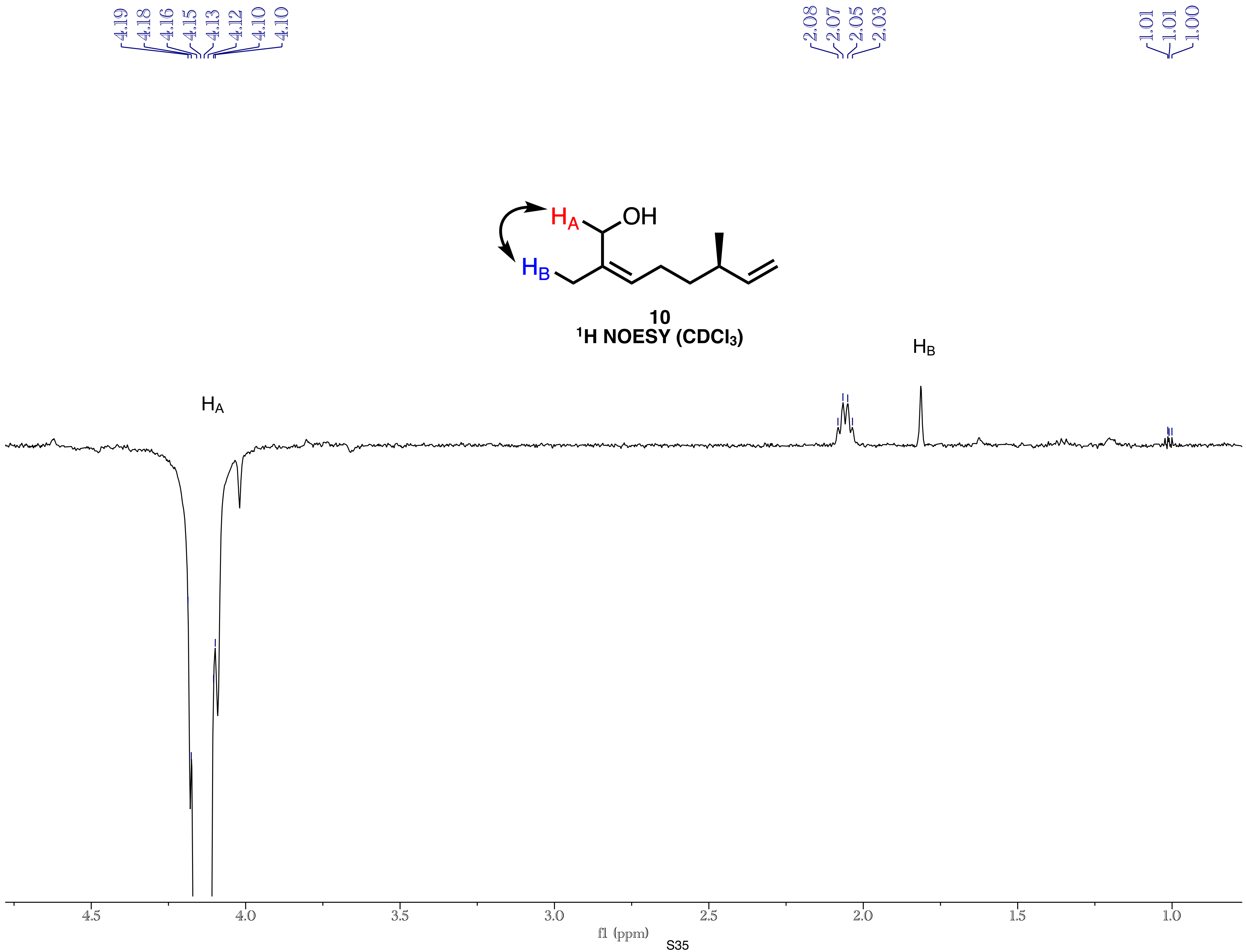
4.19
4.18
4.16
4.15
4.13
4.12
4.10
4.10
2.08
2.07
2.05
2.03
1.01
1.01
1.00
H_A
OH
H_B
10
1H NOESY (CDCl3)
H_B
H_A
4.5
4.0
3.5
3.0
2.5
2.0
1.5
1.0
f1 (ppm)

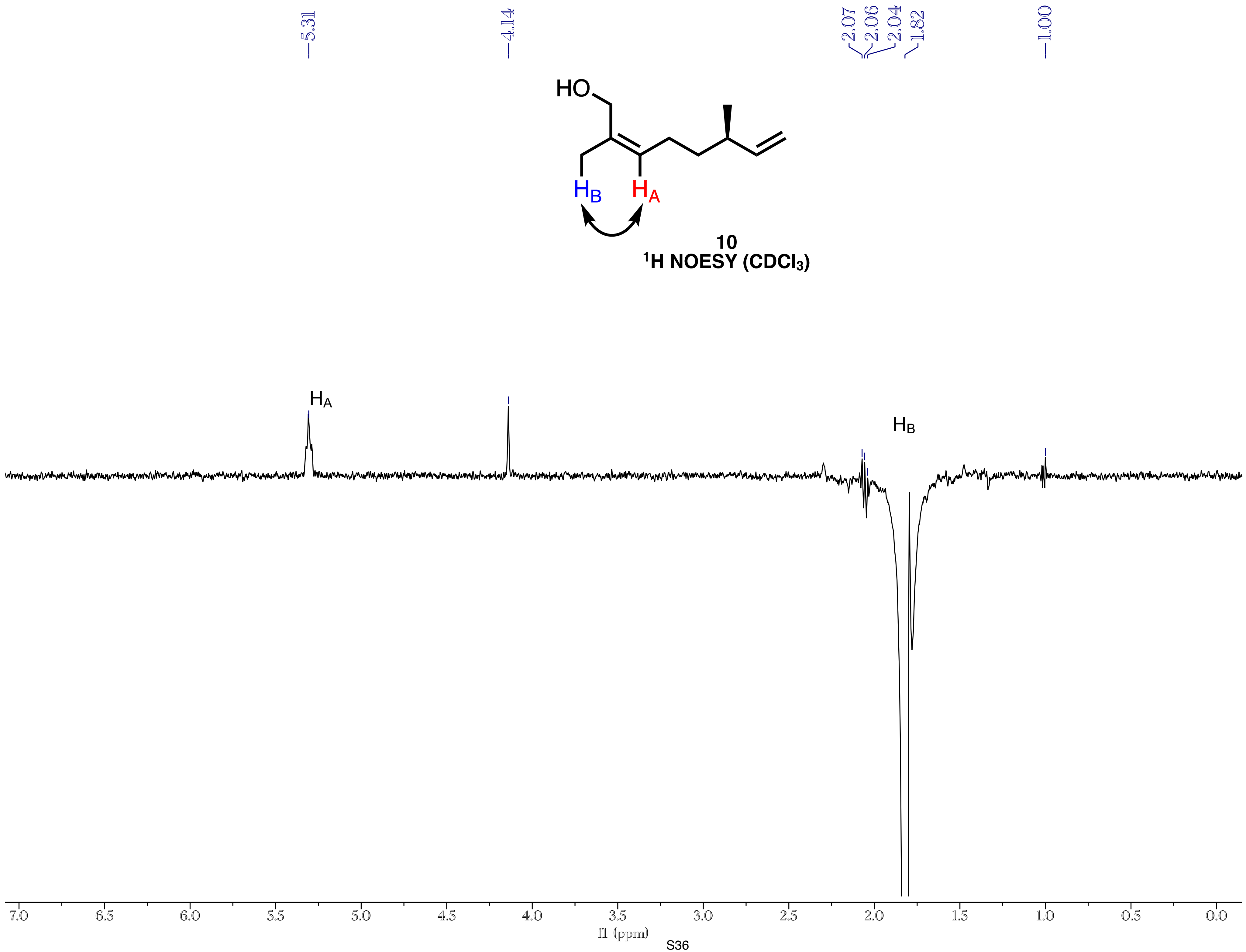
5.31
4.14
2.07
2.06
2.04
1.82
1.00
HO
H_B
H_A
10
^{1}H NOESY ($CDCl_3$)
H_A
H_B
7.0
6.5
6.0
5.5
5.0
4.5
4.0
3.5
3.0
2.5
2.0
1.5
1.0
0.5
0.0
f1 (ppm)

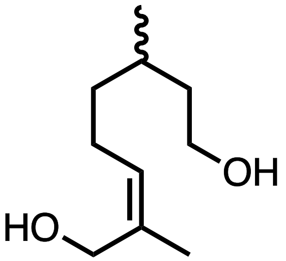

11

^{1}H NMR (500 MHz, $CDCl_3$)

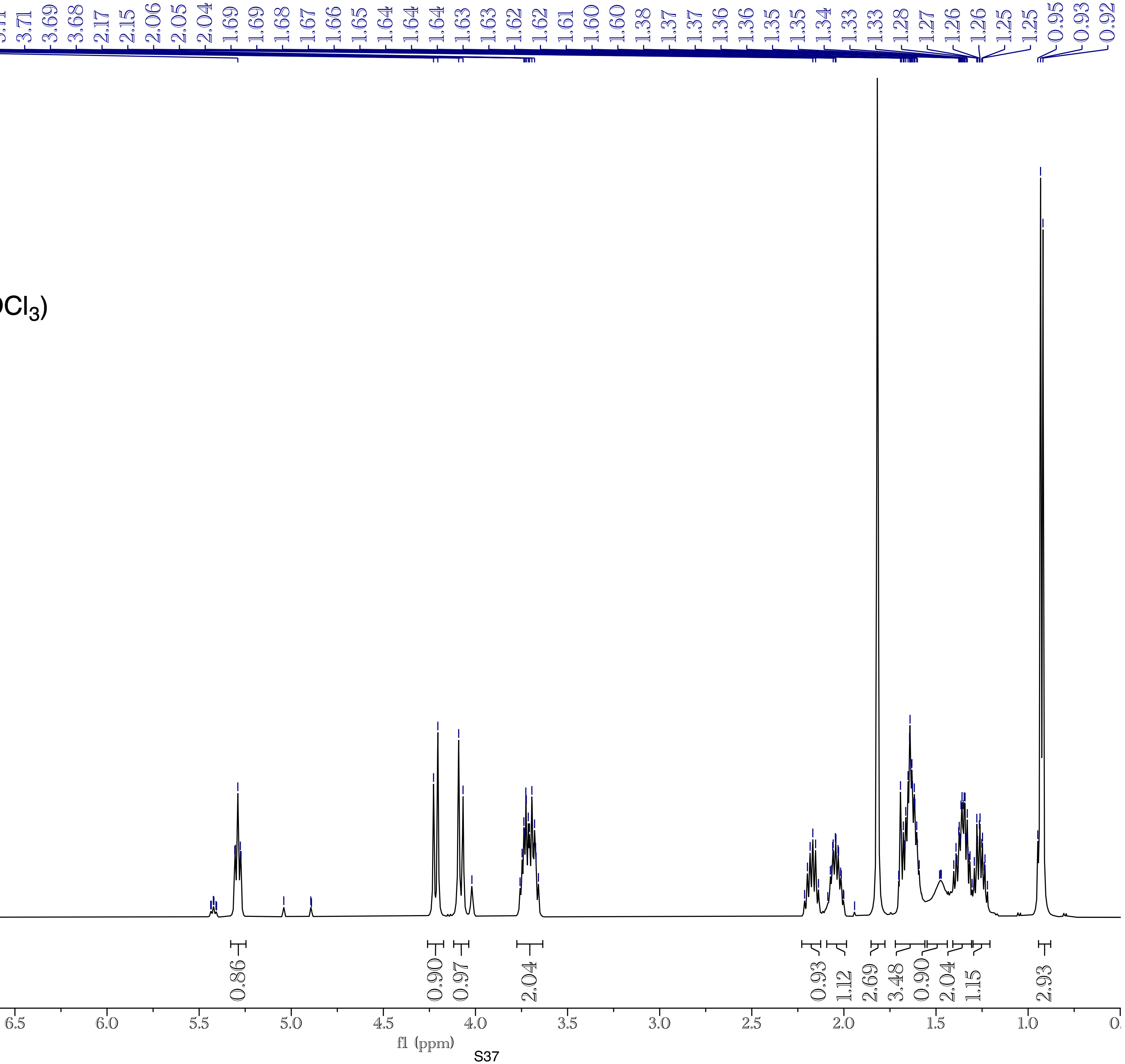

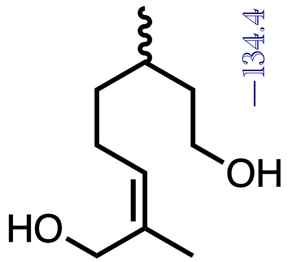

11

^{13}C NMR (126 MHz, $CDCl_3$)

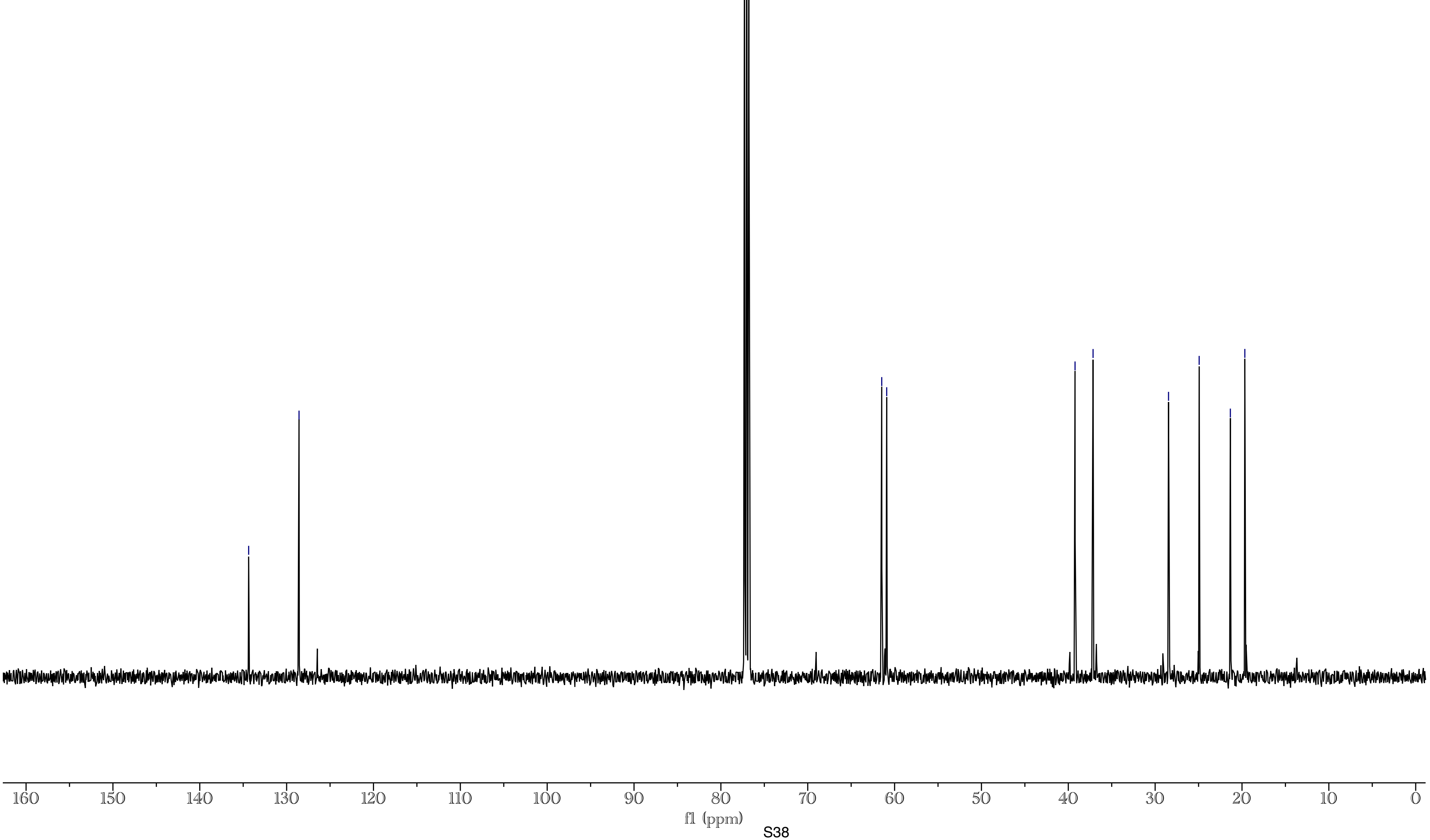

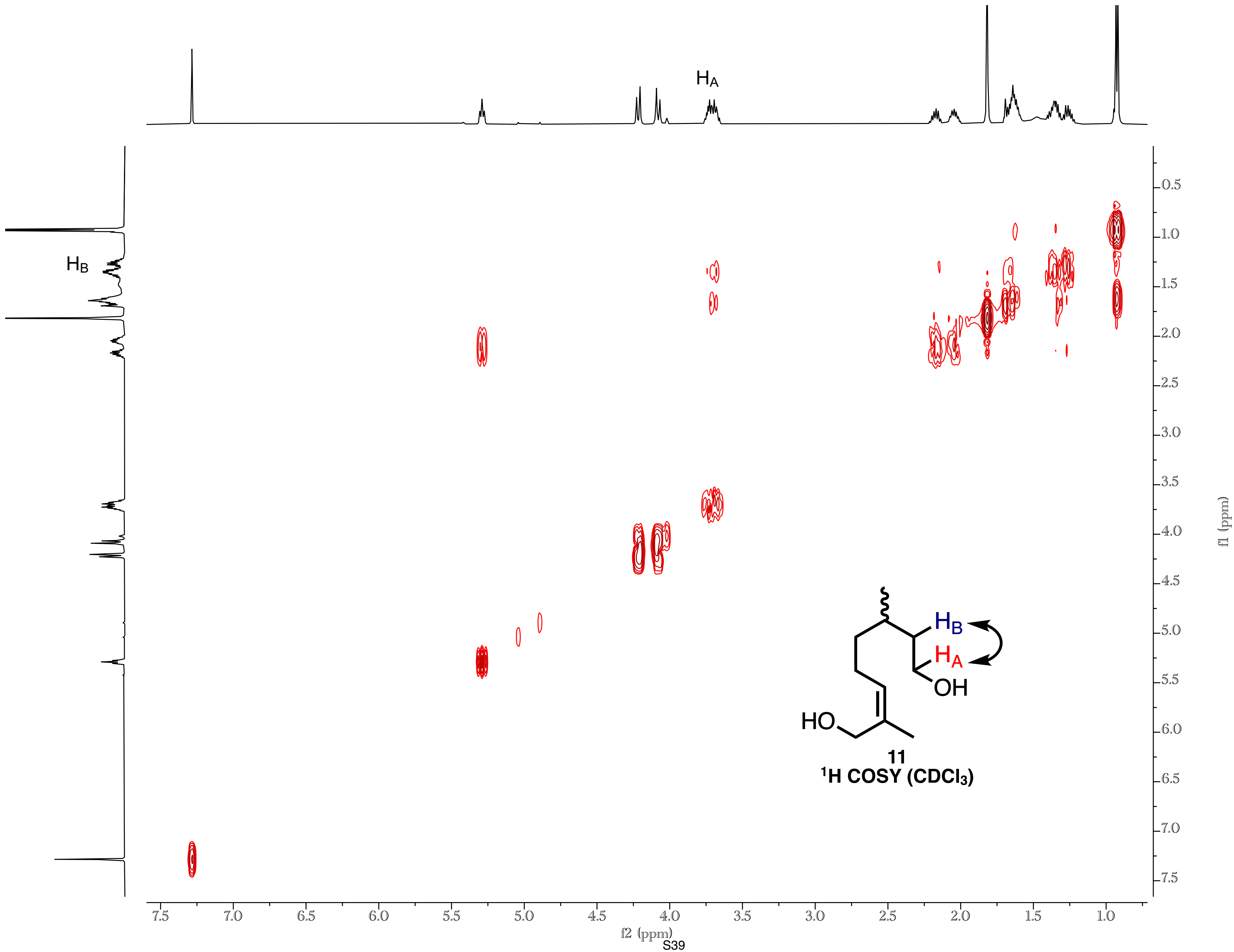

HA
HB
f2 (ppm)
f1 (ppm)
HB
HA
OH
HO
11
1H COSY (CDCl3)

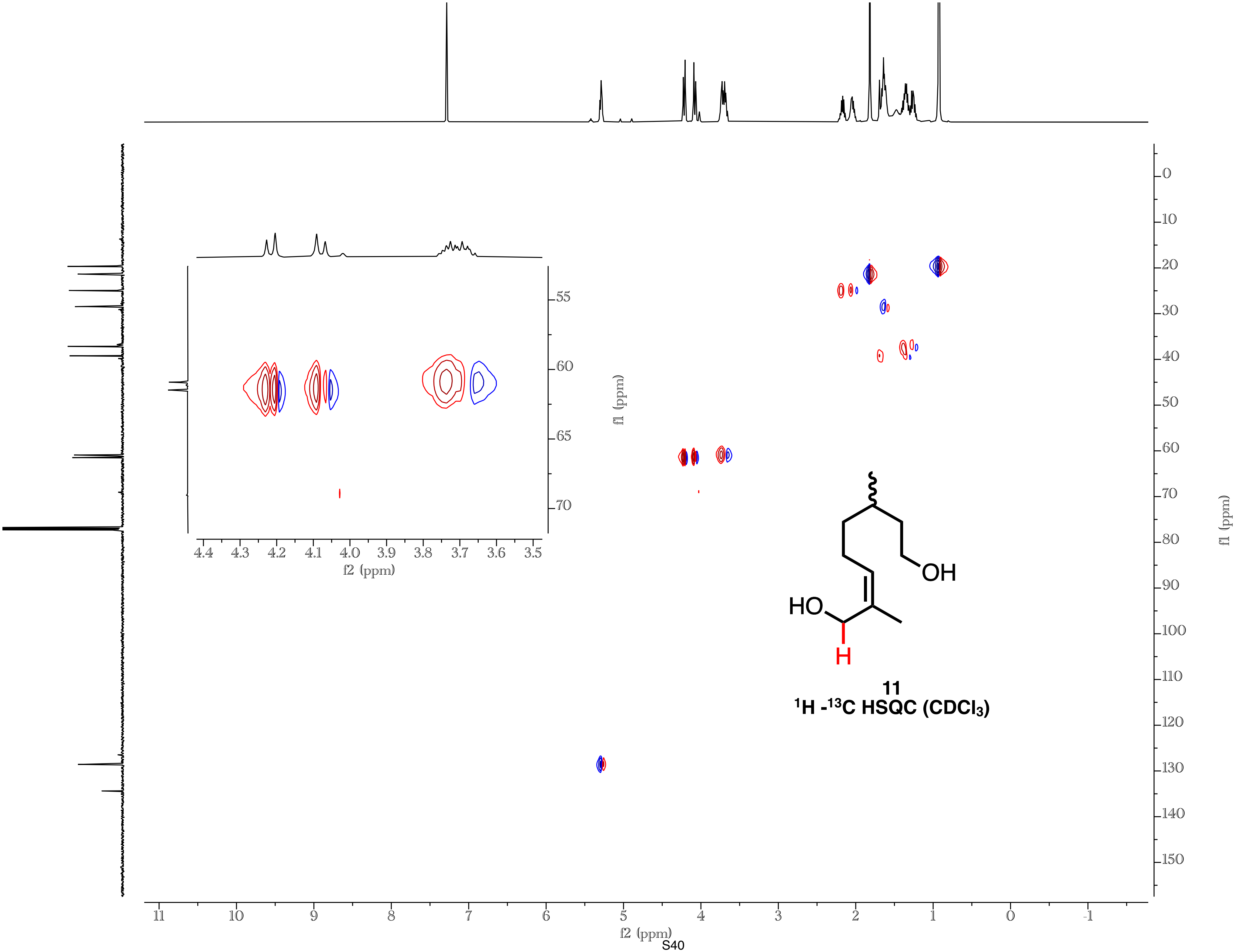

11

^{1}H -^{13}C HSQC ($CDCl_3$)

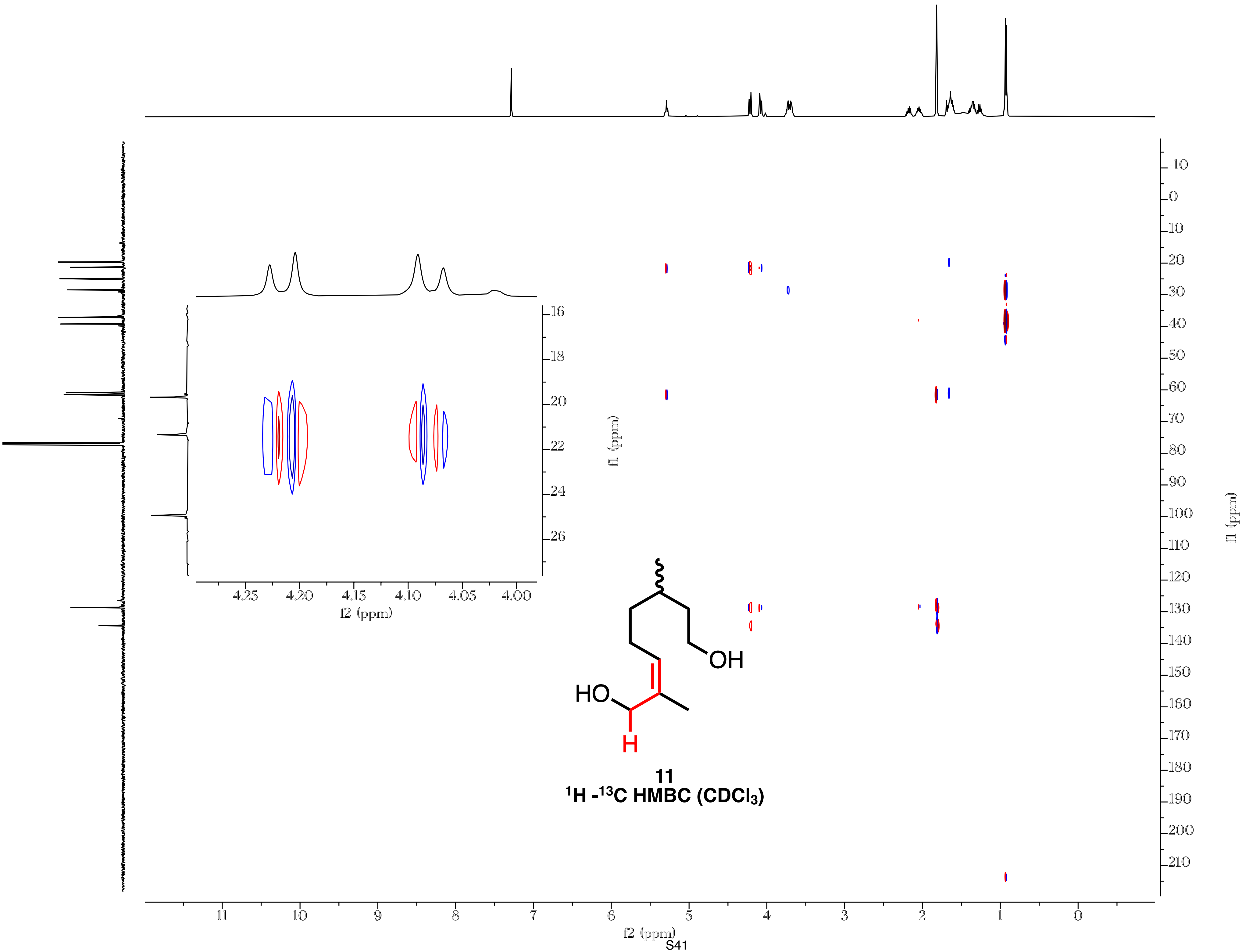

11
^{1}H -^{13}C HMBC ($CDCl_3$)

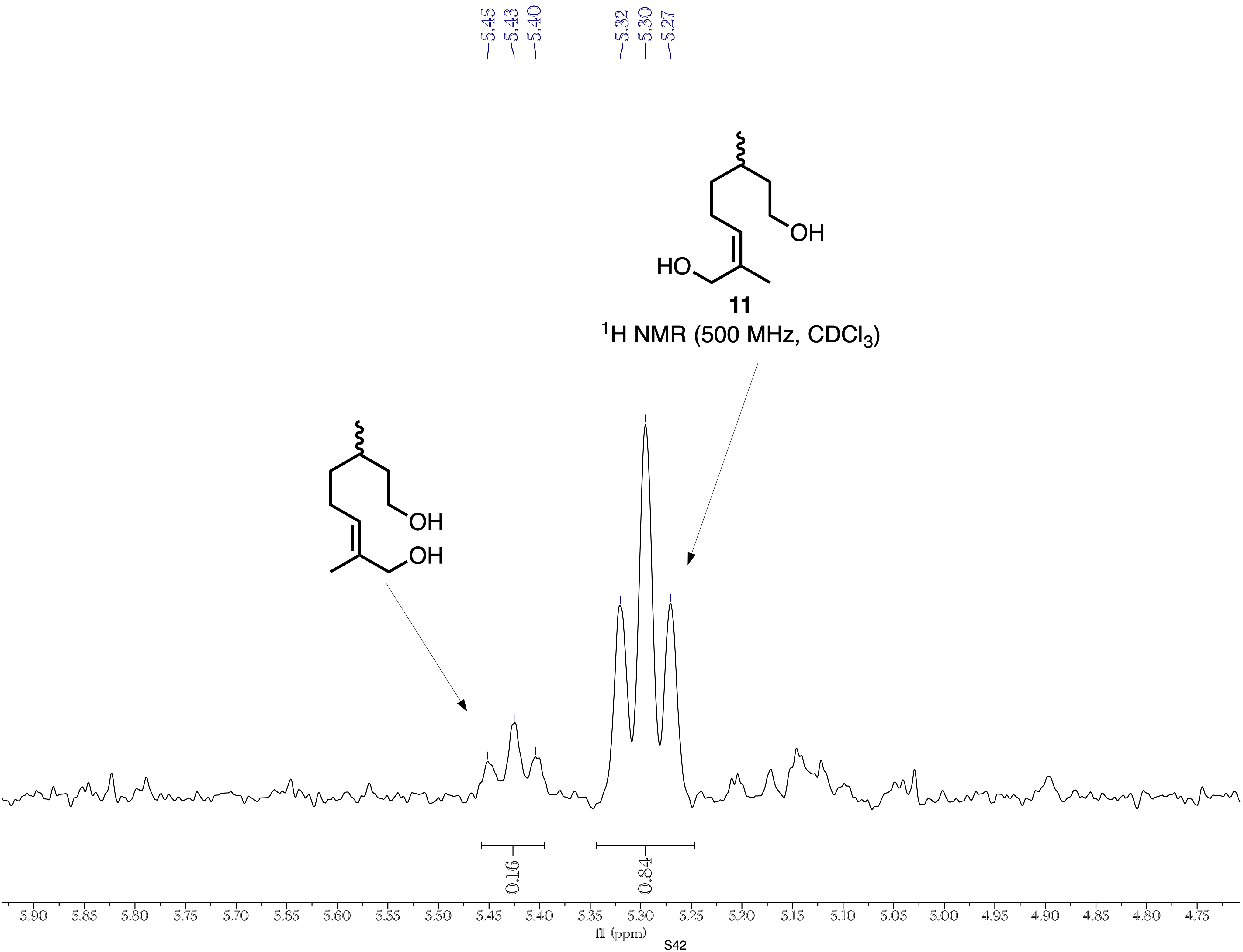

11

^{1}H NMR (500 MHz, $CDCl_3$)

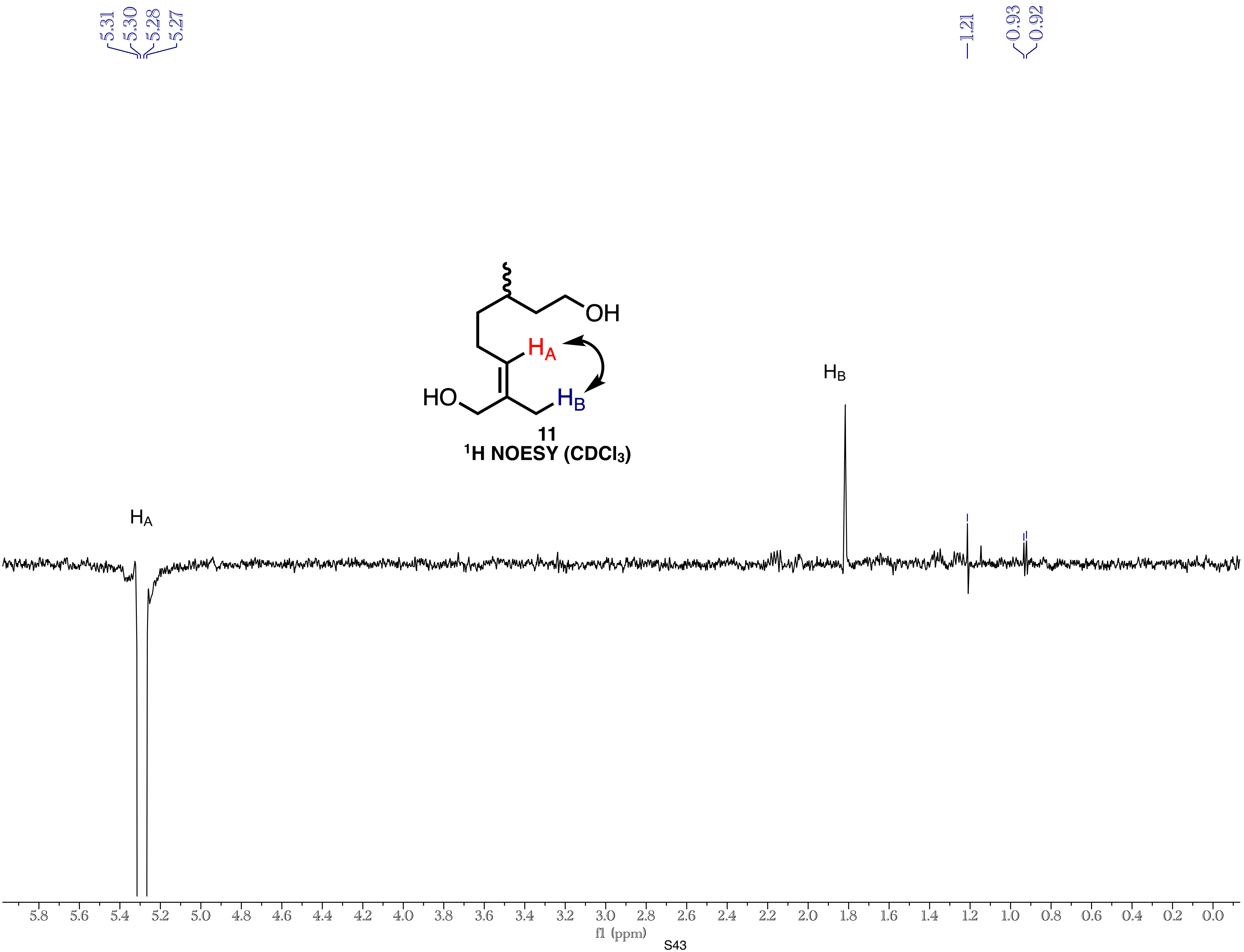
5.31
5.30
5.28
5.27
1.21
0.93
0.92
OH
HO
H_A
H_B
11
1H NOESY (CDCl3)
H_A
H_B
5.8 5.6 5.4 5.2 5.0 4.8 4.6 4.4 4.2 4.0 3.8 3.6 3.4 3.2 3.0 2.8 2.6 2.4 2.2 2.0 1.8 1.6 1.4 1.2 1.0 0.8 0.6 0.4 0.2 0.0
f1 (ppm)

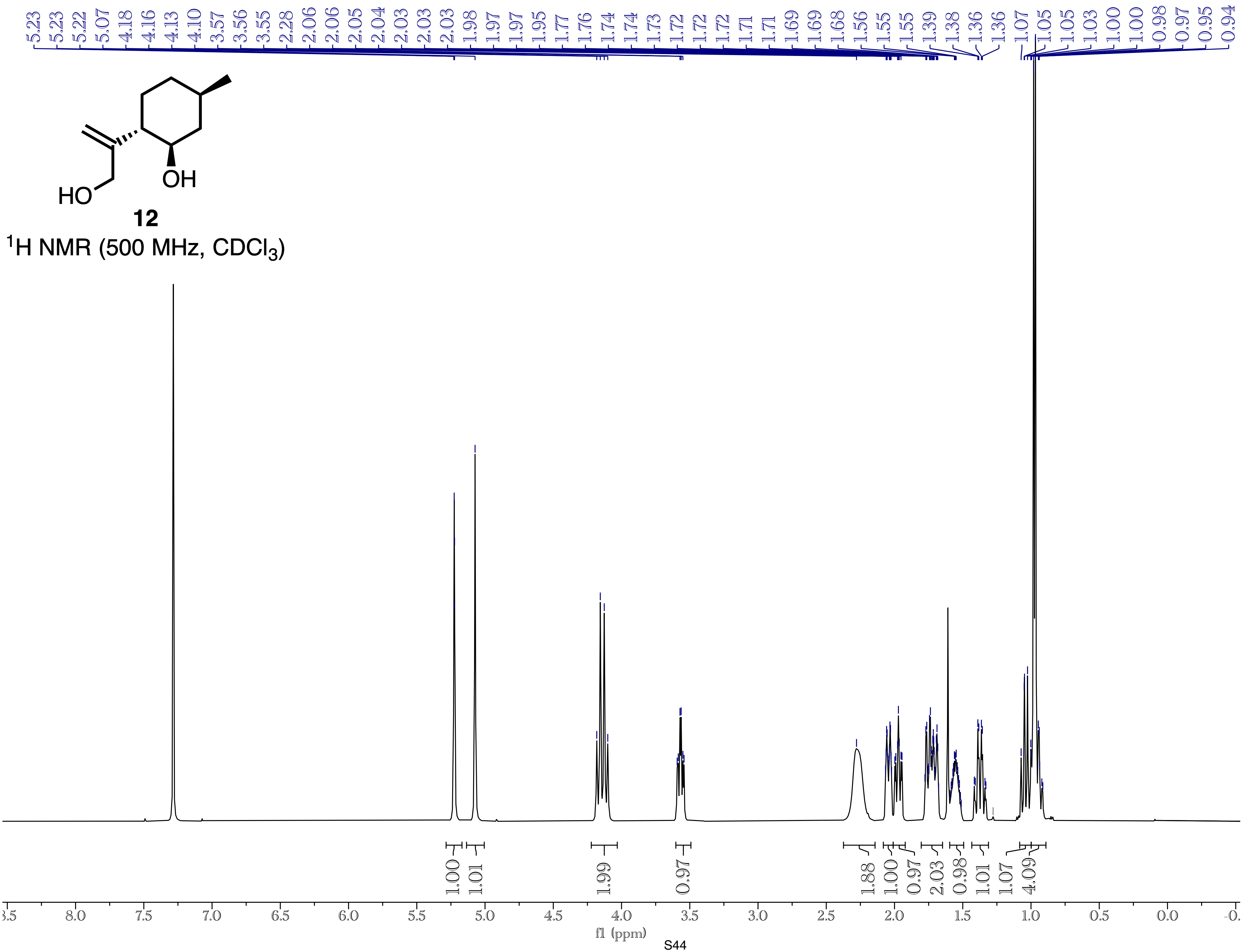
HO
OH
12
^{1}H NMR (500 MHz, $CDCl_3$)
fl (ppm)

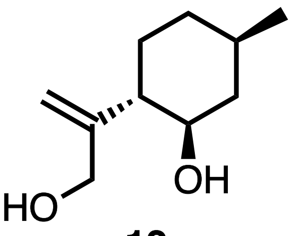

12

^{13}C NMR (126 MHz, $CDCl_3$)

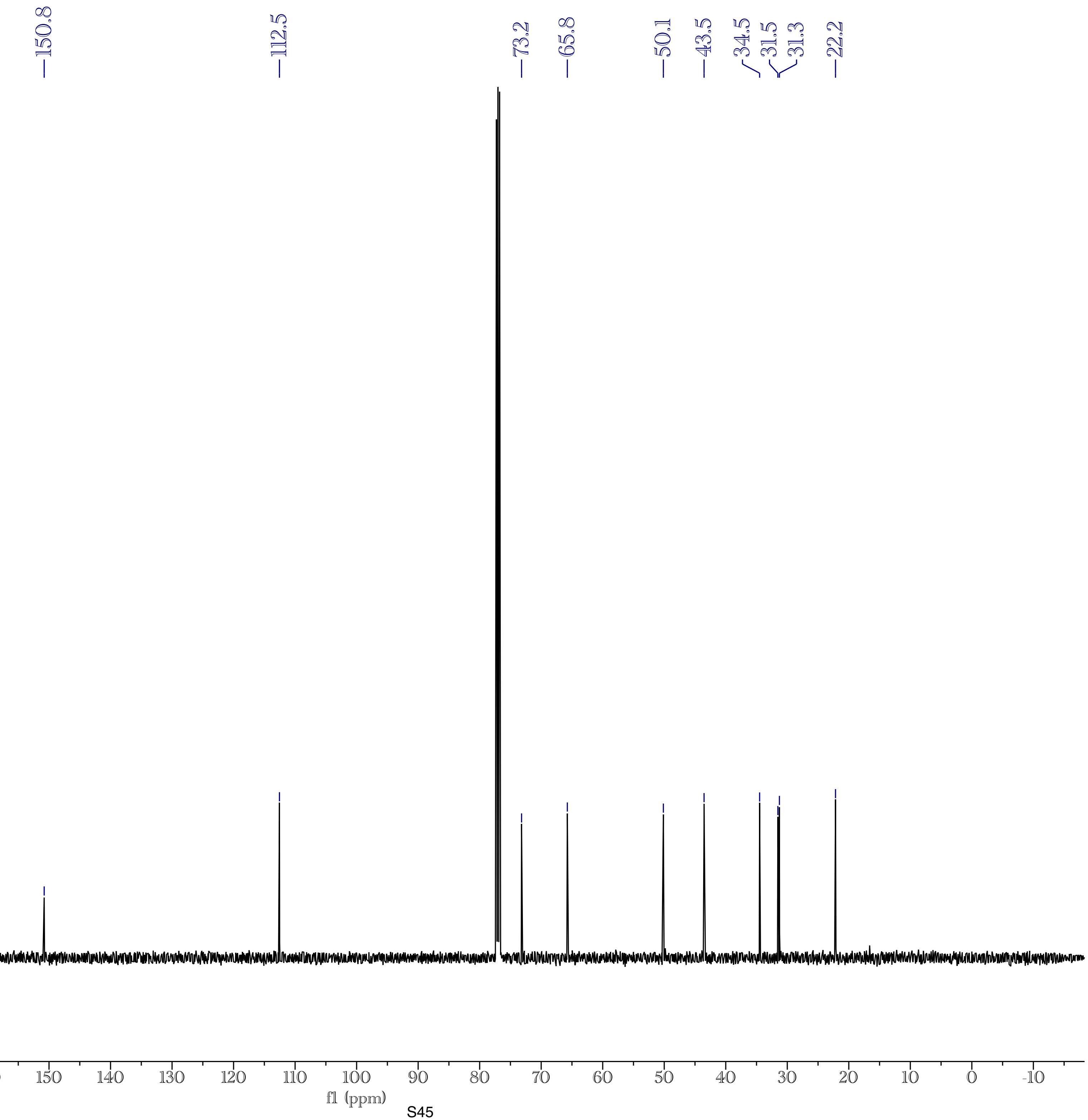

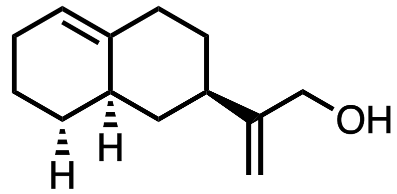

13

^{1}H NMR (500 MHz, $CDCl_3$)

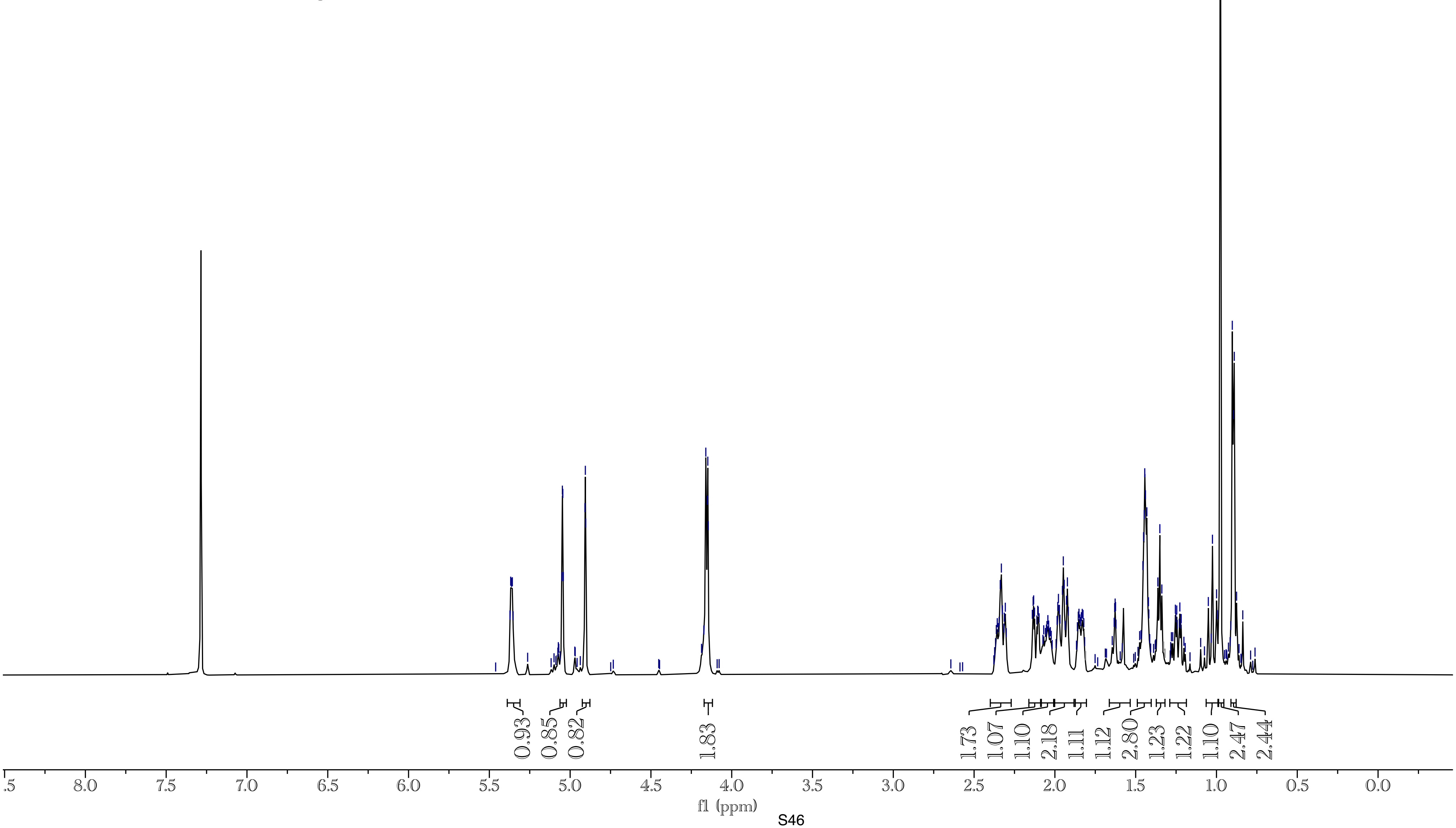

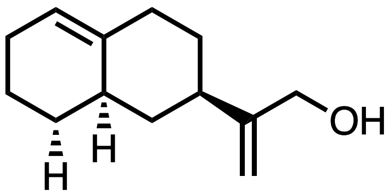

13

^{13}C NMR (126 MHz, $CDCl_3$)

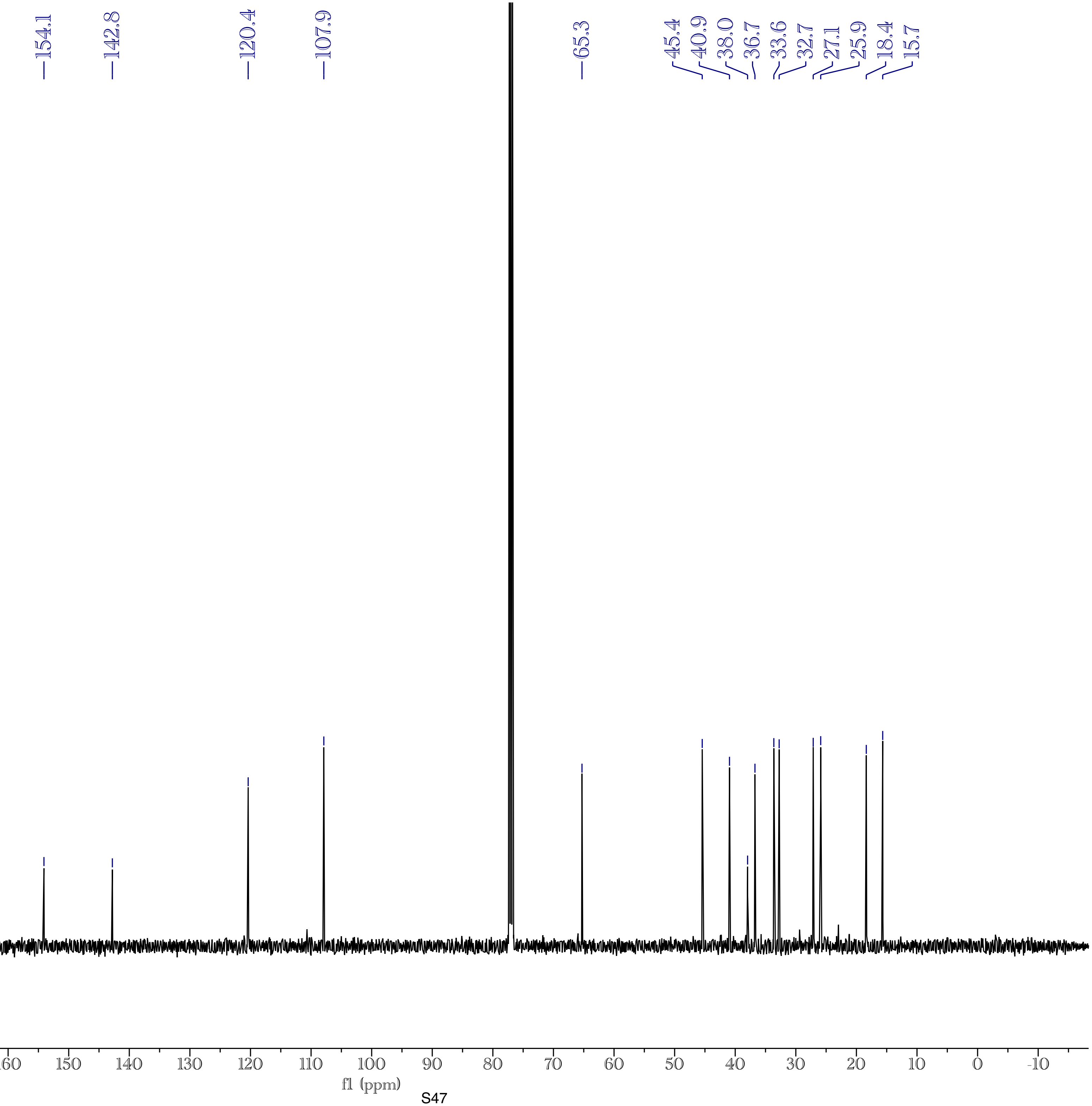

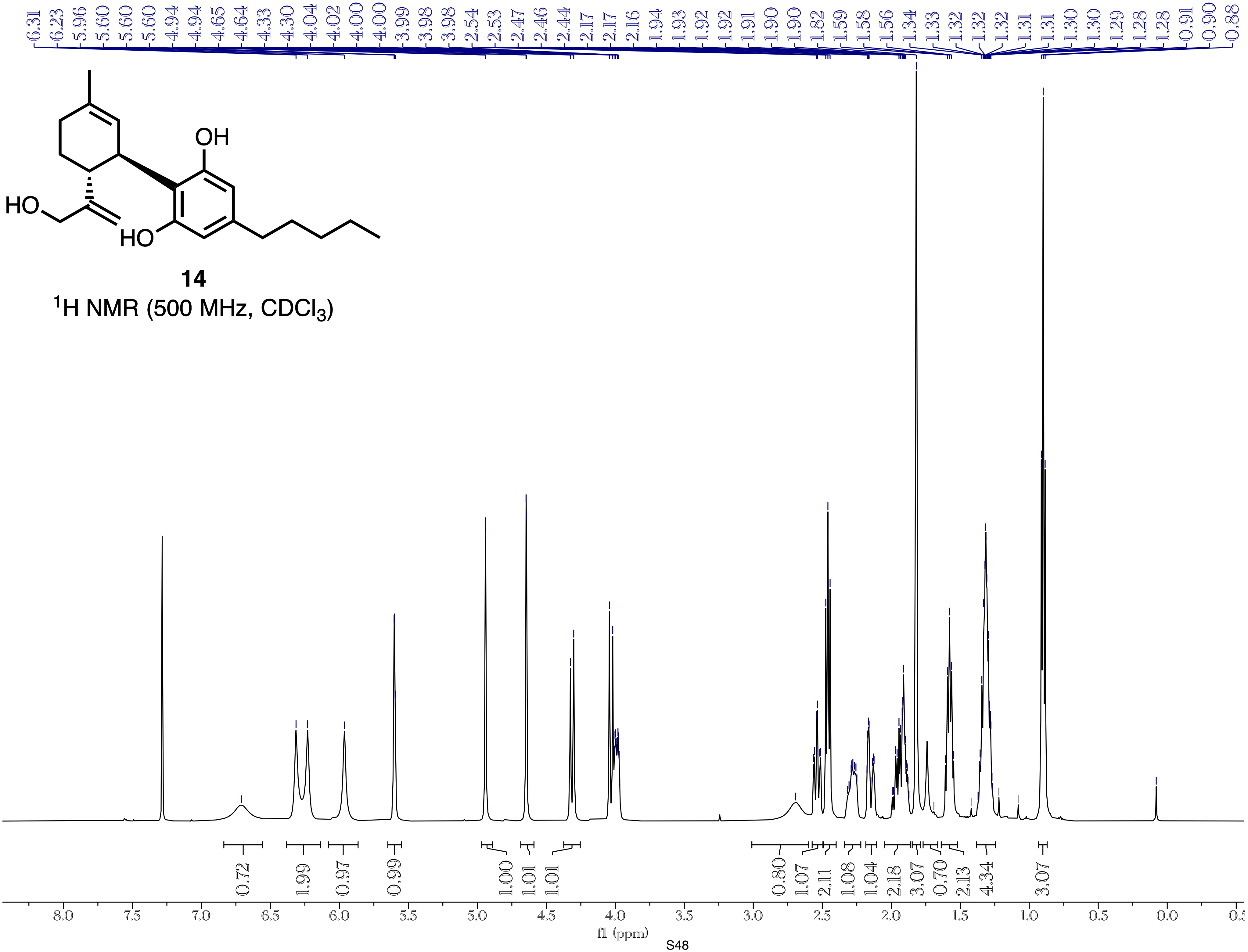

6.31 6.23 5.96 5.60 5.60 5.60 4.94 4.94 4.65 4.64 4.33 4.30 4.04 4.02 4.00 4.00 3.99 3.98 3.98 2.54 2.53 2.47 2.46 2.44 2.17 2.17 2.16 1.94 1.93 1.92 1.92 1.91 1.90 1.90 1.82 1.59 1.58 1.56 1.34 1.33 1.32 1.32 1.32 1.31 1.31 1.30 1.30 1.29 1.28 1.28 0.91 0.90 0.88
OH
HO
HO
14
^{1}H NMR (500 MHz, $CDCl_3$)
0.72 1.99 0.97 0.99 1.00 1.01 1.01 0.80 1.07 2.11 1.08 1.04 2.18 3.07 0.70 2.13 4.34 3.07
8.0 7.5 7.0 6.5 6.0 5.5 5.0 4.5 4.0 3.5 3.0 2.5 2.0 1.5 1.0 0.5 0.0
f1 (ppm)

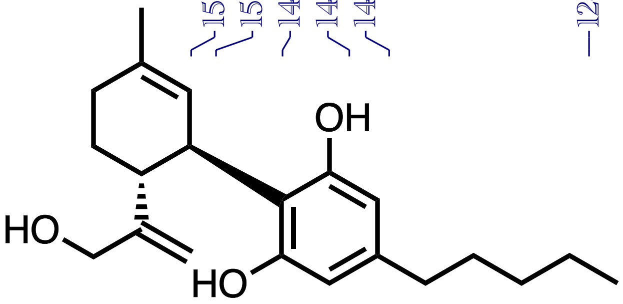

14

^{13}C NMR (126 MHz, $CDCl_3$)

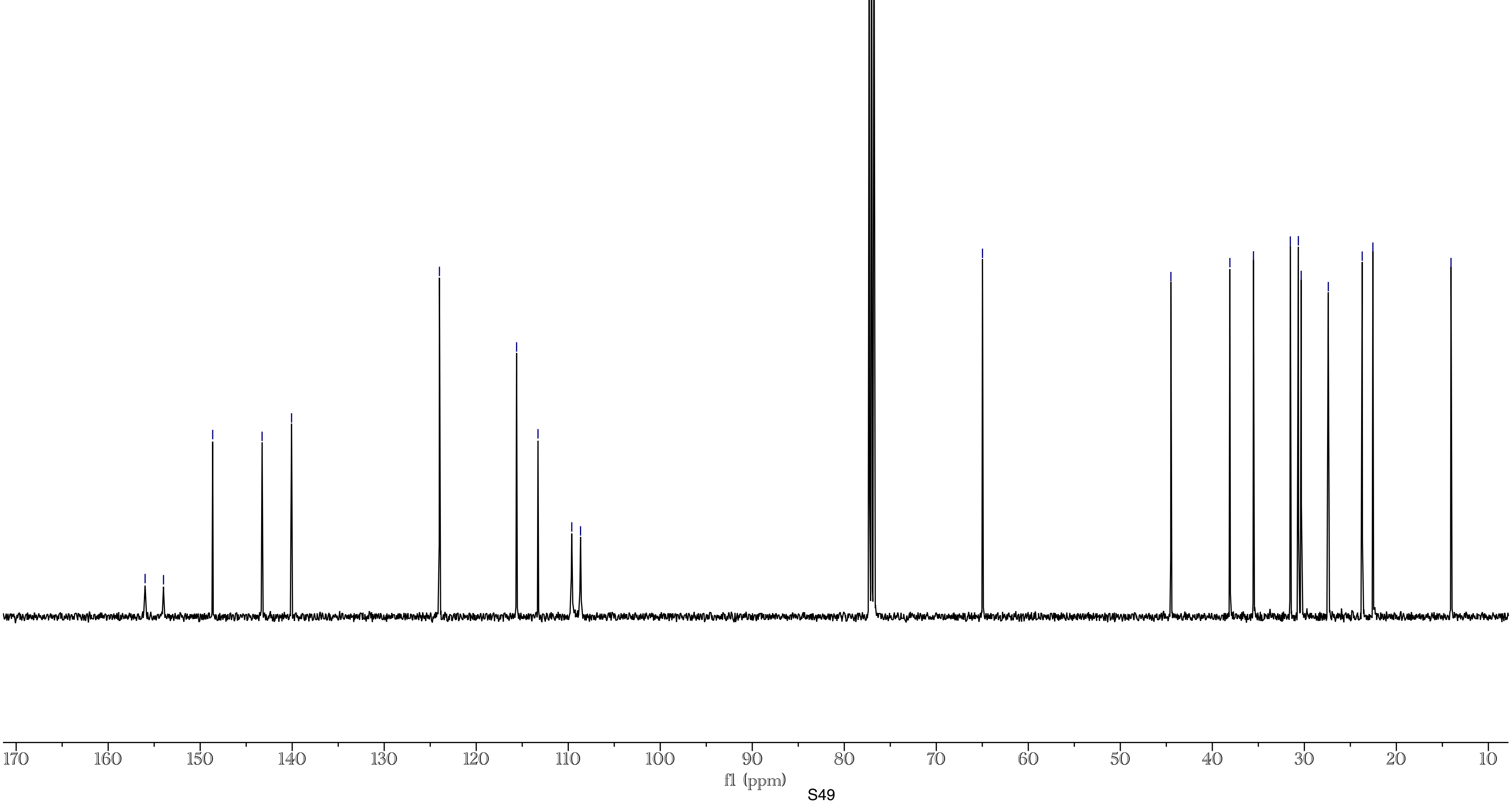

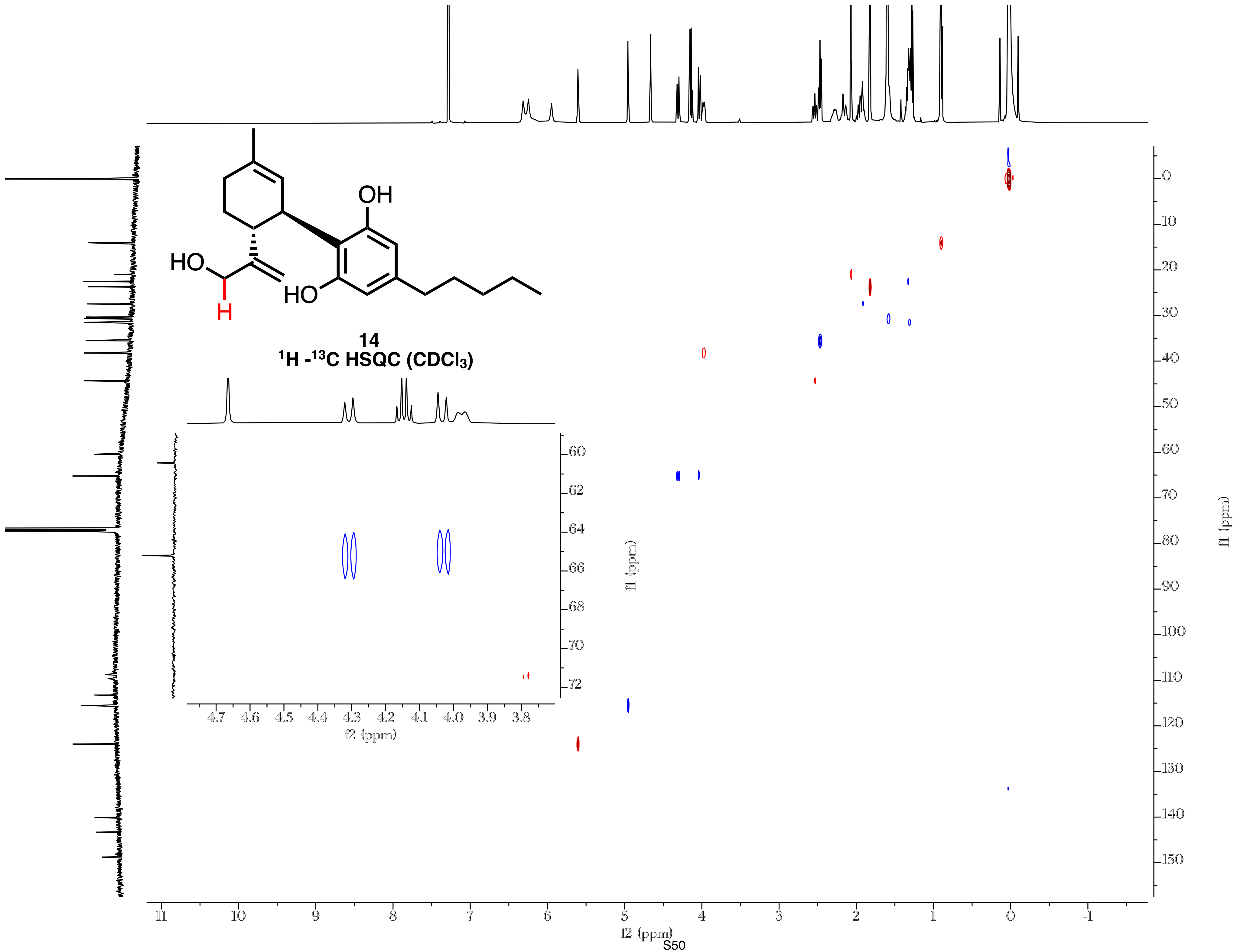
OH
HO
HO
H
14
1H -13C HSQC (CDCl3)
f2 (ppm)
f1 (ppm)

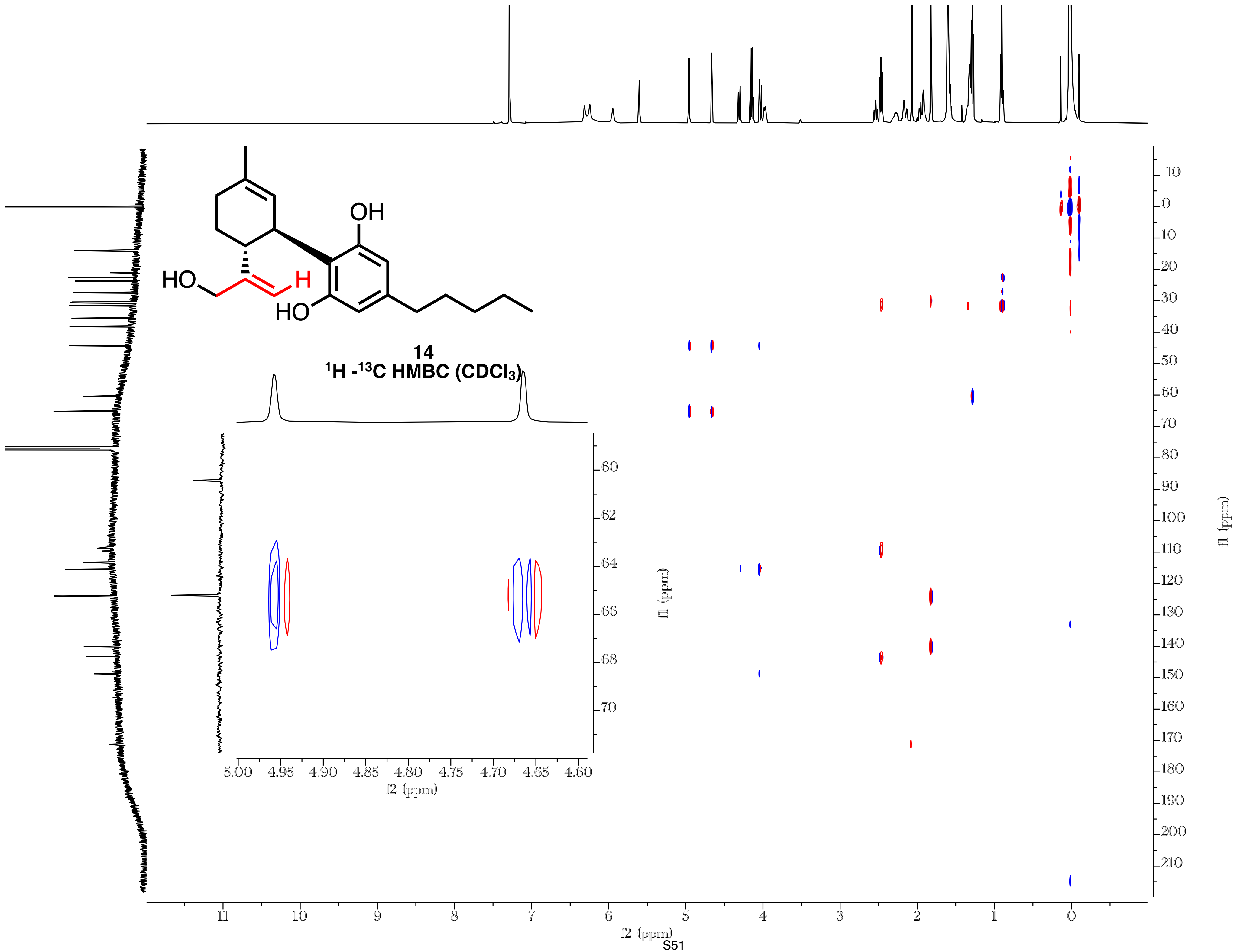
OH
HO
H
HO
14
^1H -^{13}C HMBC ($CDCl_3$)
f2 (ppm)
f1 (ppm)

S1

^1H NMR (500 MHz, $CDCl_3$)

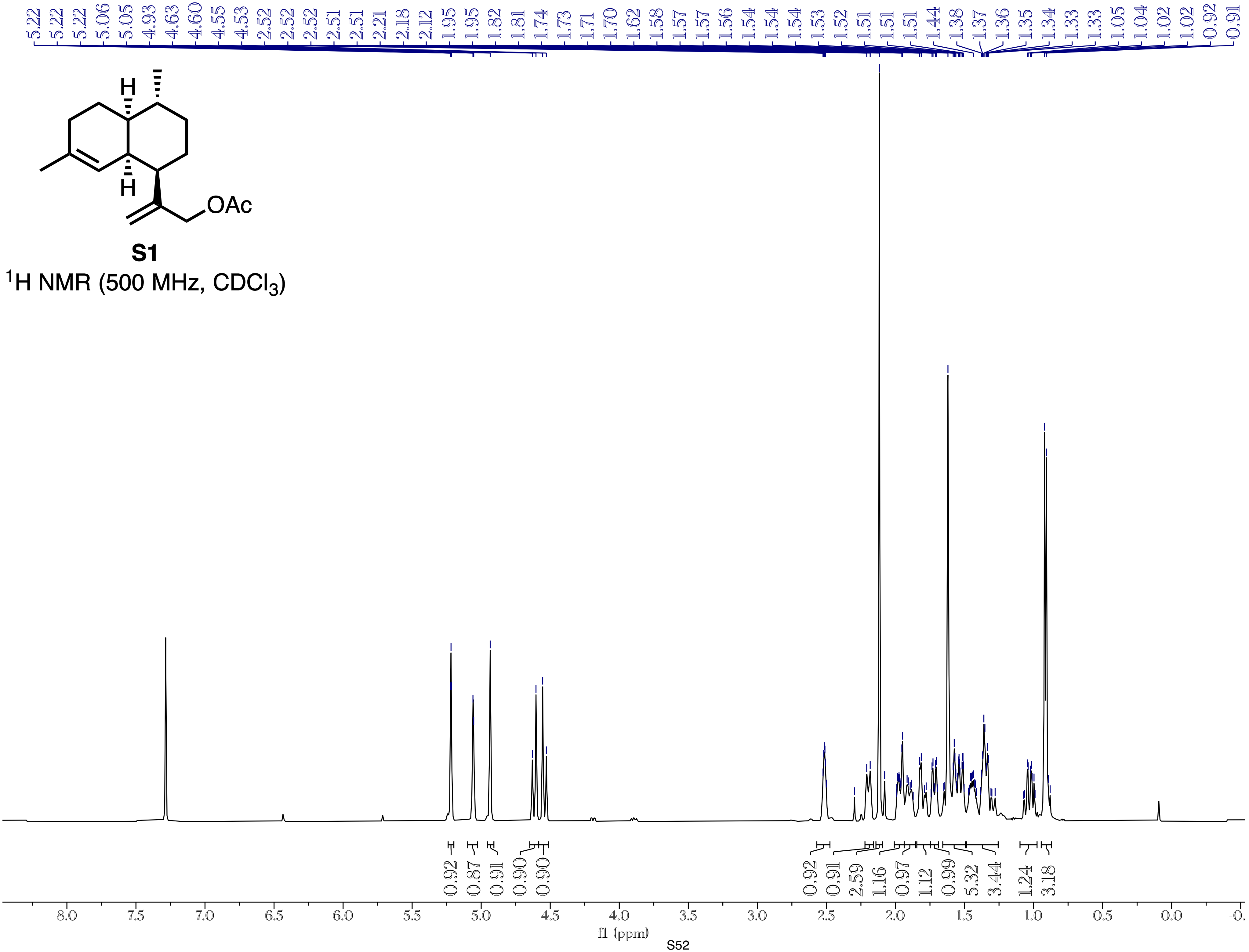

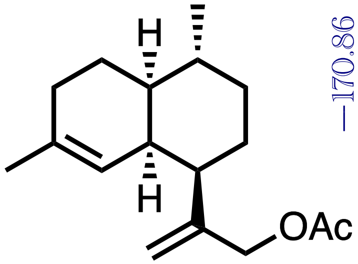

S1

^{13}C NMR (126 MHz, $CDCl_3$)

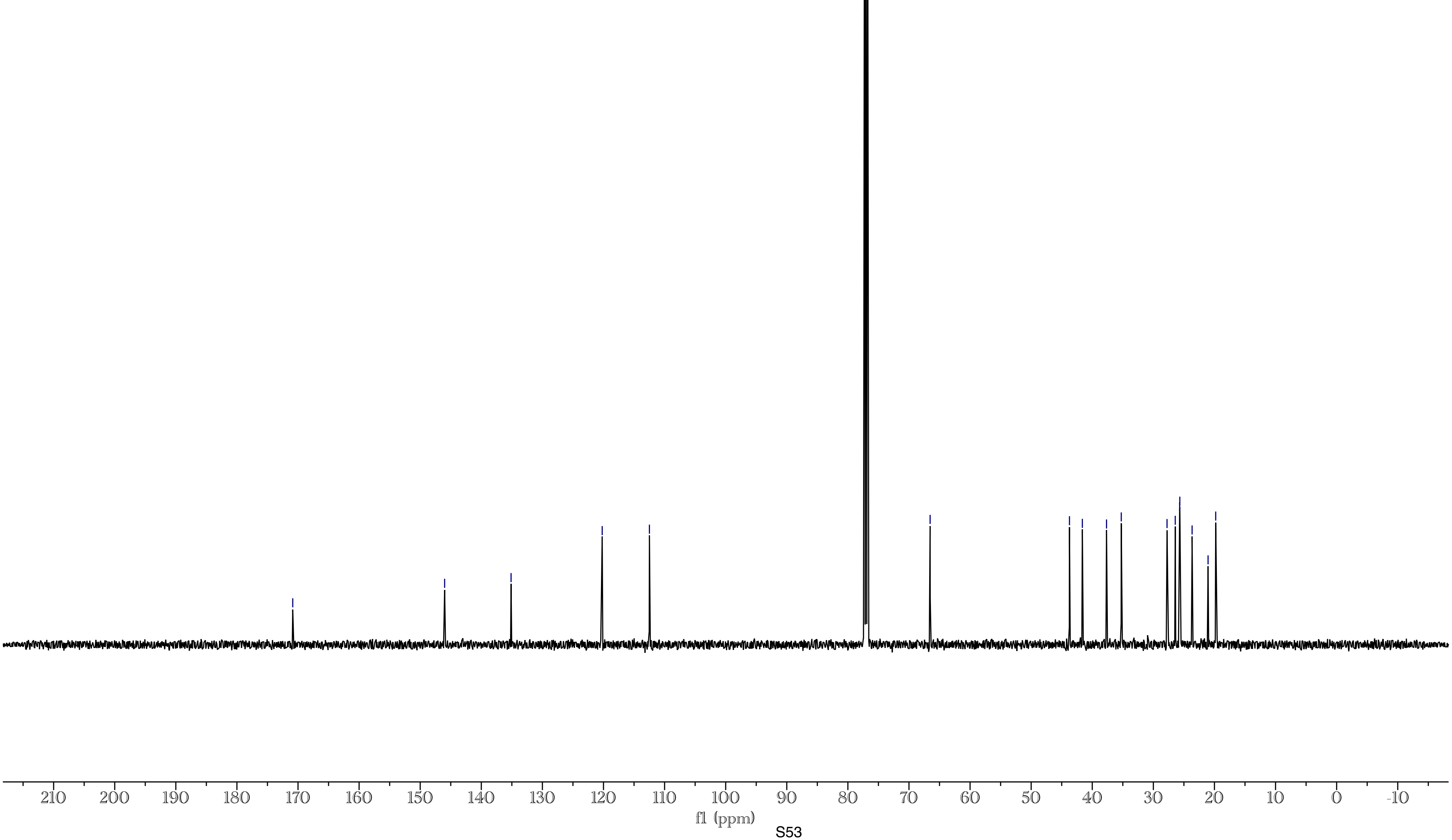

Supplement: Supplementary file 1 — ol2c04145_si_001.pdf [file ol2c04145_si_001.pdf]
